# Anodic electrosynthesis of MIL-53(Al)-N(CH_2_PO_3_H_2_)_2_ as a mesoporous catalyst for synthesis of novel (*N*-methyl-pyrrol)-pyrazolo[3,4-*b*]pyridines via a cooperative vinylogous anomeric based oxidation

**DOI:** 10.1038/s41598-021-97801-7

**Published:** 2021-09-29

**Authors:** Sima Kalhor, Mahmoud Zarei, Mohammad Ali Zolfigol, Hassan Sepehrmansourie, Davood Nematollahi, Saber Alizadeh, Hu Shi, Jalal Arjomandi

**Affiliations:** 1 Department of Organic Chemistry, Faculty of Chemistry, Bu-Ali Sina University, PO Box 6517838683, Hamedan, Iran; 2 Department of Analytical Chemistry, Faculty of Chemistry, Bu-Ali Sina University, PO Box 6517838683, Hamedan, Iran; 3 School of Chemistry and Chemical Engineering, Institute of Molecular Science, Shanxi University, Taiyuan, 030006 China; 4 Department of Physical Chemistry, Faculty of Chemistry, Bu-Ali Sina University, PO Box 6517838683, Hamedan, Iran

**Keywords:** Chemistry, Materials science, Nanoscience and technology

## Abstract

In this paper, the MIL-53(Al)-NH_2_ metal–organic frameworks (MOFs) was prepared based on the anodic electrosynthesis under green conditions. The anodic electrosynthesis as an environmentally friendly procedure was performed in the aqueous solution, room temperature, atmospheric pressure, and in the short reaction time (30 min). Also, the employed procedure was accomplished without the need for the ex-situ salt and base/probase additives as cation source and ligand activating agent at the constant current mode (10.0 mA cm^−2^). The electrosynthesized MOFs was functionalized with phosphorus acid tags as a novel mesoporous catalyst. This mesoporous catalyst was successfully employed for synthesis of new series (*N*-methyl-pyrrol)-pyrazolo[3,4-*b*]pyridines by one-pot condensation reaction of 3-methyl-1-phenyl-1*H*-pyrazol-5-amine, 3-(1-methyl-1*H*-pyrrol-2-yl)-3-oxopropanenitrile and various aromatic aldehydes (mono, bis and tripodal). This catalyst proceeded the organic synthetic reaction via a cooperative vinylogous anomeric based oxidation mechanism with a marginal decreasing its catalytic activity after recycling and reusability.

## Introduction

Metal–organic frameworks (MOFs) have been employed in various fields such as catalysts, electrocatalysts, supercapacitors, drug delivery, sensors, batteries, membranes, and absorbents due to the eye-catching features such as various synthesis methods, structural diversity, high surface area, and attractive architectures^1–14^. Besides, the potential functionalization and post-modification of MOFs have exposed the art of chemists in different fields^15,16^. The various documents have been reported for synthesis and electrosynthesis of MOFs as powder and thin films^17–29^. Behind the beneficial chemical synthesis procedures, the anodic and cathodic electrosynthesis methods are easy to observe and have been received a more acceptable niche and platform because of the environmentally friendly conditions^30–34^. This prominent glance comes from the one-step, green, and mild strategy that use of renewable electricity source instead of high temperature and high vacuum/pressure as a driving agent^30–34^. One of the MOFs having been synthesized via chemical and electrochemical procedures is MIL-53-(Al)-NH_2_, which has been used in different applications^35–41^. The main drawbacks of conventional chemical methods are the critical synthesis conditions such as high-pressure or -vacuum, prolonged time, and high temperature^38–41^. While, the anodic electrosynthesis method provides a mild and greener condition for this purpose^35–37^. In this method the anodic electrode plays the role of cation source for avoiding using of related cation salt which leads to the prevention of pore-blocking by the salt ions. Also, the electroreduction of water molecules on the counter electrode generates the hydroxide ions that lead to the in-situ deprotonation of ligands without the need for any ex-situ base/probase additive. Finally, the coordination and crystallization of MOF can be progressed by a green electricity source instead of high vacuum/pressure as a driving agent^30–34^. At the next step, the electrosynthesized MOFs was functionalized by phosphorous acid tags. Organphosphoric acid as a catalyst has been used for the oligomerization of light olefins, destructive alkylation of hydrocarbons and organic synthesis^42,43^. Furthermore, phosphoric acid and its derivatives have been also used in multinuclear NMR, adsorption and extraction^44^. Materials containing N(CH_2_PO_3_H_2_)_2_ moieties have environmental impacts worldwide in agricultural, chemical, and pharmaceutical applications^45,46^. Recently efficient methodologies have been reported for the preparation of metal–organic frameworks^47,48^, melamine and glycoluril^49,50^ and mesoporous materials (SBA-15) with phosphorous acid functional groups^51^.

**Figure 1:**
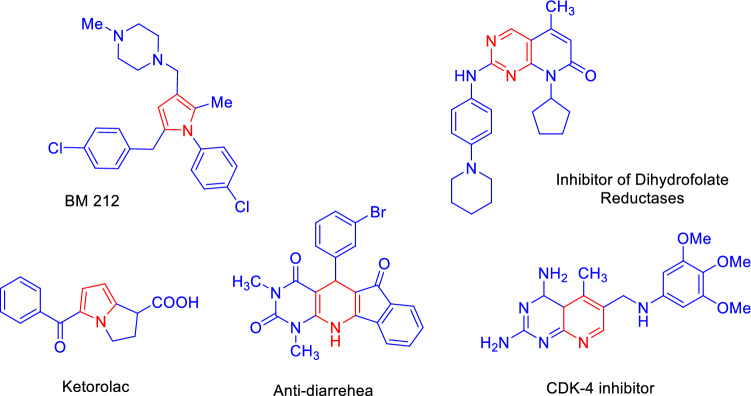
Structure of *N*-heterocycle rings as drug candidates.

Lots of researches have been done to achieve a novel pyridine and other derivatives as drug candidates^52,53^. Aryl and heterocyclic compounds substituted with *β*-ketonitriles have shown a unique class of useful intermediates. Pyran, pyridine and other ring derivatives plays important role in nature and drug development. Molecules with pyran and pyridine moieties are also associated with several important biological and pharmacological activities such as antitumor, antipyretic, analgesic, antiallergic, anti-hypertension, bronchitis dilator, strengthen the heart, blood vessel dilator, antimalarial, anti-fungal, an adenosine kinase inhibitor, diuretic, tyrosine kinas' inhibitors and antibacterial activity^54–59^. Furthermore, these materials were also applied in the synthesis of BM 212, Ketorolac and other compounds as anti‐fungal and anti‐bacterial, and anti‐inflammatory inhibitors of dihydrofolate reductases, anti-diarrhoea and CDK-4 inhibitor respectively (Fig. 1)^60^.

**Figure 2:**
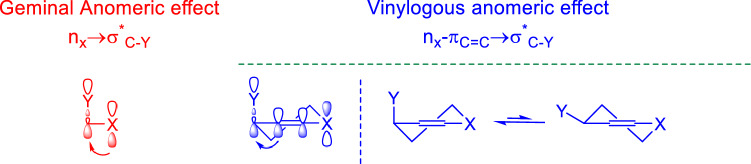
Geminal anomeric effect versus vinylogous anomeric effect^61^.

The anomeric interactions extended through double bonds has been named as a vinylogous anomeric effect. The geminal anomeric effect is compared with vinylogous anomeric (Fig. 2). As it can be seen in Fig. 2, in the vinylogous anomeric effect electron density is shared through a double bond but in the geminal anomeric effect donor and acceptor are germinal^61^. Recently anomeric based oxidation has been introduced for the latter step at the synthesis of susceptible molecules^62,63^. Since that reduction of substrates by NADH and NADPH_2_ have proceeded via a hydride transfer^64–66^. The key feature of the reduction mechanism is hydride transfer from carbon via an anomeric based oxidation mechanism. Herein, two cooperative vinylogous anomeric based oxidation mechanisms were suggested for the synthesis of new molecules with pyridine structure in the presence and absence of oxygen^67,68^.

**Figure 3:**
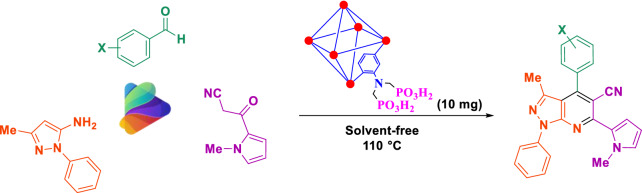
Synthesis of new (*N*-methyl-pyrrol)-pyrazolo[3,4-*b*]pyridines using MIL-53(Al)-N(CH_2_PO_3_H_2_)_2_.

Since molecules with indole moieties are biological interest candidates^69^ and our background on the comprehensive reviewing of bis and tris indolyl methanes^70^, we decided to synthesis pyridines with both indole and pyrazole moieties. Therefore, in continuation of our investigations on the development of electrosynthesis of metal–organic frameworks (MOFs) and its chemical post-functionalization with phosphorous acid tags^47–51^, herein we wish to report a green methodology for preparing of new MIL-53(Al)-N(CH_2_PO_3_H_2_)_2_ as an efficient mesoporous catalyst. For this purpose, the MIL-53-(Al)-NH_2_ was successfully fabricated via anodic electrosynthesis technique in the aqueous solution, short reaction time (30 min), room temperature, and atmospheric pressure at the constant current mode (10.0 mA cm^−2^). It should be noted that the employed procedure was accomplished without the need for the related cation salt as a cation source and ex-situ base/probase additive for deprotonation of the ligand. The final mesoporous catalyst was prepared through functionalizing of MIL-53-(Al)-NH_2_ with the phosphorous acid tags and utilized for the synthesis of new (*N*-methyl-pyrrol)-pyrazolo[3,4-*b*]pyridines by the condensation reaction of 3-methyl-1-phenyl-1*H*-pyrazol-5-amine, 3-(1-methyl-1*H*-pyrrol-2-yl)-3-oxopropanenitrile and various aromatic aldehydes (mono, bis and tripodal) under green conditions (Fig. 3).

## Experimental

### Materials

2-Amino terephthalic acid (NH_2_-H_2_BDC) (Merck, 95%), potassium nitrate (KNO_3_) (Sigma-Aldrich, 99%), DMF (Merck, 99%), phosphorous acid (Merck, 99%), 1-methyl-1*H*-pyrrole (Merck, 98%), 2-cyanoacetic acid (Merck, 98%), EtOH (Merck, 99%) were reagent-grade materials and employed as received without further purification.

### Instrumental measurements

From the model of the BRUKER Ultrashield FT-NMR spectrometer (δ in ppm) were recorded ^1^H NMR (600 or 400 MHz), ^13^C NMR (151 or 101 MHz). Recorded on a Büchi B-545 apparatus in open capillary tubes were melting points. The PerkinElmer PE-1600-FTIR device was recorded for infrared spectra of compounds. SEM was performed using a scanning electron microscope for field publishing made by TE-SCAN. Thermal gravimetry (TG) and differential thermal gravimetric (DTG) were analyzed by a Perkin Elmer (Model: Pyris 1). BET and BJH were analyzed by BELSORP-mini ii high precision Surface area and pore size. Xrd was analyzed by ITAL STRUCTURE APD2000.

### General procedure for the anodic electrosynthesis of MIL-53(Al)-NH_2_

In a typical anodic electrosynthesis procedure, (0.1 mmol, 0.127 g) potassium nitrate (KNO_3_) as a supporting electrolyte was dissolved in 45.0 mL distilled water (Solution A). Also, (8.2 mmol, 1.5 g) of 2-amino terephthalic acid (NH_2_-H_2_BDC) as a ligand was dissolved in the 5.0 mL EtOH (solution B) and added to solution A (10 Vol% EtOH). The precursor was stirred at room temperature for 15 min before the electrosynthesis. In the following, the prepared precursor transferred to the homemade undivided two-electrode cell consists of a cap glass bottle and two Aluminum plates (100.0 mm × 30.0 mm × 2.0 mm) as working (cation source) and the auxiliary electrodes. Electrosynthesis of MOF was performed by applying 10.0 mA cm^−2^ current densities for 30 min. The MIL-53-(Al)-NH_3_ powders were removed from the solution by centrifuge at 5000 rpm for 5 min and rinsed twice with distilled water and DMF. The final MIL-53-(Al)-NH_3_ was aged overnight at 100 °C.

### General procedure for the preparation of MIL-53(Al)-N(CH_2_PO_3_H_2_)_2_

**Figure 4:**
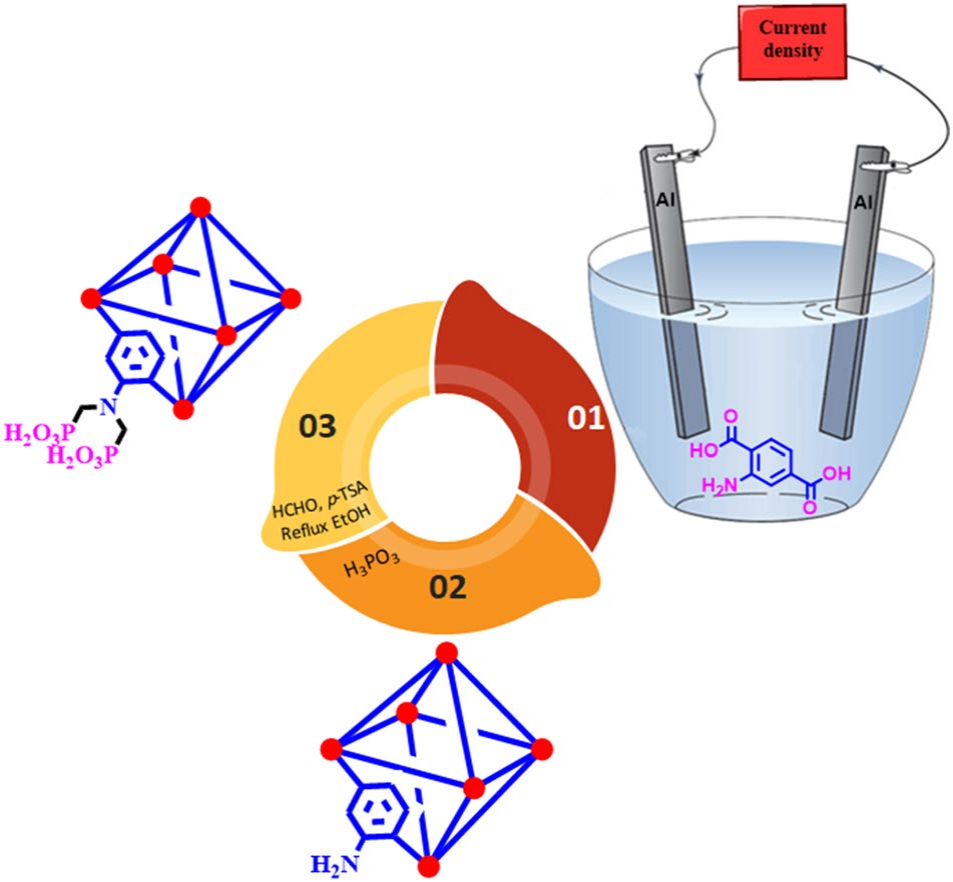
Preparation of MIL-53(Al)-N(CH_2_PO_3_H_2_)_2_.

In a 50 mL round-bottomed flask, MIL-53(Al)-NH_2_ (0.5 g), paraformaldehyde (5.0 mmol, 0.27 g), phosphorous acid (4.0 mmol, 0.76 g), *p-*TSA (0.5 mmol, 0.08 g) and ethanol (25 mL) were added and refluxed for 18 h. After this time, a yellow precipitated was filtered off by centrifugation (1000 rpm, 10 min). The obtained solid residue was dried under vacuum to give MIL-53(Al)-N(CH_2_PO_3_H_2_)_2_ (Fig. 4).

### General procedure for the synthesis of 3-methyl-6-(1-methyl-1*H*-pyrrol-3-yl)-1,4-diphenyl-1*H*-pyrazolo[3,4-b]pyridine-5-carbonitrile derivatives using MIL-53(Al)-N(CH_2_PO_3_H_2_)_2_ as the catalyst

**Figure 5:**
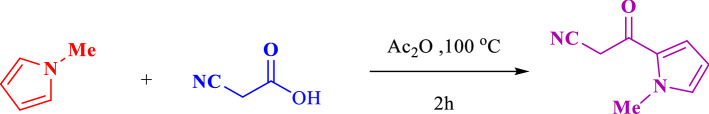
Preparation of 3-(1-methyl-1*H*-pyrrol-2-yl)-3-oxopropanenitrile.

At first 3-(1-methyl-1*H*-pyrrol-2-yl)-3-oxopropanenitrile was synthesized according to the previously reported literature (Fig. 5)^71^. Then, a mixture of 3-methyl-1-phenyl-1*H*-pyrazol-5-amine (1.0 mmol, 0.174 g), aryl aldehyde (1.0 mmol), 3-(1-methyl-1*H*-pyrrol-2-yl)-3-oxopropanenitrile (1.0 mmol, 0.148 g) and MIL-53(Al)-N(CH_2_PO_3_H_2_)_2_ (10.0 mg) as catalyst were stirred under solvent-free conditions at 110 °C in a 25.0 mL round-bottomed. After completion of the reaction (monitor by TLC *n-*hexane: ethyl acetate; 7:3). The catalyst was separated by centrifugation (2000 rpm) after adding PEG (5.0 mL) for 5 min. Finally, the mixture was filtered off and washed with ethanol (3 × 10 mL) and the final pure product was obtained (Fig. 3).

### Spectral data

#### 3-methyl-6-(1-methyl-1*H*-pyrrol-2-yl)-1-phenyl-4-(p-tolyl)-1*H*-pyrazolo[3,4-b] pyridine-5-carbonitrile (1a)

White solid; M.p: 216–218 °C; FT-IR (KBr): υ (cm^−1^) = 3282, 2955, 2923, 2219, 1575, 1556, 1506, 1488, 1415. ^1^H NMR (600 MHz, DMSO-*d*_6_) δ 8.13 (d, *J* = 7.8 Hz, 2H), 7.60 (t, *J* = 7.9 Hz, 2H), 7.54 (d, *J* = 7.9 Hz, 2H), 7.45 (d, *J* = 7.8 Hz, 2H), 7.40 (t, *J* = 7.4 Hz, 1H), 7.13 (s, 1H), 6.99 (dd, *J* = 3.8, 1.4 Hz, 1H), 6.21 (dd, *J* = 3.6, 2.7 Hz, 1H), 3.94 (s, 3H), 2.46 (s, 3H), 2.05 (s, 3H). ^13^C NMR (151 MHz, DMSO-*d*_6_) δ 153.4, 152.4, 149.7, 144.40, 140.0, 138.5, 131.3, 129.7, 129.5, 129.3, 129.0, 128.6, 127.0, 121.8, 118.4, 115.9, 112.5, 108.1, 101.0, 39.5, 36.9, 21.4, 14.9, (See SI*,* Figs. S1–S3).

#### 4-(4-isopropylphenyl)-3-methyl-6-(1-methyl-1*H*-pyrrol-2-yl)-1-phenyl-1*H*-pyrazolo[3,4-b] pyridine-5-carbonitrile (2a)

White solid; M.p: 193–195 °C; FT-IR (KBr): υ (cm^−1^) = 3067, 2959, 2929, 2866, 2221, 1572, 1558, 1541, 1507, 1415. ^1^H NMR (600 MHz, DMSO-*d*_6_) δ 8.13 (d, J = 7.7 Hz, 2H), 7.60 (t, J = 7.9 Hz, 2H), 7.57 (d, J = 8.1 Hz, 2H), 7.51 (d, J = 8.1 Hz, 2H), 7.40 (t, J = 7.4 Hz, 1H), 7.13 (s, 1H), 6.99 (dd, J = 3.8, 1.5 Hz, 1H), 6.21 (dd, J = 3.7, 2.7 Hz, 1H), 3.94 (s, 3H), 3.05 (d, J = 6.9 Hz, 1H), 2.04 (s, 3H), 1.31 (d, J = 6.9 Hz, 6H). ^13^C NMR (151 MHz, DMSO-*d*_6_) δ 153.3, 152.5, 150.7, 149.7, 144.4, 138.5, 131.5, 129.7, 129.5, 129.0, 128.6, 127.0, 126.8, 121.8, 118.4, 115.9, 112.6, 108.1, 100.9, 36.9, 33.7, 24.2, 14.8, (See SI*,* Figs. S4–S6).

#### 4-(4-methoxyphenyl)-3-methyl-6-(1-methyl-1*H*-pyrrol-2-yl)-1-phenyl-1*H*-pyrazolo[3,4-b] pyridine-5-carbonitrile (3a)

White solid; M.p: 208–210 °C; FT-IR (KBr): υ (cm^−1^) = 3018, 2974, 2933, 2838, 2119, 1609, 1577, 1558, 1513, 1437, 1415. ^1^H NMR (600 MHz, DMSO-*d*_6_) δ 8.12 (d, *J* = 7.9 Hz, 2H), 7.59 (d, *J* = 8.1 Hz, 3H), 7.39 (t, *J* = 7.4 Hz, 1H), 7.18 (d, *J* = 8.6 Hz, 2H), 7.12 (s, 1H), 6.99 (dd, *J* = 3.7, 1.3 Hz, 1H), 6.22–6.20 (m, 1H), 3.94 (s, 3H), 3.89 (s, 3H), 2.08 (s, 3H). ^13^C NMR (151 MHz, DMSO-*d*_6_) δ 160.9, 153.2, 152.5, 149.7, 144.4, 138.6, 131.1, 129.7, 128.9, 128.6, 127.0, 126.1, 121.7, 118.5, 115.9, 114.3, 112.7, 108.1, 101.1, 55.7, 36.9, 15.0, (See SI*,* Figs. S7–S9).

#### 4-(3,4-dimethoxyphenyl)-3-methyl-6-(1-methyl-1*H*-pyrrol-2-yl)-1-phenyl-1*H*-pyrazolo[3,4-b] pyridine-5-carbonitrile (4a)

White solid; M.p: 196–198 °C; FT-IR (KBr): υ (cm^−1^) = 3128, 2992, 2960, 2834, 2224, 1600, 1568, 1516, 1490, 1439. ^1^H NMR (400 MHz, DMSO-*d*_6_) δ 8.13 (d, *J* = 7.7 Hz, 2H), 7.61 (t, *J* = 7.9 Hz, 2H), 7.40 (t, *J* = 7.4 Hz, 1H), 7.28 (s, 1H), 7.20 (s, 2H), 7.13 (s, 1H), 7.00 (dd, *J* = 3.8, 1.4 Hz, 1H), 6.22 (dd, *J* = 3.7, 2.7 Hz, 1H), 3.94 (s, 3H), 3.89 (s, 3H), 3.83 (s, 3H), 2.13 (s, 3H). ^13^C NMR (101 MHz, DMSO-*d*_6_) δ 152.7, 152.0, 149.8, 149.2, 148.2, 144.0, 138.1, 129.2, 128.4, 128.1, 126.5, 125.7, 121.8, 121.3, 118.0, 115.4, 112.7, 112.2, 111.2, 107.6, 100.7, 55.7, 55.5, 36.4, 14.5, (See SI*,* Figs. S10–S12).

#### 3-methyl-6-(1-methyl-1*H*-pyrrol-2-yl)-1,4-diphenyl-1*H*-pyrazolo[3,4-b]pyridine-5 carbonitrile (5a)

Brown solid; M.p: 174–178 °C; FT-IR (KBr): υ (cm^−1^) = 2961, 2923, 2216, 1656, 1599, 1567, 1506, 1487, 1415. ^1^H NMR (400 MHz, DMSO-*d*_6_) δ 8.15–8.10 (m, 2H), 7.65–7.64 (m, 4H), 7.61–7.58 (m, 2H), 7.44–7.36 (m, 1H), 7.14 (dd, *J* = 2.6, 1.7 Hz, 1H), 7.00 (dd, *J* = 3.9, 1.7 Hz, 1H), 6.22 (dd, *J* = 3.9, 2.6 Hz, 1H), 3.95 (s, 3H), 2.02 (s, 3H). ^13^C NMR (101 MHz, DMSO-*d*_6_) δ 152.7, 151.9, 149.2, 143.8, 138.0, 133.7, 129.8, 129.2, 128.8, 128.5, 128.4, 128.1, 126.6, 121.3, 117.8, 115.5, 112.0, 107.6, 100.4, 36.4, 14.2, (See SI*,* Figs. S13–S15).

#### 4-(4-bromophenyl)-3-methyl-6-(1-methyl-1*H*-pyrrol-2-yl)-1-phenyl-1*H*-pyrazolo[3,4-b]pyridine-5-carbonitrile (6a)

White solid; M.p: 235–237 °C; FT-IR (KBr): υ (cm^−1^) = 3063, 2983, 2953, 2214, 1995, 1574, 1557, 1506, 1485, 1412. ^1^H NMR (600 MHz, DMSO-*d*_6_) δ 8.12 (d, *J* = 7.9 Hz, 2H), 7.86 (d, *J* = 8.3 Hz, 2H), 7.64–7.59 (m, 4H), 7.40 (t, *J* = 7.4 Hz, 1H), 7.14 (s, 1H), 7.00 (dd, *J* = 3.8, 1.4 Hz, 1H), 6.22–6.21 (m, 1H), 3.94 (s, 3H), 2.06 (s, 3H). ^13^C NMR (151 MHz, DMSO-*d*_6_) δ 152.4, 151.9, 149.7, 144.2, 138.5, 135.3, 133.0, 131.4, 129.7, 129.1, 129.1, 128.5, 127.1, 121.8, 118.2, 116.0, 112.4, 108.2, 100.9, 39.5, 36.9, 14.8, (See SI*,* Figs. S16–S18).

#### 4-(4-chlorophenyl)-3-methyl-6-(1-methyl-1*H*-pyrrol-2-yl)-1-phenyl-1*H*-pyrazolo[3,4-b]pyridine-5-carbonitrile (7a)

White solid; M.p: 238–240 °C; FT-IR (KBr): υ (cm^−1^) = 3083, 2980, 2964, 2216, 1595, 1573, 1555, 1506, 1486, 1413. ^1^H NMR (600 MHz, DMSO-*d*_6_) δ 8.12 (d, *J* = 7.8 Hz, 2H), 7.71 (q, *J* = 8.6 Hz, 4H), 7.61 (t, *J* = 7.9 Hz, 2H), 7.40 (t, *J* = 7.4 Hz, 1H), 7.14 (s, 1H), 7.00 (dd, *J* = 3.8, 1.5 Hz, 1H), 6.22 (dd, *J* = 3.7, 2.7 Hz, 1H), 3.94 (s, 3H), 2.06 (s, 3H). ^13^C NMR (151 MHz, DMSO-*d*_6_) δ 152.4, 151.9, 149.7, 144.2, 138.5, 135.3, 133.0, 131.4, 129.7, 129.1, 129.1, 128.5, 127.1, 121.8, 118.2, 116.0, 112.43, 108.2, 100.9, 36.9, 14.8, (See SI*,* Figs. S19–S21).

#### 4-(4-Fluorophenyl)-3-methyl-6-(1-methyl-1*H*-pyrrol-2-yl)-1-phenyl-1*H*-pyrazolo[3,4-b]pyridine-5-carbonitrile (8a)

Light yellow solid; M.p.: 241–243 °C; FT-IR (KBr): υ (cm^−1^) = 3070, 2960, 2226, 1602, 1563, 1506, 1486, 1412. ^1^H NMR (400 MHz, DMSO-*d*_6_) δ 8.13 (d, *J* = 7.8 Hz, 2H), 7.60 (t, *J* = 7.9 Hz, 2H), 7.54 (d, *J* = 7.9 Hz, 2H), 7.45 (d, *J* = 7.8 Hz, 2H), 7.40 (t, *J* = 7.4 Hz, 1H), 7.13 (s, 1H), 6.99 (dd, *J* = 3.8, 1.4 Hz, 1H), 6.21 (dd, *J* = 3.6, 2.7 Hz, 1H), 3.94 (s, 3H), 2.46 (s, 3H), 2.05 (s, 3H). ^13^C NMR (101 MHz, DMSO-*d*_6_) δ 164.1, 161.7, 151.9, 151.75, 149.2, 143.8, 138.0, 131.4, 131.4, 130.0, 129.3, 128.6, 128.0, 126.6, 121.3, 117.83, 115.7, 115.5, 115.4, 112.1, 107.7, 100.6, 36.4, 14.3, (See SI*,* Figs. S22–S24).

#### 3-Methyl-6-(1-methyl-1*H*-pyrrol-2-yl)-1-phenyl-4-(pyridin-4-yl)-1*H*-pyrazolo[3,4-b]pyridine-5-carbonitrile (9a)

Pale orange solid; M.p: 218–220 °C; FT-IR (KBr): υ (cm^−1^) = 3041, 2958, 2233, 1596, 1568, 1554, 1540, 1505, 1413. ^1^H NMR (400 MHz, DMSO-*d*_6_) δ 8.87 (dd, *J* = 4.4, 1.6 Hz, 2H), 8.14–8.09 (m, 2H), 7.72 (dd, *J* = 4.4, 1.6 Hz, 2H), 7.61 (t, *J* = 8.0 Hz, 2H), 7.41 (t, *J* = 7.4 Hz, 1H), 7.17–7.14 (m, 1H), 7.02 (dd, *J* = 3.9, 1.7 Hz, 1H), 6.23 (dd, *J* = 3.9, 2.6 Hz, 1H), 3.95 (s, 3H), 2.04 (s, 3H). ^13^C NMR (101 MHz, DMSO-*d*_6_) δ 151.8, 149.8, 149.6, 149.1, 143.5, 141.7, 137.9, 129.3, 129.2, 128.8, 127.9, 126.7, 123.5, 121.3, 119.9, 117.4, 115.6, 111.2, 107.8, 99.81, 36.5, 14.1, (See SI*,* Figs. S25–S27).

#### 4-(4-(Dimethylamino)phenyl)-3-methyl-6-(1-methyl-1*H*-pyrrol-2-yl)-1-phenyl-1*H*-pyrazolo[3,4-b]pyridine-5-carbonitrile (10a)s

Yellow solid; M.p: 248–250 °C; FT-IR (KBr): υ (cm^−1^) = 3107, 2906, 2814, 2220, 1605, 1530, 1504, 1486. ^1^H NMR (400 MHz, DMSO-*d*_6_) δ 8.16–8.07 (m, 2H), 7.59 (t, *J* = 8.0 Hz, 2H), 7.51–7.44 (m, 2H), 7.43–7.34 (m, 1H), 7.10 (t, *J* = 2.1 Hz, 1H), 6.97 (dd, *J* = 3.9, 1.7 Hz, 1H), 6.93–6.86 (m, 2H), 6.20 (dd, *J* = 3.9, 2.6 Hz, 1H), 3.92 (s, 3H), 3.04 (s, 6H), 2.16 (s, 3H), (See SI*,* Figs. S28–S29).

#### 4-(4-(Diethylamino)phenyl)-3-methyl-6-(1-methyl-1*H*-pyrrol-2-yl)-1-phenyl-1*H*-pyrazolo[3,4-b]pyridine-5-carbonitrile (11a)

Orange solid; M.p: 186–188 °C; FT-IR (KBr): υ (cm^−1^) = 2969, 2894, 2870, 2213, 1604, 1568, 1521, 1506, 1484. ^1^H NMR (400 MHz, DMSO-*d*_6_) δ 8.12 (d, *J* = 7.7 Hz, 2H), 7.59 (t, *J* = 7.9 Hz, 2H), 7.45 (d, *J* = 8.8 Hz, 2H), 7.38 (t, *J* = 7.4 Hz, 1H), 7.11–7.09 (m, 1H), 6.97 (dd, *J* = 3.9, 1.6 Hz, 1H), 6.84 (d, *J* = 8.9 Hz, 2H), 6.20 (dd, *J* = 3.8, 2.6 Hz, 1H), 3.92 (s, 3H), 3.45 (q, *J* = 6.9 Hz, 4H), 2.19 (s, 3H), 1.17 (t, *J* = 7.0 Hz, 6H). ^13^C NMR (101 MHz, DMSO-*d*_6_) δ 153.5, 152.3, 149.4, 148.5, 144.0, 138.1, 130.9, 129.2, 128.3, 128.1, 126.4, 121.2, 118.9, 118.6, 115.3, 112.1, 110.3, 107.5, 100.1, 43.6, 36.3, 15.0, 12.3, (See SI*,* Figs. S30–S32).

#### 4-(1*H*-Indol-3-yl)-3-methyl-6-(1-methyl-1*H*-pyrrol-2-yl)-1-phenyl-1*H*-pyrazolo[3,4-b]pyridine-5-carbonitrile (12a)

Light brown solid; M.p: 127–129 °C; FT-IR (KBr): υ (cm^−1^) = 3414, 3114, 3062, 2971, 2930, 2218, 1618, 1596, 1558, 1507, 1486, 1415. ^1^H NMR (400 MHz, DMSO-*d*_6_) δ 11.94 (s, 1H), 8.19–8.11 (m, 2H), 7.96 (s, 1H), 7.60 (t, *J* = 8.0 Hz, 3H), 7.42 (dd, *J* = 16.1, 7.7 Hz, 2H), 7.24 (t, *J* = 7.2 Hz, 1H), 7.17–7.10 (m, 2H), 6.99 (dd, *J* = 3.9, 1.7 Hz, 1H), 6.21 (dd, *J* = 3.9, 2.6 Hz, 1H), 3.96 (s, 3H), 2.07 (s, 3H). ^13^C NMR (101 MHz, DMSO-*d*_6_) δ 152.4, 149.5, 147.2, 144.2, 138.1, 135.9, 129.2, 128.2, 127.6, 126.5, 126.2, 122.1, 121.3, 120.2, 119.2, 118.6, 115.3, 113.0, 112.2, 107.9, 107.5, 101.3, 36.35, 14.3, (See SI*,* Figs. S33–S35).

#### 3-Methyl-6-(1-methyl-1*H*-pyrrol-2-yl)-4-(4-nitrophenyl)-1-phenyl-1*H*-pyrazolo[3,4-b]pyridine-5-carbonitrile (13a)

Brown solid; M.p: 179–181 °C; FT-IR (KBr): υ (cm^−1^) = 3071, 2952, 2220, 1598, 1567, 1557, 1507, 1439, 1415. ^1^H NMR (400 MHz, DMSO-*d*_6_) δ 8.49 (d, *J* = 8.3 Hz, 1H), 8.13 (t, *J* = 7.7 Hz, 3H), 7.98 (d, *J* = 8.1 Hz, 1H), 7.61 (s, 2H), 7.43–7.37 (m, 2H), 7.15 (s, 1H), 7.05–7.01 (m, 1H), 6.23 (s, 1H), 3.95 (s, 3H), 2.02 (s, 3H). ^13^C NMR (101 MHz, DMSO-*d*_6_) δ 151.8, 150.3, 149.1, 148.3, 143.6, 140.1, 138.0, 130.6, 130.3, 129.3, 129.2, 128.8, 126.7, 124.9, 123.6, 122.5, 121.34, 119.8, 117.5, 115.6, 107.8, 36.5, 14.3, (See SI*,* Figs. S36–S38).

#### 3-Methyl-6-(1-methyl-1*H*-pyrrol-2-yl)-4-(3-nitrophenyl)-1-phenyl-1*H*-pyrazolo[3,4-b]pyridine-5-carbonitrile (14a)

Yellow solid; M.p: 204–206 °C; FT-IR (KBr): υ (cm^−1^) = 3078, 2955, 2925, 2857, 2214, 1669, 1567, 1535, 1489, 1419. ^1^H NMR (600 MHz, DMSO-*d*_6_) δ 8.60 (s, 1H), 8.52–8.49 (m, 1H), 8.16 (d, *J* = 7.6 Hz, 1H), 8.13 (d, *J* = 7.7 Hz, 2H), 7.96 (t, *J* = 8.0 Hz, 1H), 7.62 (t, *J* = 7.9 Hz, 2H), 7.41 (t, *J* = 7.4 Hz, 1H), 7.15 (s, 1H), 7.03 (dd, *J* = 3.8, 1.5 Hz, 1H), 6.23 (dd, *J* = 3.7, 2.7 Hz, 1H), 3.95 (s, 3H), 2.03 (s, 3H). ^13^C NMR (151 MHz, DMSO-*d*_6_) δ 152.4, 151.9, 149.7, 144.2, 138.5, 135.3, 133.0, 131.4, 129.7, 129.1, 129.1, 128.5, 127.1, 121.8, 118.2, 116.0, 112.4, 108.2, 100.9, 36.9, 14.8, (See SI*,* Figs. S39–S41).

#### 4-(5-Cyano-3-methyl-6-(1-methyl-1*H*-pyrrol-2-yl)-1-phenyl-1*H*-pyrazolo[3,4-b]pyridin-4-yl)benzoic acid (15a)

Beige solid; M.p: 177–179 °C; FT-IR (KBr): υ (cm^−1^) = 3420, 3107, 2971, 2931, 2217, 1709, 1666, 1599, 1556, 1508, 1439, 1419. ^1^H NMR (400 MHz, DMSO-*d*_6_) δ 8.14–8.11 (m, 4H), 7.66 (d, *J* = 8.1 Hz, 2H), 7.61 (t, *J* = 7.9 Hz, 2H), 7.40 (t, *J* = 7.4 Hz, 1H), 7.15–7.11 (m, 1H), 7.01 (dd, *J* = 3.9, 1.6 Hz, 1H), 6.22 (dd, *J* = 3.8, 2.6 Hz, 1H), 3.95 (s, 3H), 2.03 (s, 3H). ^13^C NMR (101 MHz, DMSO-*d*_6_) δ 167.8, 152.9, 152.4, 149.7, 144.3, 138.5, 136.4, 129.7, 129.6, 129.1, 128.6, 127.1, 121.8, 118.2, 116.0, 112.3, 108.2, 100.7, 36.9, 14.7, (See SI*,* Figs. S42–S44).

#### 4,4'-(1,4-Phenylene)bis(3-methyl-6-(1-methyl-1*H*-pyrrol-2-yl)-1-phenyl-1*H*-pyrazolo[3,4-b]pyridine-5-carbonitrile) (16a)

Beige solid; dec.: 320 °C; FT-IR (KBr): υ (cm^−1^) = 3064, 2949, 2220, 1646, 1566, 1507, 1436, 1417. ^1^H NMR (400 MHz, DMSO-*d*_6_) δ ^1^H NMR (400 MHz, DMSO) δ 8.16 (d, *J* = 8.4 Hz, 4H), 7.93 (d, *J* = 8.7 Hz, 3H), 7.66–7.58 (m, 5H), 7.41 (d, *J* = 7.0 Hz, 2H), 7.16 (s, 2H), 7.07 (dd, *J* = 3.7, 1.4 Hz, 2H), 6.27–6.23 (m, 2H), 3.98 (s, 6H), 2.15 (s, 3H), 2.12 (s, 3H), (See SI*,* Figs. S45–S46).

#### 4,4'-(1,3-Phenylene)bis(3-methyl-6-(1-methyl-1*H*-pyrrol-2-yl)-1-phenyl-1*H*-pyrazolo[3,4-b]pyridine-5-carbonitrile) (17a)ss

Brown solid; M.p: 248–250 °C; FT-IR (KBr): υ (cm^−1^) = 3463, 2950, 2220, 1660, 1599, 1570, 1558, 1506, 1490, 1416. ^1^H NMR (400 MHz, DMSO-*d*_6_) δ 8.13 (d, *J* = 7.8 Hz, 4H), 7.91 (d, *J* = 7.9 Hz, 2H), 7.63–7.59 (m, 5H), 7.40 (t, *J* = 7.4 Hz, 3H), 7.14 (d, *J* = 1.9 Hz, 2H), 7.01 (dd, *J* = 3.9, 1.6 Hz, 2H), 6.22 (dd, *J* = 3.7, 2.7 Hz, 2H), 3.96 (s, 6H), 2.13 (s, 6H). ^13^C NMR (101 MHz, DMSO-*d*_6_) δ 151.8, 151.8, 149.1, 144.0, 138.0, 134.4, 129.9, 129.3, 129.0, 128.7, 128.5, 128.0, 126.6, 121.3, 117.7, 115.5, 112.1, 107.7, 100.6, 36.5, 14.0, (See SI*,* Figs. S47–S49).

#### 4,4',4''-(((1,3,5-Triazine-2,4,6-triyl)tris(oxy))tris(benzene-4,1-diyl))tris(3-methyl-6-(1-methyl-1H-pyrrol-2-yl)-1-phenyl-1H-pyrazolo[3,4-b]pyridine-5-carbonitrile) (18a)s

Brown solid; M.p: 131–133 °C; FT-IR (KBr): υ (cm^−1^) = 3118, 2928, 2877, 2806, 2217, 1662, 1612, 1593, 1561, 1516, 1487, 1439. ^1^H NMR (400 MHz, DMSO-*d*_6_) δ 8.15–8.09 (m, 6H), 7.60 (dd, *J* = 10.9, 5.1 Hz, 6H), 7.46 (d, *J* = 8.6 Hz, 6H), 7.39 (t, *J* = 7.4 Hz, 3H), 7.13–7.10 (m, 3H), 7.00 (d, *J* = 1.8 Hz, 3H), 6.98 (dd, *J* = 3.3, 2.1 Hz, 6H), 6.21 (dd, *J* = 3.9, 2.6 Hz, 3H), 3.93 (s, 9H), 2.11 (s, 9H). ^13^C NMR (101 MHz, DMSO-*d*_6_) δ 162.2, 159.1, 153.2, 152.0, 149.3, 144.0, 138.1, 130.7, 129.2, 129.0, 128.3, 128.2, 126.4, 123.8, 123.2, 121.2, 118.1, 115.4, 115.2, 112.2, 107.6, 100.5, 30.5, 14.4, (See SI*,* Figs. S50–S52).

## Results and discussion

**Figure 6:**
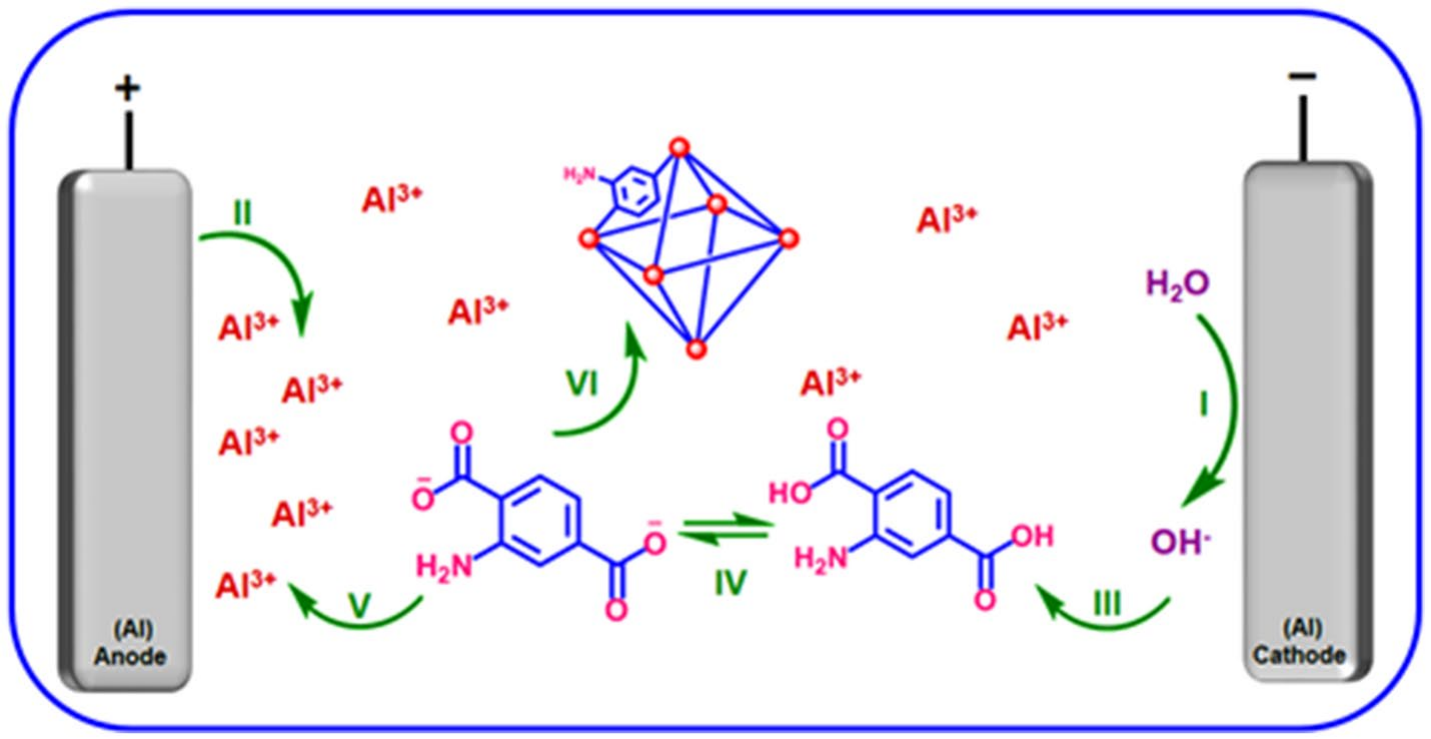
Preparation of MIL-53(Al)-NH_2_ using anodic electrosynthesis method.

In order to develop the catalytic application of metal–organic frameworks (MOFs), MIL-53(Al)-NH_2_ was prepared by anodic electrosynthesis technique (Fig. 6). Post-functionalization of MIL-53(Al)-NH_2_ for preparing of MIL-53(Al)-N(CH_2_PO_3_H_2_)_2_ was occurred by using paraformaldehyde under refluxing ethanol and presence *p*-TSA. To shed light of this issue, at the first step, we employed an anodic electrosynthesis procedure for the preparation of MIL-53(Al)-NH_2_. This mode has accomplished by inserting two aluminium metallic plates in the presence of H_2_BDC-NH_2_ and KNO_3_ as ligand and supporting electrolyte, respectively at room temperature in an EtOH/H_2_O (10/90, v/v) mixture under stirring. Aluminium metal plays the role of cation source and also the anodic and cathodic electrodes. Upon the establishment of the current density (10.0 mA cm^−2^ at 30 min), the increased pH at the cathode surface thanks to the in-situ electrogeneration of hydroxide ions makes deprotonation of ligands by electroreduction of water molecules. On the other hand, the anode surface starts to oxidize in order to the preparation of aluminum cations to coordinate with the available ligands. The activated ligands can be coordinated to the available cations to starting the crystallinity of MIL-53(Al)-NH_2_ (Fig. 6). This process has some advantages as in-situ deprotonation of ligand, in-situ cation electrogeneration, cation-controlled release, and avoiding of the pore-blocking due to the absence of the related cation salt. The MIL-53(Al)-NH_2_ has prepared without the need of any ex-situ base/probase and cation salt additive, at room temperature, atmospheric pressure, and short reaction time. At the second step, the post-modification of the prepared MIL-53(Al)-NH_2_ with the phosphorous acid tags was performed in the percent of *p*-TSA and paraformaldehyde under refluxing ethanol. To shed light of the more details, the full characterization of the MIL-53(Al)-NH_2_ and MIL-53(Al)-N(CH_2_PO_3_H_2_)_2_ as a porous material catalyst was evaluated by FT-IR, X-ray diffraction analysis (XRD), energy-dispersive X-ray spectroscopy (EDX), FE-SEM, elemental mapping, thermal gravimetric (TG) and derivative thermal gravimetric (DTG) techniques.

**Figure 7:**
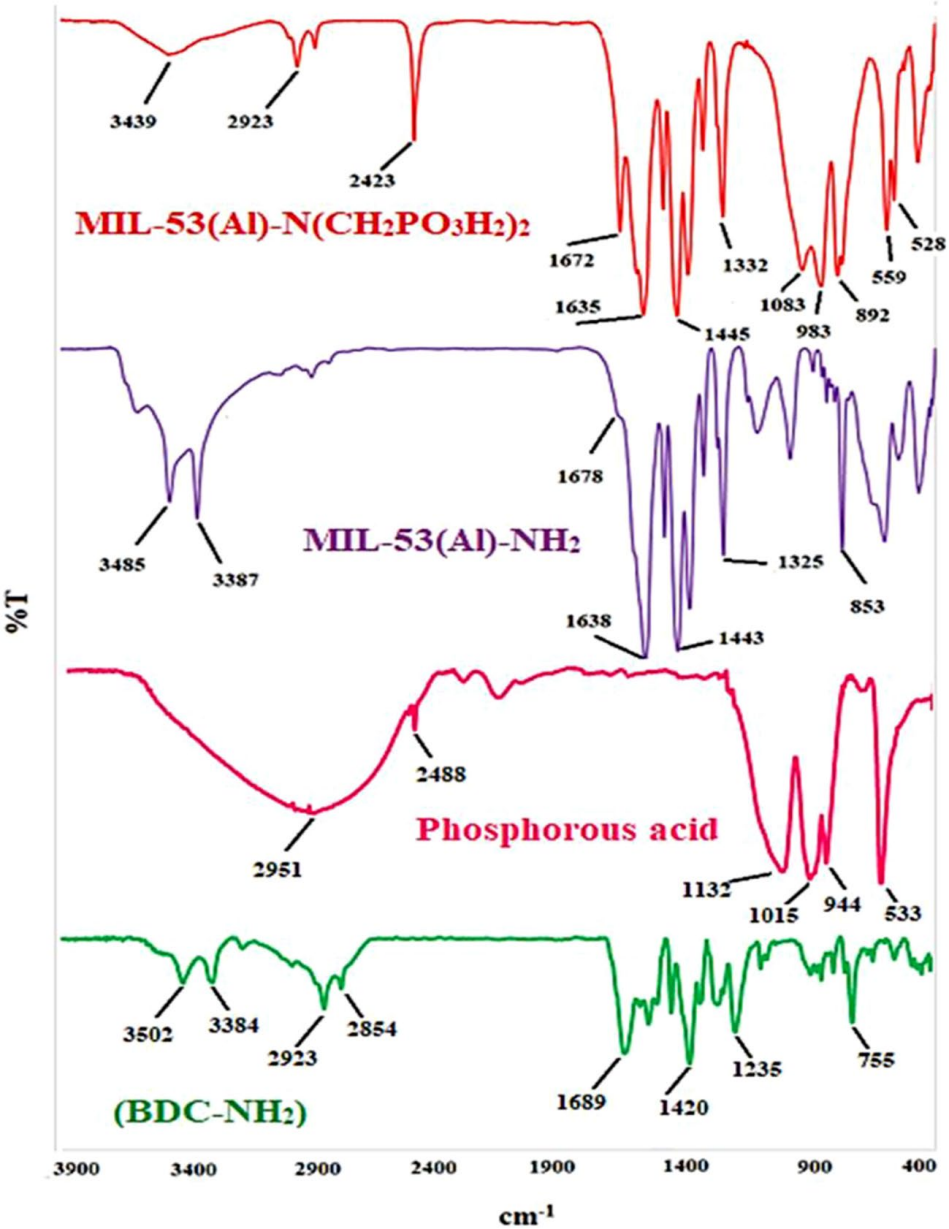
FT-IR analysis of 2-amino terephthalic acid, phosphorous acid, MIL-53(Al)-NH_2_ and MIL-53(Al)-N(CH_2_PO_3_H_2_)_2_.

The FT-IR analysis was surveyed for verification of the functional groups and bonding properties in the synthesized MOF as a catalyst. The FT-IR spectra of 2-amino terephthalic acid as susceptible ligand, phosphorus acid, MIL-53(Al)-NH_2_ and MIL-53(Al)-N(CH_2_PO_3_H_2_)_2_ MOFs were shown in Fig. 7. The broad peak 2600–3500 cm^−1^ is related to the OH of PO_3_H_2_ functional groups. Also, the absence of the OH group’s (carboxylic acid) peak 2488–3500 cm^−1^ in the ligand spectrum. Then, the absence of free C=O functional groups shifting of the characteristic couple bands from 1689 cm^−1^ in the ligand spectrum to the 1672 cm^−1^ in the MOFs spectra can be assigned to the coordination of the COO^–^ groups with the aluminium metallic cations. Besides, the presence of the two sharp bands at 3485 and 3387 cm^−1^ in the MIL-53(Al)-NH_2_ spectrum indicates the symmetric and asymmetric vibrations of the NH_2_ groups^35–37,72–78^. The absorption bands at 944 and 1015 cm^−1^ are related to P-O bond stretching and the band at 1132 cm^−1^ is related to P=O. The FT-IR spectrum difference between MIL-53(Al)-NH_2_ and MIL-53(Al)-N(CH_2_PO_3_H_2_)_2_ verified the structure of the desired post-synthesized catalyst.

**Figure 8:**
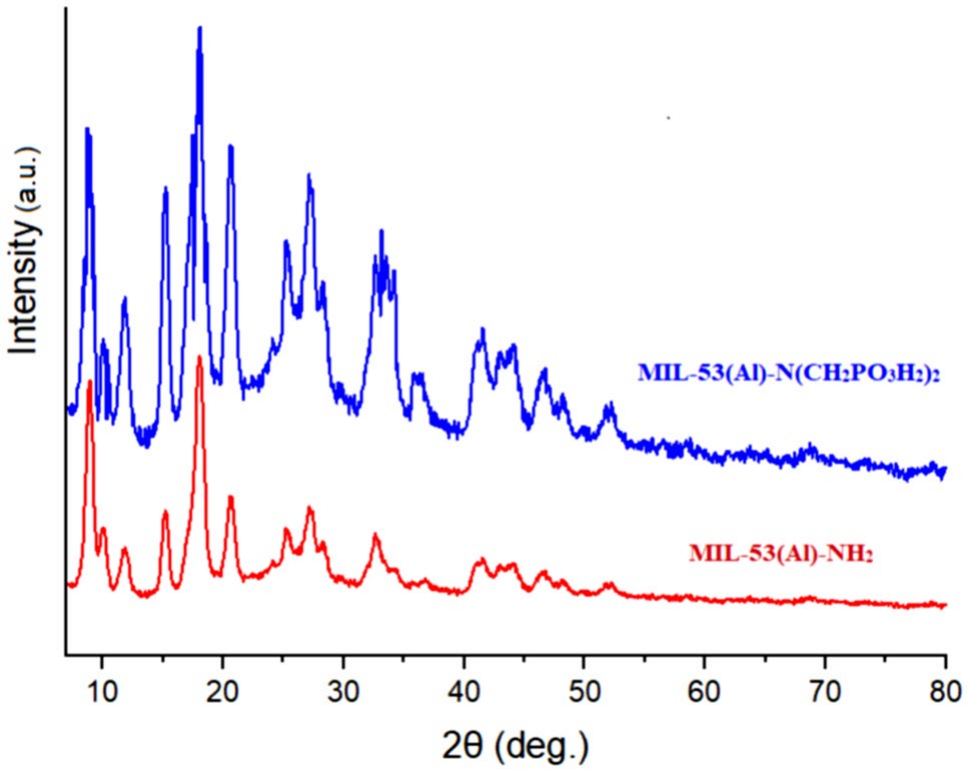
XRD pattern of MIL-53(Al)-NH_2_ and MIL-53(Al)-N(CH_2_PO_3_H_2_)_2_.

To study the purity and crystallinity of the synthesized MOF structures, the XRD patterns were recorded. According to the XRD profile (Fig. 8), the MIL-53(Al)-NH_2_ and MIL-53(Al)-N(CH_2_PO_3_H_2_)_2_ are isoreticualr materials with suitable crystallinity and without any impurity or contaminants. Comparing the three characteristic peaks located at the 2θ = 9.27°, 12.40°, and 17.50°, 25.10° with previously reported data is approved their structures^35–37,72–78^.

**Figure 9:**
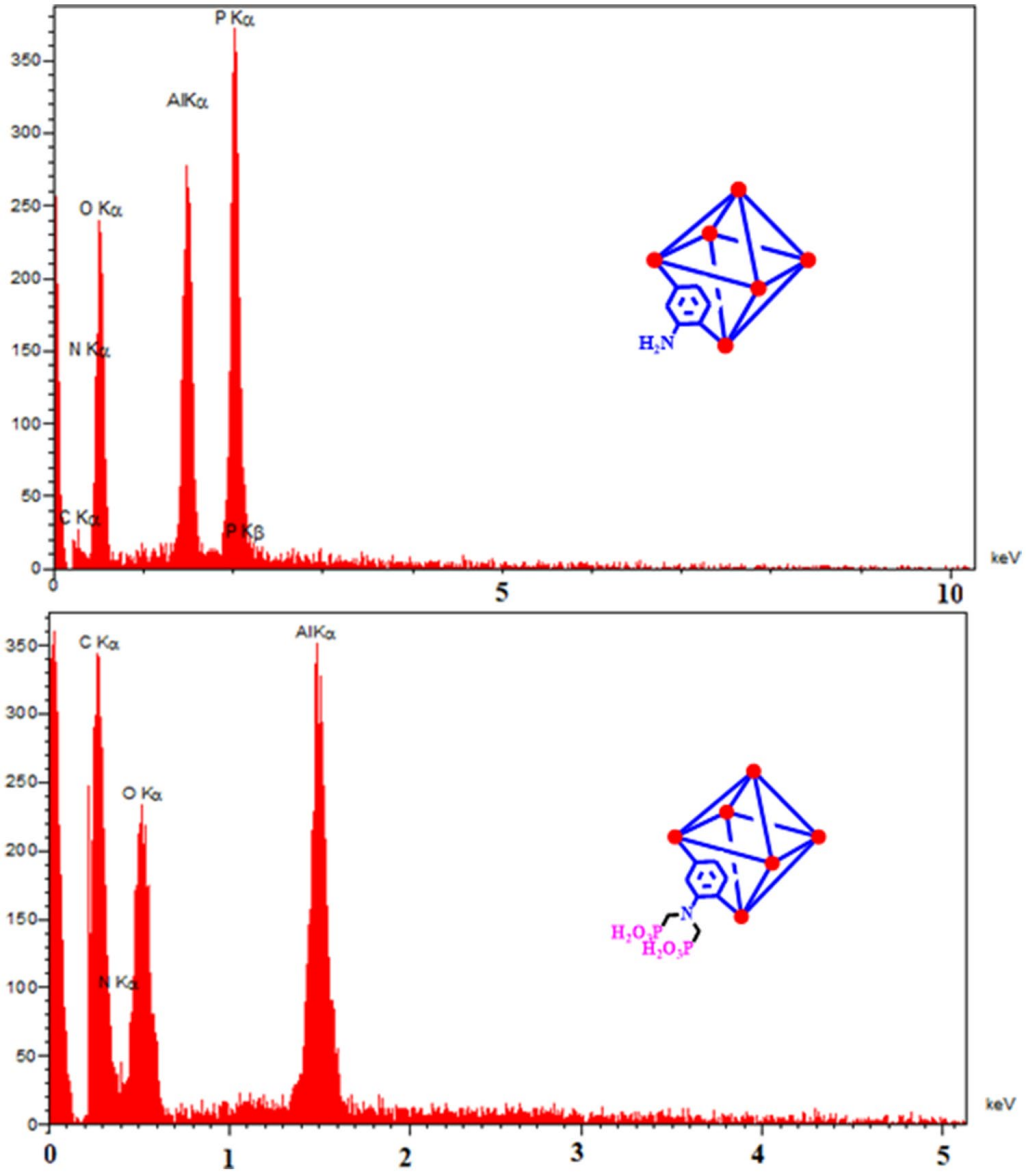
EDX analysis and elemental mapping of electro-synthesized MIL-53(Al)-NH_2_ and MIL-53(Al)-N(CH_2_PO_3_H_2_)_2_.

**Figure 10:**
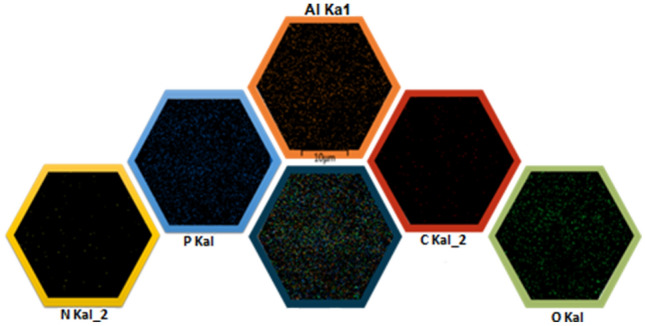
Elemental maps (EDX) of C (red); N (yellow); Al (orange); P (blue) and O (green) atoms for MIL-53(Al)-N(CH_2_PO_3_H_2_.

For more details, the EDX analysis was also accomplished for elemental analysis and elemental dispersity of prepared MOFs (Fig. 9). The emerged elemental peaks approved the existence of C, O, N, and Al elements in the MIL-53(Al)-NH_2_ structure and P element in the MIL-53(Al)-N(CH_2_PO_3_H_2_)_2_ structure. Besides, the EDX mapping images indicates the uniform and homogeneous distribution of elements (Fig. 10).

**Figure 11:**
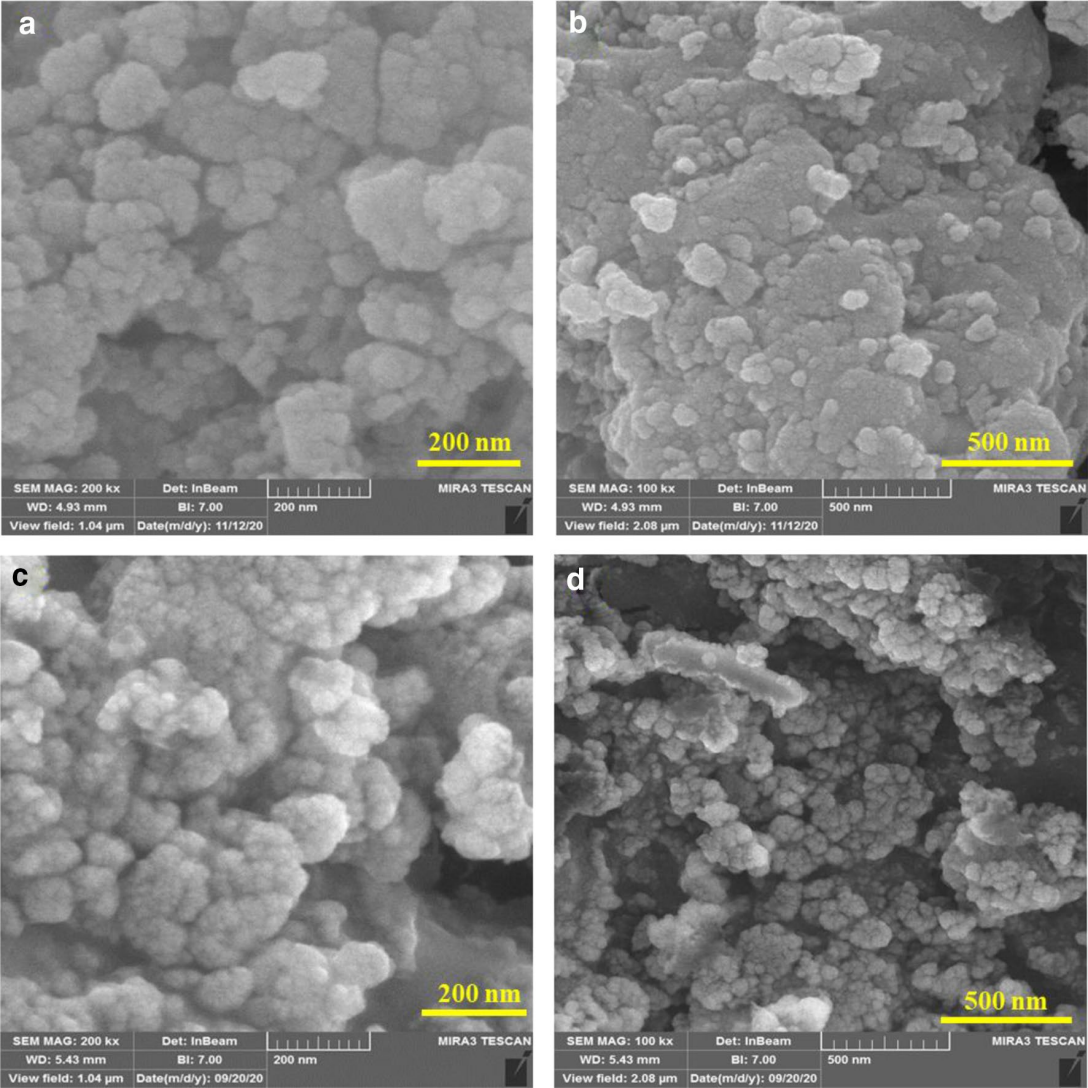
FE-SEM images of MIL-53(Al)-NH_2_ (**a**,**b**) and MIL-53(Al)-N(CH_2_PO_3_H_2_)_2_(**c**,**d**).

Also, FE-SEM images of electro-synthesized MIL-53(Al)-NH_2_ and its post-functionalized one were recorded for investigation of their morphologies (Fig. 11). The obtained images exhibit the uniform cauliflower-shaped nanoparticles with an average diameter size of around 33.0 nm. The increasing of the EtOH/H_2_O ratio than pure H_2_O solvent in the electrosynthesis procedure can lead to the lower crystallinity of structure and smaller particles^32–34^.

**Figure 12:**
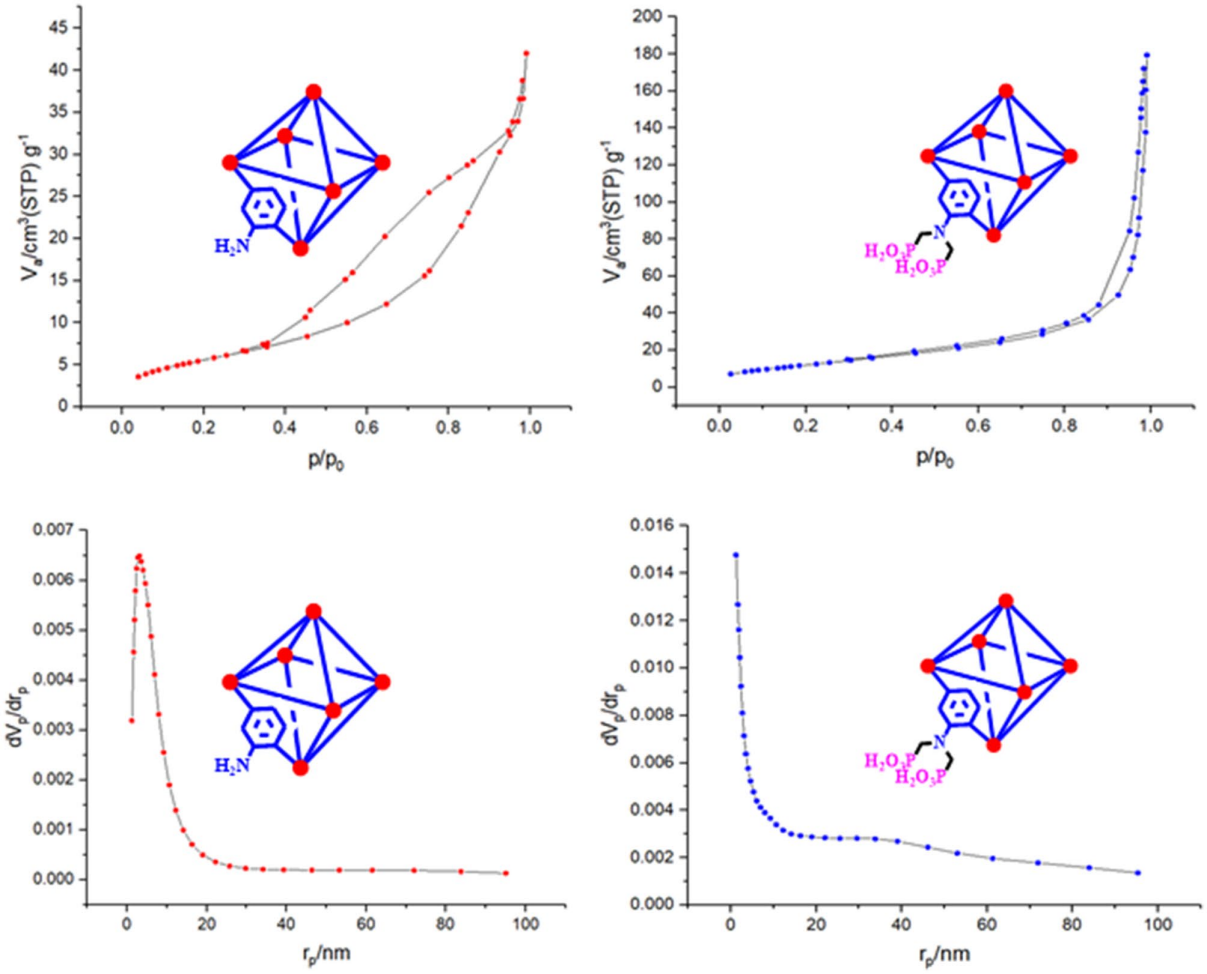
N_2_ adsorption/desorption isotherm and BJH of MIL-53(Al)-N(CH_2_PO_3_H_2_)_2_ and MIL-53(Al)-N(CH_2_PO_3_H_2_)_2_.

The specific surface area and pore size distribution are considered as significant parameters of the prepared porous materials and can be affected on the catalysis performance. In this way, the N_2_ adsorption/desorption technique was employed for the investigation of the MIL-53(Al)-NH_2_ and MIL-53(Al)-N(CH_2_PO_3_H_2_)_2_ structure porosity (Fig. 12). As can be seen, the N_2_ adsorption/desorption plots exhibit the IV-type isotherms with the hysteresis loops as a feature of the mesoporous materials. It is noteworthy, the created mesophase is consistent with the fact that increased EtOH/H_2_O ratio in the synthesis process can lead to mesoporosity structures^32–34^. According to the obtained BET results, the specific surface areas of MIL-53(Al)-NH_2_ and MIL-53(Al)-N(CH_2_PO_3_H_2_)_2_ structure are 204.75 and 132.19 m^2^ g^−1^, respectively. Also, according to the obtained BJH information, the pore size of MIL-53(Al)-NH_2_ and MIL-53(Al)-N(CH_2_PO_3_H_2_)_2_ structure was determined to be 4.2 nm and 3.2 nm, respectively. It should be noted that the surface area and pore size of functionalized MOF was decreased. Thus, it can be assigned to the presence of the larger phosphorus acid tags than amine groups during the post-modification process.

**Figure 13:**
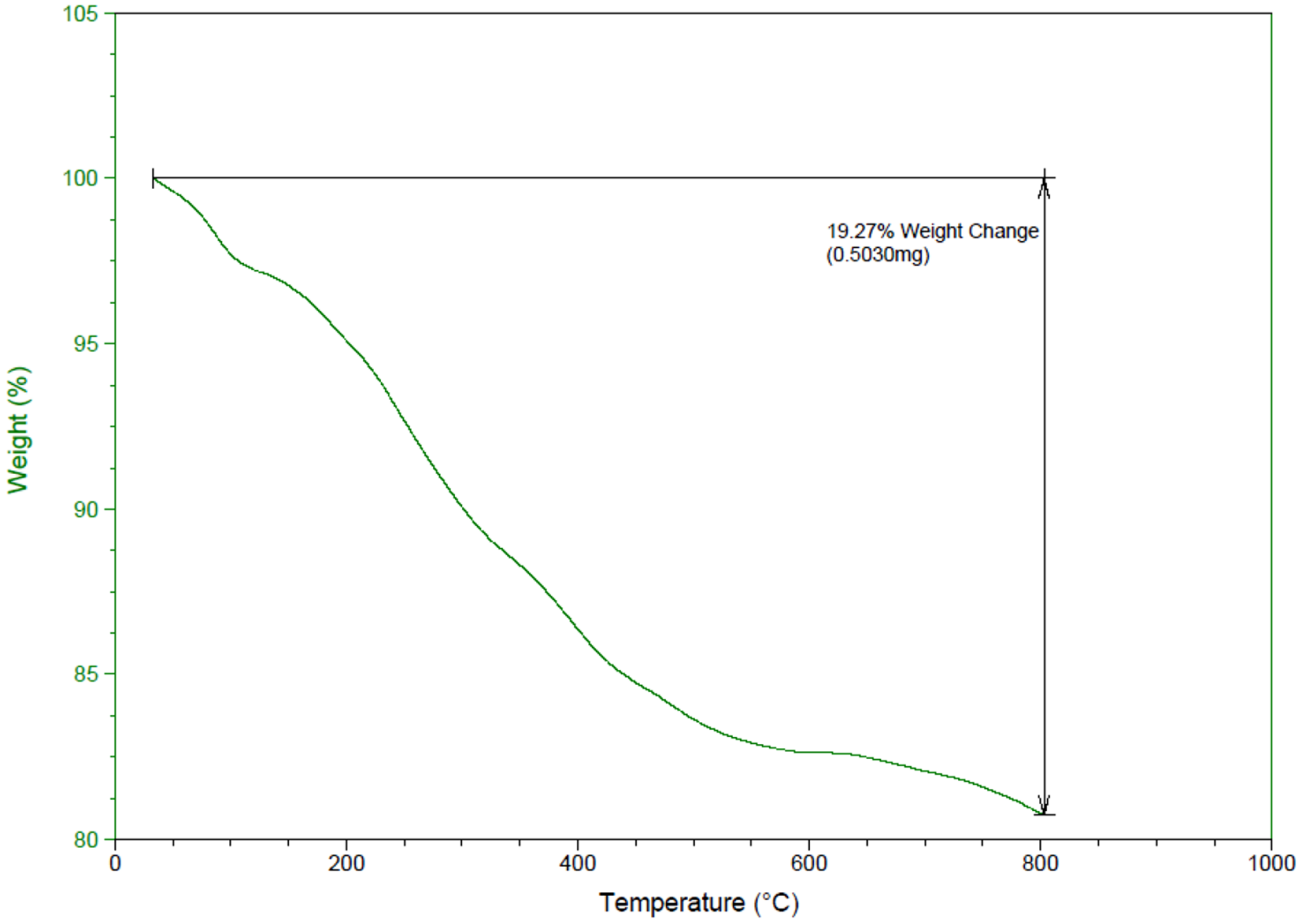
Thermogravimetric (TGA) analysis of MIL-53(Al)-N(CH_2_PO_3_H_2_)_2_.

The thermogravimetric (TGA) analysis for MIL-53(Al)-N(CH_2_PO_3_H_2_)_2_ was performed to study at range of 50–800 °C, with a temperature increase rate of 10 °C min^−1^ in CO_2_ atmosphere (Fig. 13). The first distinguished weight loss step in the temperature zone of 50–100 °C can be assigned to the evaporation and removal of solvents. The second distinguished weight loss step in the temperature zone of 150–450 °C can be assigned to the decomposition of the organic section of material includes the ligand and phosphorus acid which leads to the degradation and collapse of the framework.

After identification of MIL-53(Al)-N(CH_2_PO_3_H_2_)_2_ as mesoporous hybrid-catalyst, we tested its catalytic activity in the preparation of novel organic and biological interest candidates (*N*-methyl-pyrrol)-pyrazolo[3,4-*b*]pyridines derivatives. Optimization of reaction for synthesis of target molecules were done by one-pot reaction of benzaldehyde (1.0 mmol, 0.106 g), 3-methyl-1-phenyl-1*H*-pyrazol-5-amine (1.0 mmol, 0.174 g) and 3-(1-methyl-1*H*-pyrrol-2-yl)-3-oxopropanenitrile (1.0 mmol, 0.148 g) as a model reaction. The optimized data is listed in Table 1. As shown, the most optimal one-pot reaction for the preparation of (*N*-methyl-pyrrol)-pyrazolo[3,4- *b*]pyridines is reported in the presence of 10.0 mg MIL-53(Al)-N(CH_2_PO_3_H_2_)_2_ at 110 °C under solvent-free condition (Table 1 entry 4). The obtained data for the model reaction using other amounts of catalyst and temperature shows that the yield and time were not improved (Table 1 entries 1–8 except 4). The model reaction was also studied by using several solvents such as EtOH, CH_2_Cl_2_, CHCl_3_, EtOAc, CH_3_CN, PEG, *n*-Hexane, H_2_O and DMF (5.0 mL) and solvent-free condition in the presence of 10.0 mg of catalyst. The results of the reaction did not improve (Table 1, entries 9–17).

**Table 1:** Effect of different amounts of catalysts, temperature and solvent (5 mL) in the synthesis of (*N*-methyl-pyrrol)-pyrazolo[3,4-*b*]pyridines.


Entry	Amount of cat. (mg)	Temp. (°C)	Time (min)	Solvent	Yield^a^ (%)
1	–	110	60	–	Trace
2	5	110	60	–	35
3	7.5	110	60	–	55
4	10	110	25	–	88
5	15	110	25	–	88
6	10	75	60	–	65
7	10	50	60	–	40
8	10	r.t	60	–	Trace
9	10	Reflux	60	EtOH	52
10	10	Reflux	60	CH_2_Cl_2_	Trace
11	10	Reflux	60	CHCl_3_	45
12	10	Reflux	60	EtOAc	48
13	10	Reflux	60	CH_3_CN	55
14	10	110	60	PEG	65
15	10	Reflux	60	*n*-Hexane	Trace
16	10	Reflux	60	H_2_O	45
17	10	110	60	DMF	55

^a^Reaction conditions: benzaldehyde (1.0 mmol, 0.106 g), 3-methyl-1-phenyl-1*H*-pyrazol-5-amine (1.0 mmol, 0.174 g) and 3-(1-methyl-1*H*-pyrrol-2-yl)-3-oxopropanenitrile (1.0 mmol, 0.148 g).

After optimizing the reaction conditions, MIL-53(Al)-N(CH_2_PO_3_H_2_)_2_ (10.0 mg) is applied to synthesis a good range of new biological and pharmacological interest candidates using various aromatic aldehyde derivatives (trephetaldehyde, iso-trephetaldehyde, tripodal-aldehyde, heterocycle, bearing electron-donating and electron-withdrawing groups), 3-methyl-1-phenyl-1*H*-pyrazol-5-amine and 3-(1-methyl-1*H*-pyrrol-2-yl)-3-oxopropanenitrile. As shown in Tables 2 and 3, the obtained results indicated that MIL-53(Al)-N(CH_2_PO_3_H_2_)_2_ is appropriate for the preparation of target molecules in high to excellent yields (65–92%) within relatively short reaction times (20–40 min). Interestingly, when we synthesized (*N*-methyl-pyrrol)-pyrazolo[3,4-*b*]pyridines in the percent indole aldehyde as a biological and pharmacological compound (Table 2). Also, the preparation of bis and tris-(*N*-methyl-pyrrol)-pyrazolo[3,4-*b*]pyridines by the condensation of 3-methyl-1-phenyl-1*H*-pyrazol-5-amine and 3-(1-methyl-1*H*-pyrrol-2-yl)-3-oxopropanenitrile with terephthaldehyde, *iso*-terephthaldehyde and tripodal aldehyde using MIL-53(Al)-N(CH_2_PO_3_H_2_)_2_ (10.0 mg) at 110 °C under solvent-free condition (Table 3).

**Table 2:**
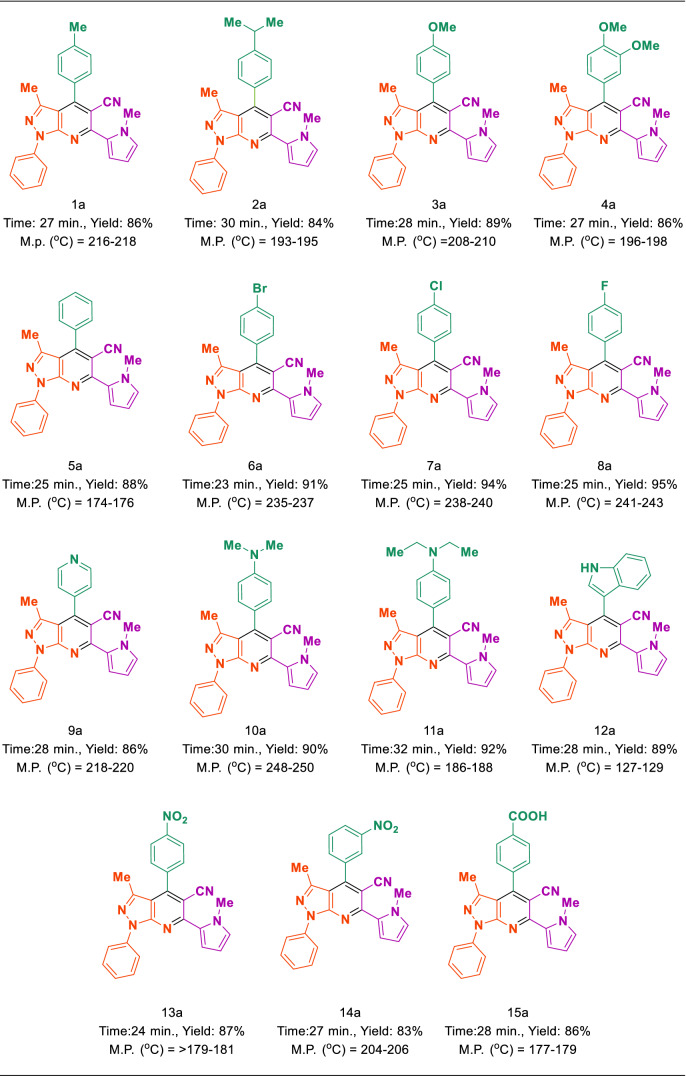
Synthesis of (*N*-methyl-pyrrol)-pyrazolo[3,4-*b*]pyridines using MIL-53(Al)-N(CH_2_PO_3_H_2_)_2_ (10 mg) at 110 °C under solvent-free condition.

**Table 3:**
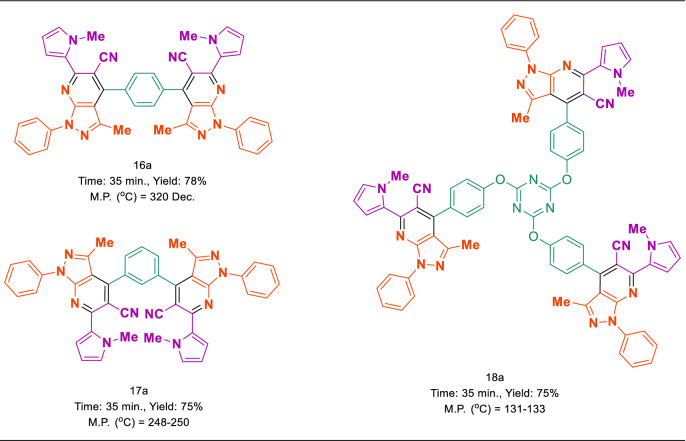
Synthesis of (*N*-methyl-pyrrol)-pyrazolo[3,4-*b*]pyridines using MIL-53(Al)-N(CH_2_PO_3_H_2_)_2_ (10 mg) at 110 °C under solvent-free condition.

**Figure 14:**
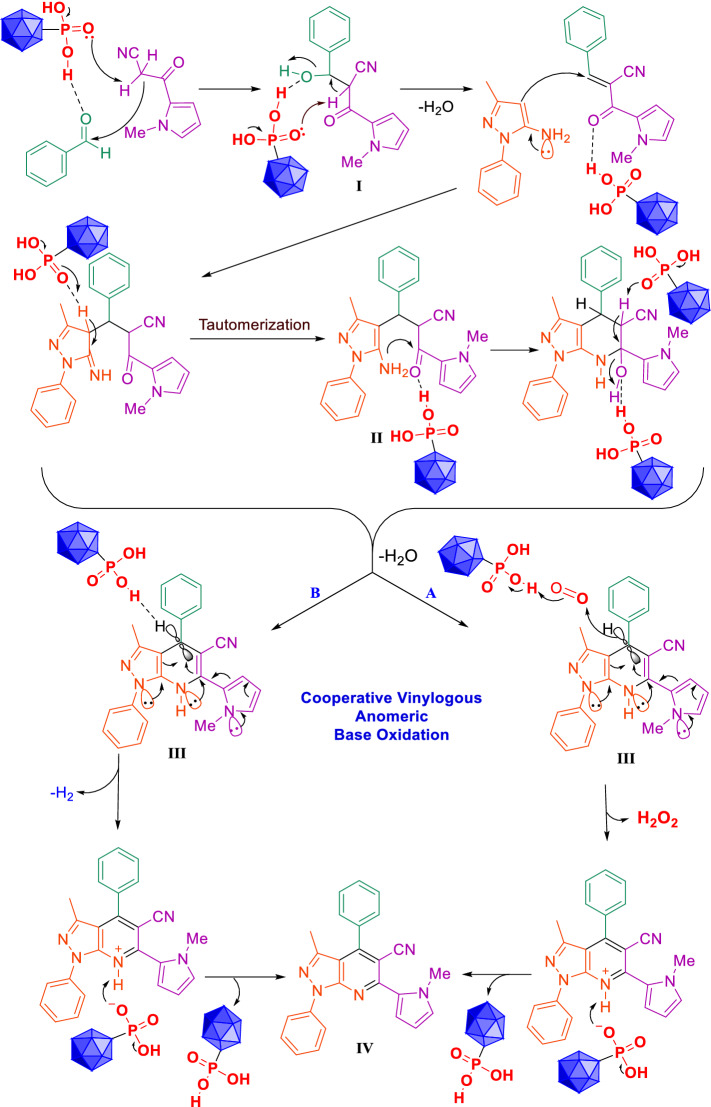
Plausible mechanisms for the synthesis (*N*-methyl-pyrrol)-pyrazolo[3,4-*b*]pyridines using MIL-53(Al)-N(CH_2_PO_3_H_2_)_2_.

In suggested mechanism, aldehyde is activated with PO_3_H_2_ tags of MIL-53(Al)-N(CH_2_PO_3_H_2_)_2_ and intermediate (I) is prepared by reaction of 3-(1-methyl-1*H*-pyrrol-2-yl)-3-oxopropanenitrile with the loss of H_2_O. In the second step, phenyl pyrazol-5-amine react with intermediate (I) to gives intermediate (II) after tautomerization. Then, intermediate (II) gives intermediates (III) after intramolecular cyclization and loss of one molecule of H_2_O. Then, the corresponding product (IV) is produced via a cooperative vinylogous anomeric based oxidation mechanism both in the presence and absence of oxygen (pathways A and B in Fig. 14)^62,63,67,68,79^. It should be mentioned that the reaction in the absence of oxygen and under both nitrogen and argon atmospheres have proceeded as same as in the presence of oxygen. Recently, we have comprehensively showed that the hetero atoms have stereoelectronic effects of common origin and that anomeric effect is one of them^80^.

To evaluate the performance of MIL-53(Al)-N(CH_2_PO_3_H_2_)_2_ as a catalyst for the synthesis of (*N*-methyl-pyrrol)-pyrazolo[3,4-*b*]pyridines, we have tested various acid catalysts (organic and inorganic) in the reaction of benzaldehyde (1.0 mmol, 0.106 g), 3-methyl-1-phenyl-1*H*-pyrazol-5-amine (1.0 mmol, 0.174 g) and 3-(1-methyl-1*H*-pyrrol-2-yl)-3-oxopropanenitrile (1.0 mmol, 0.148 g) in Table 4. The obtained results which are collected in Table 4 shows that, MIL-53(Al)-N(CH_2_PO_3_H_2_)_2_ is the best catalyst for the synthesis of a novel (*N*-methyl-pyrrol)-pyrazolo[3,4-*b*]pyridines.

**Table 4:** Evaluation of various catalyst for the synthesis of novel (*N*-methyl-pyrrol)-pyrazolo[3,4-*b*]pyridines in comparison with MIL-53(Al)-N(CH_2_PO_3_H_2_)_2_.


Entry	Catalyst	(mg)	Time (min)	Yield (%)
1	[PVI-SO_3_H]FeCl_4_^81^	10	60	55
2	NaHSO_4_	10 mol%	60	28
3	Fe_3_O_4_	10	60	35
4	SSA^82^	10	60	45
5	[Fe_3_O_4_@SiO_2_@(CH_2_)_3_-DABCO-SO_3_H]Cl_2_^83^	10	60	35
6	FeCl_3_	10 mol%	60	45
7	TrBr^84^	10 mol%	60	Trace
8	GTBSA^85^	10 mol%	60	65
9	Mg(NO_3_)_2_·6H_2_O	10 mol%	60	Trace
10	[Py-SO_3_H]Cl^86^	10 mol%	60	65
11	NH_4_NO_3_	10 mol%	60	–
12	MIL-100(Cr)/NHEtN(CH_2_PO_3_H_2_)_2_^47^	10	60	75
13	APVPB^87^	10	60	45
14	MHMHPA^50^	10 mol%	60	55
15	MIL-53(Al)-N(CH_2_PO_3_H_2_)_2_^This work^	10	25	88

**Figure 15:**
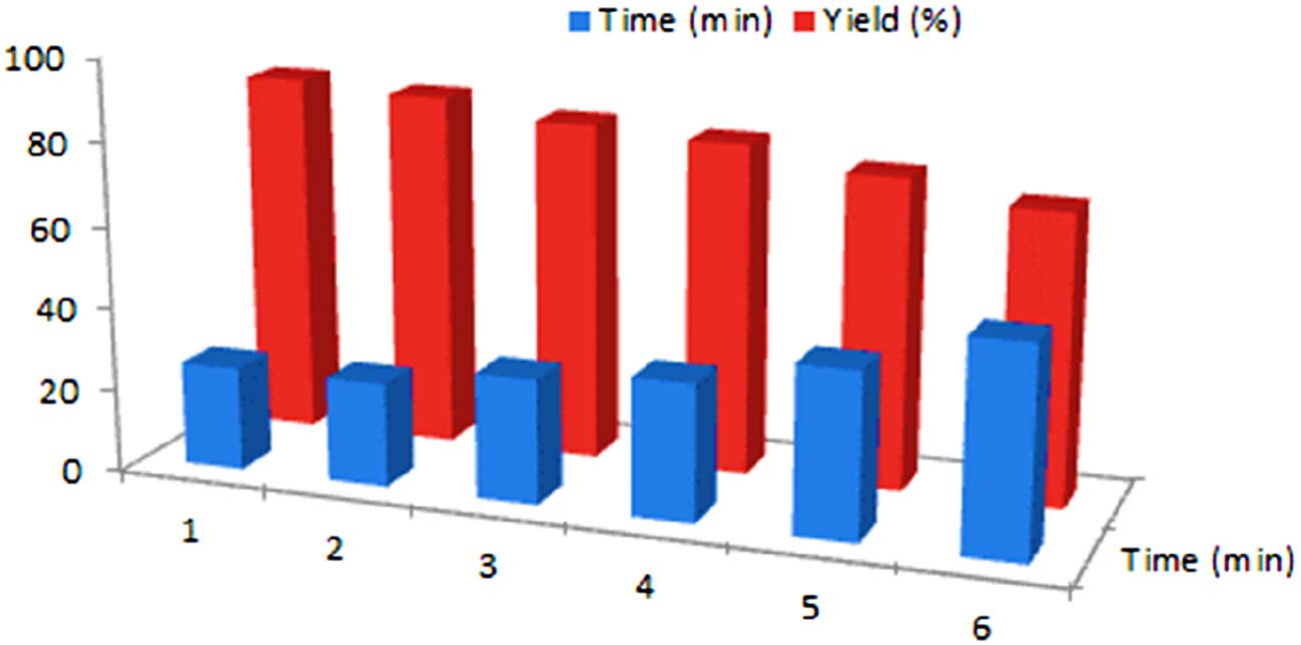
Reusability of MIL-53(Al)-N(CH_2_PO_3_H_2_)_2_.

The obtained results of catalytic activity and reusability of MIL-53(Al)-N(CH_2_PO_3_H_2_)_2_ were added in (Fig. 15). As above said, MIL-53(Al)-N(CH_2_PO_3_H_2_)_2_ was separated by centrifugation and reused without significantly reducing its catalytic activity. For this purpose, recyclability of the catalyst was also studied on the one-pot reaction of benzaldehyde (1.0 mmol, 0.106 g), 3-methyl-1-phenyl-1*H*-pyrazol-5-amine (1.0 mmol, 0.174 g) and 3-(1-methyl-1*H*-pyrrol-2-yl)-3-oxopropanenitrile (1.0 mmol, 0.148 g) as a model reaction under the above-mentioned optimized reaction conditions. Therefore, MIL-53(Al)-N(CH_2_PO_3_H_2_)_2_ can be reused up to five times without noticeable changes in its catalytic activity.

The large scale synthesis of (*N*-methyl-pyrrol)-pyrazolo[3,4-*b*]pyridines by the reaction of aldehyde (20 mmol), 3-methyl-1-phenyl-1*H*-pyrazol-5-amine (20 mmol, 3.48 g) and 3-(1-methyl-1*H*-pyrrol-2-yl)-3-oxopropanenitrile (20 mmol, 2.96 g) using MIL-53(Al)-N(CH_2_PO_3_H_2_)_2_ (1 g) at 110 °C (Table 5).

**Table 5:** Large scale synthesis of (*N*-methyl-pyrrol)-pyrazolo[3,4-*b*]pyridines.

Entry	Product	Time (min)	Yield (%)	M.p. °C
1		55	75	216–218
2		60	65	209–211

## Conclusion

In this study, electrosynthesis of MIL-53(Al)-NH_2_ as a metal–organic framework was presented. The anodic electrosynthesis as an environmentally friendly technique procedure was performed in the aqueous solution, room temperature, and atmospheric pressure in the shortest possible time. Also, the employed electrochemical technique provided a promising procedure for the preparation of mesoporous catalyst with a shorter time and high yield. Then its functionalization with the phosphorus acid tags through the post-modification process has occurred. It is noteworthy, this procedure was accomplished without the need for the ex-situ salt and base/pre-base additives as cation source and ligand activating agent, respectively. MIL-53(Al)-N(CH_2_PO_3_H_2_)_2_ as an efficient catalyst was used for the synthesis of the novel (*N*-methyl-pyrrol)-pyrazolo[3,4-*b*]pyridines as biological interest molecules via a cooperative vinylogous anomeric based oxidation mechanism. Short reaction time, clean profile of reaction, recycling and reusing of catalyst are the major advantages of the presented work. We think that the present work can open up a promising insight for developing of anomeric effect in the course of organic synthesis.

## Supplementary Information

Supplementary Information.
