# Supplementary material for: Anodic electrosynthesis of MIL-53(Al)-N(CH2PO3H2)2 as a mesoporous catalyst for synthesis of novel (N-methyl-pyrrol)-pyrazolo[3,4-b]pyridines via a cooperative vinylogous anomeric based oxidation
Source: Sci Rep. 2021 Sep 29;11:19370. doi: 10.1038/s41598-021-97801-7 (PMC8481481; doi:10.1038/s41598-021-97801-7)
Supplement: Supplementary file 1 — Supplementary Information. [file 41598_2021_97801_MOESM1_ESM.docx]

**Anodic Electrosynthesis of MIL-53(Al)-N(CH_2_PO_3_H_2_)_2_ as a mesoporous catalyst for synthesis of novel (*N*-methyl-pyrrol)-pyrazolo[3,4-*b*]pyridines *via* a cooperative vinylogous anomeric based oxidation**

*Sima Kalhor,^a^ Mahmoud Zarei,*^a^ Mohammad Ali Zolfigol,*^a^ Hassan Sepehrmansourie,^a^ Davood Nematollahi,*^b^ Saber Alizadeh,*^b^ Hu Shi*^c^ and Jalal Arjomandi^d^*

*^a^* Department of Organic Chemistry, Faculty of Chemistry, Bu-Ali Sina University, PO Box 6517838683, Hamedan, Iran. Tel: +988138282807, Fax: +988138380709 Iran. E-Mail: mahmoud8103@yahoo.com (M. Zarei) or zolfi@basu.ac.ir & mzolfigol@yahoo.com (M. A. Zolfigol).

*^b^* Department of Analytical Chemistry, Faculty of Chemistry, Bu-Ali Sina University, PO Box 6517838683, Hamedan, Iran. Tel: +988138282807, Fax: +988138380709 Iran. E-Mail: s.alizade66@yahoo.com, nemat@basu.ac.ir, [nematollahid@gmail.com](mailto:nematollahid@gmail.com†).

*^c^* School of Chemistry and Chemical Engineering, Institute of Molecular Science, Shanxi University, Taiyuan 030006, China. E-Mail: [hshi@sxu.edu.cn](mailto:hshi@sxu.edu.cn).

*^d^* Department of physical Chemistry, Faculty of Chemistry, Bu-Ali Sina University, PO Box 6517838683, Hamedan, Iran. Tel: +988138282807, Fax: +988138380709 Iran. E-Mail: [j_arjomandi@yahoo.com](mailto:j_arjomandi@yahoo.com)


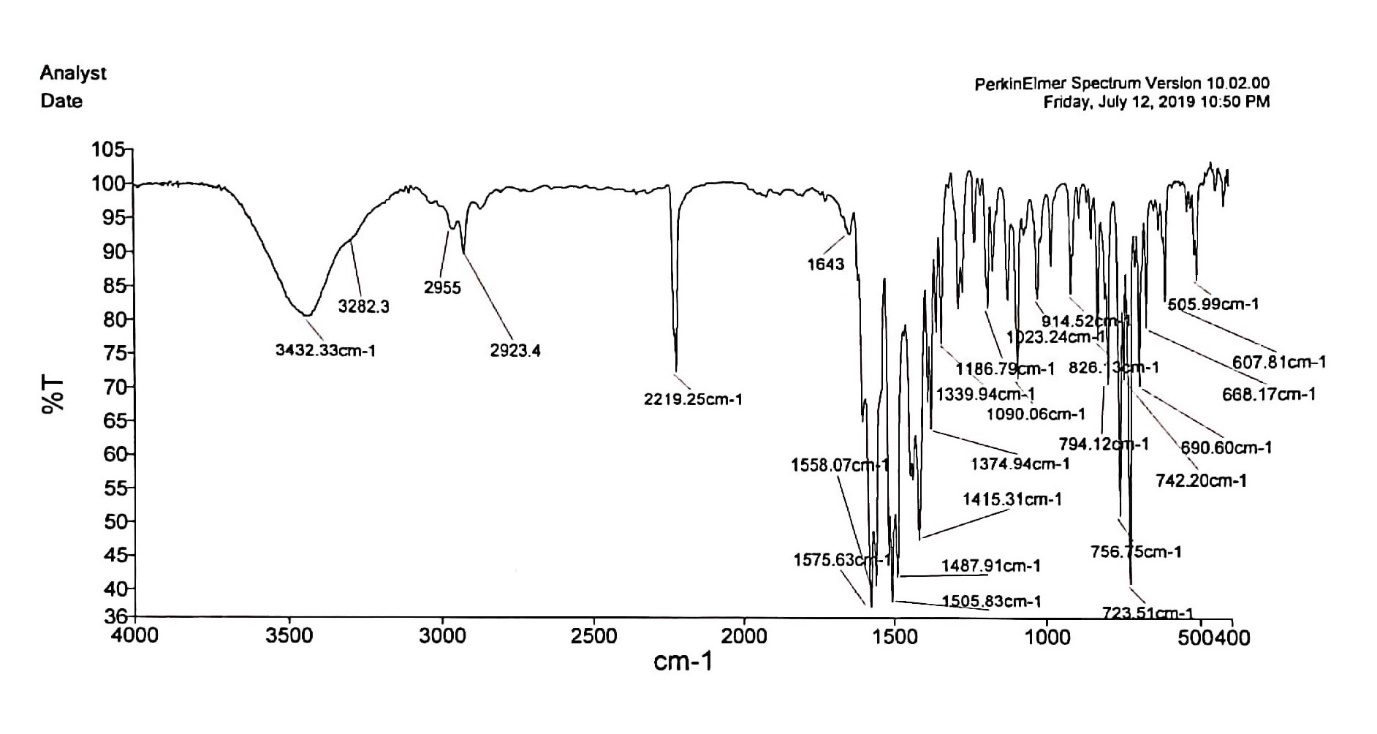


**Figure S1:** FT-IR spectrum of 3-methyl-6-(1-methyl-1*H*-pyrrol-2-yl)-1-phenyl-4-(*p*-tolyl)-1*H*-pyrazolo[3,4-*b*] pyridine-5-carbonitrile (1a)


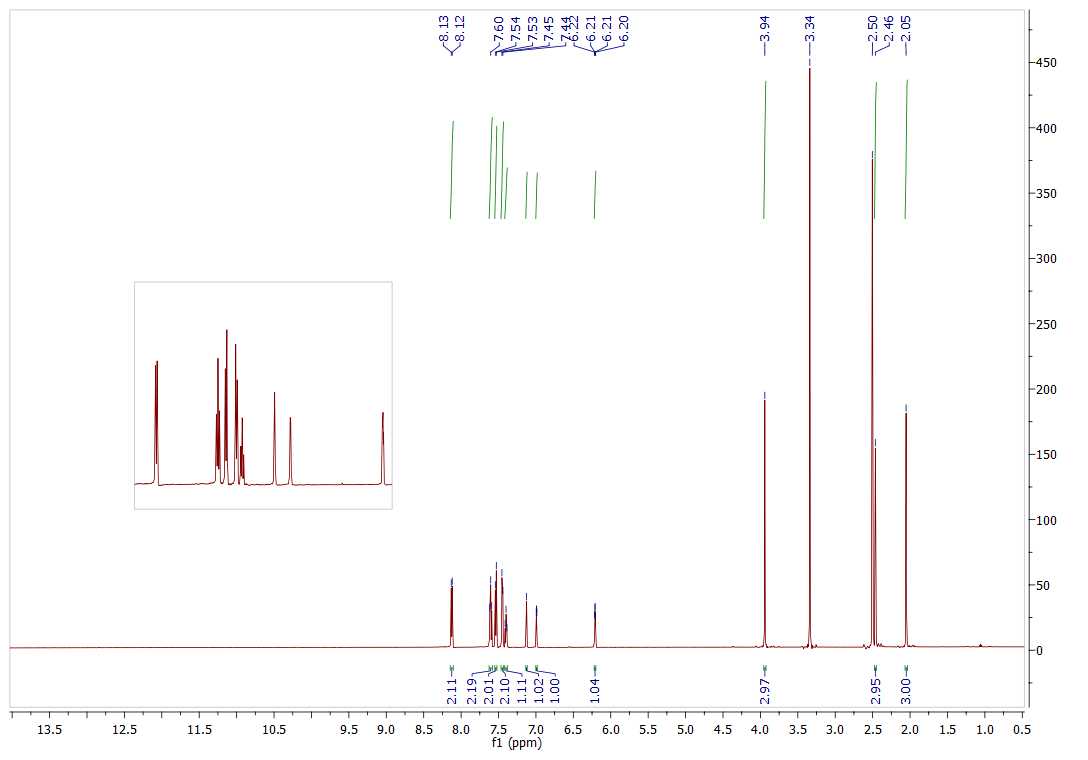

**Figure S2:** ^1^H NMR spectrum of 3-methyl-6-(1-methyl-1*H*-pyrrol-2-yl)-1-phenyl-4-(*p*-tolyl)-1*H*-pyrazolo[3,4-*b*] pyridine-5-carbonitrile (1a)


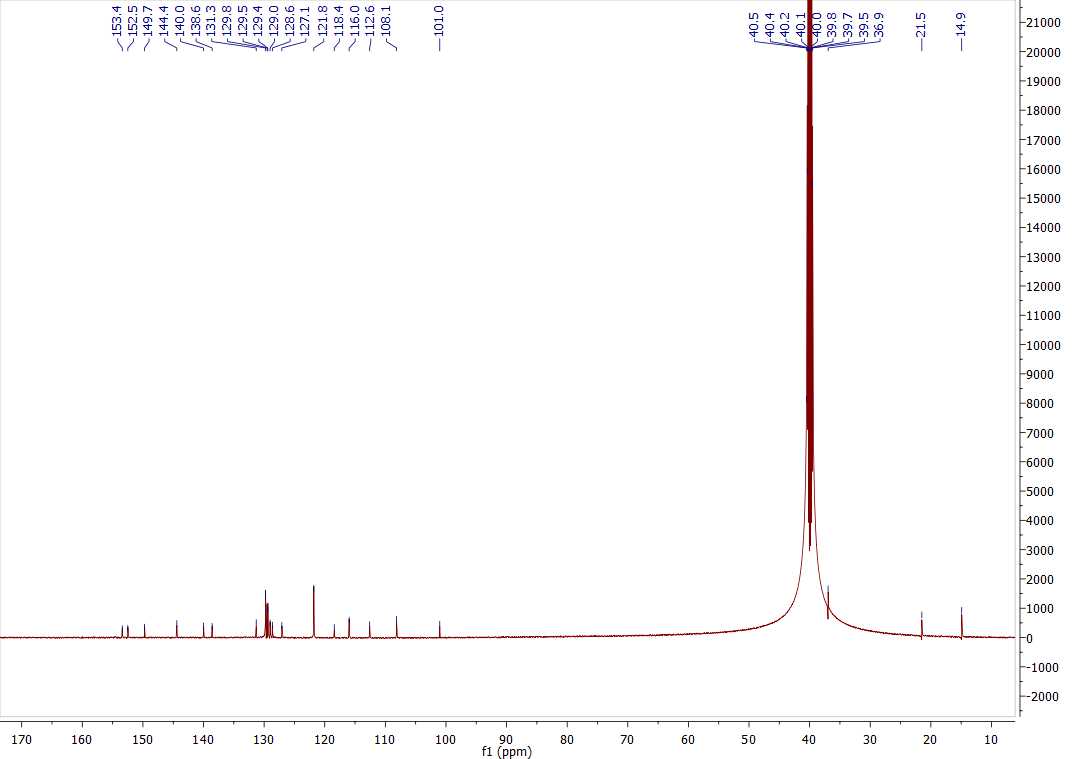

**Figure S3:** ^13^C NMR spectrum of 3-methyl-6-(1-methyl-1*H*-pyrrol-2-yl)-1-phenyl-4-(*p*-tolyl)-1*H*-pyrazolo[3,4-*b*] pyridine-5-carbonitrile (1a)


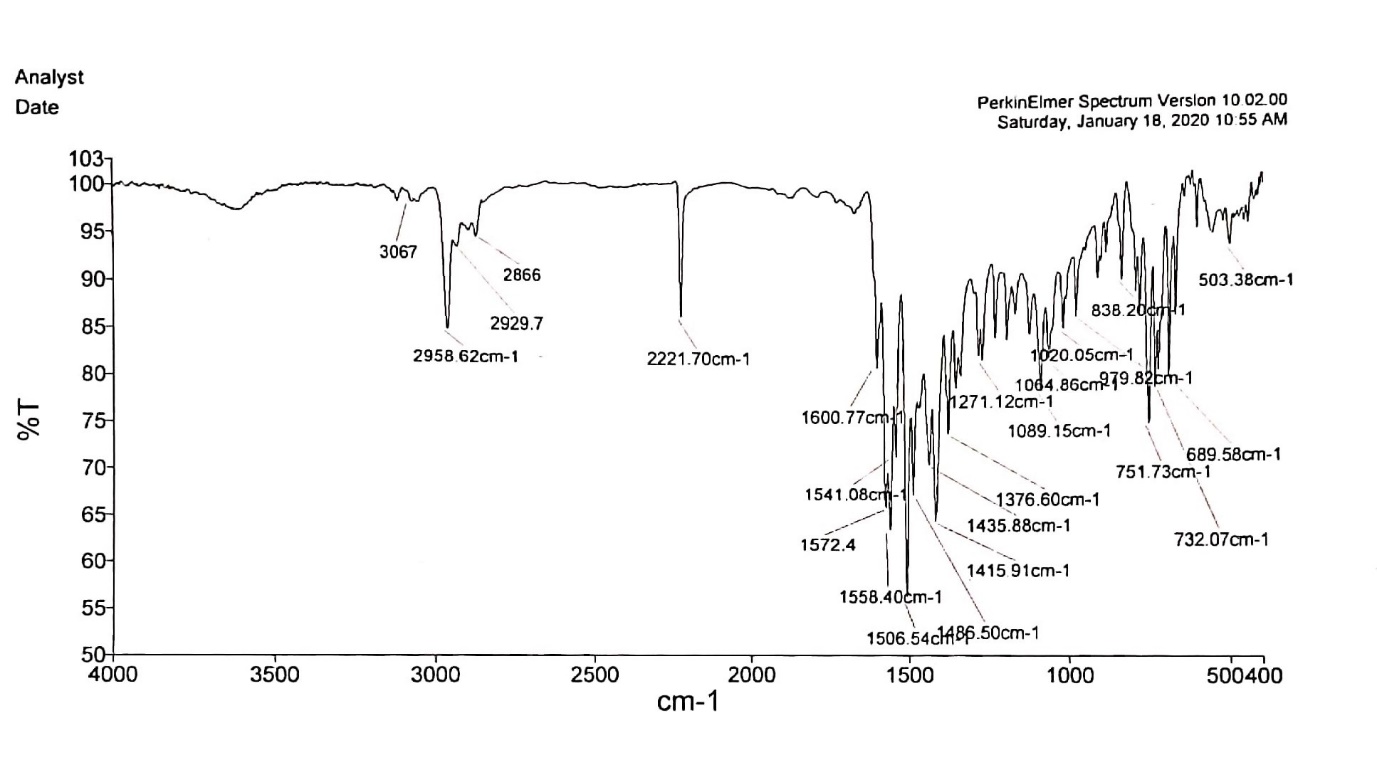

**Figure S4:** FT-IR spectrum of 4-(4-isopropylphenyl)-3-methyl-6-(1-methyl-1*H*-pyrrol-2-yl)-1-phenyl-1*H*-pyrazolo[3,4-*b*] pyridine-5-carbonitrile (2a)


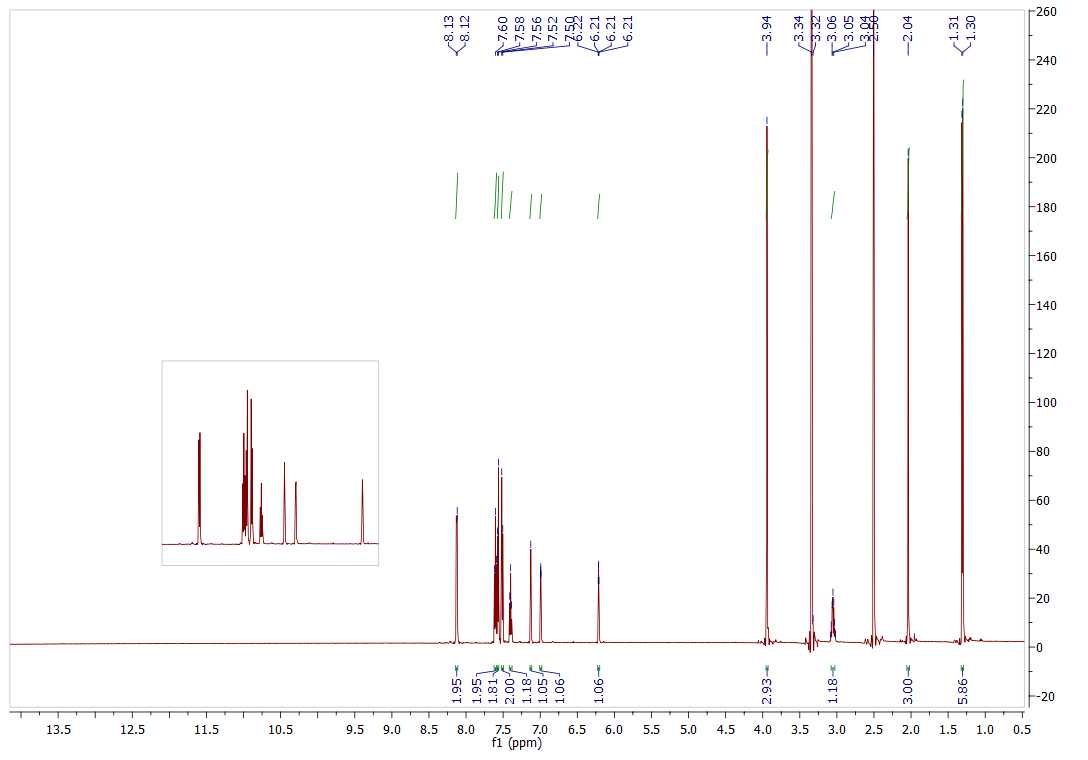

**Figure S5:** ^1^H NMR spectrum of 4-(4-isopropylphenyl)-3-methyl-6-(1-methyl-1*H*-pyrrol-2-yl)-1-phenyl-1*H*-pyrazolo[3,4-*b*] pyridine-5-carbonitrile (2a)


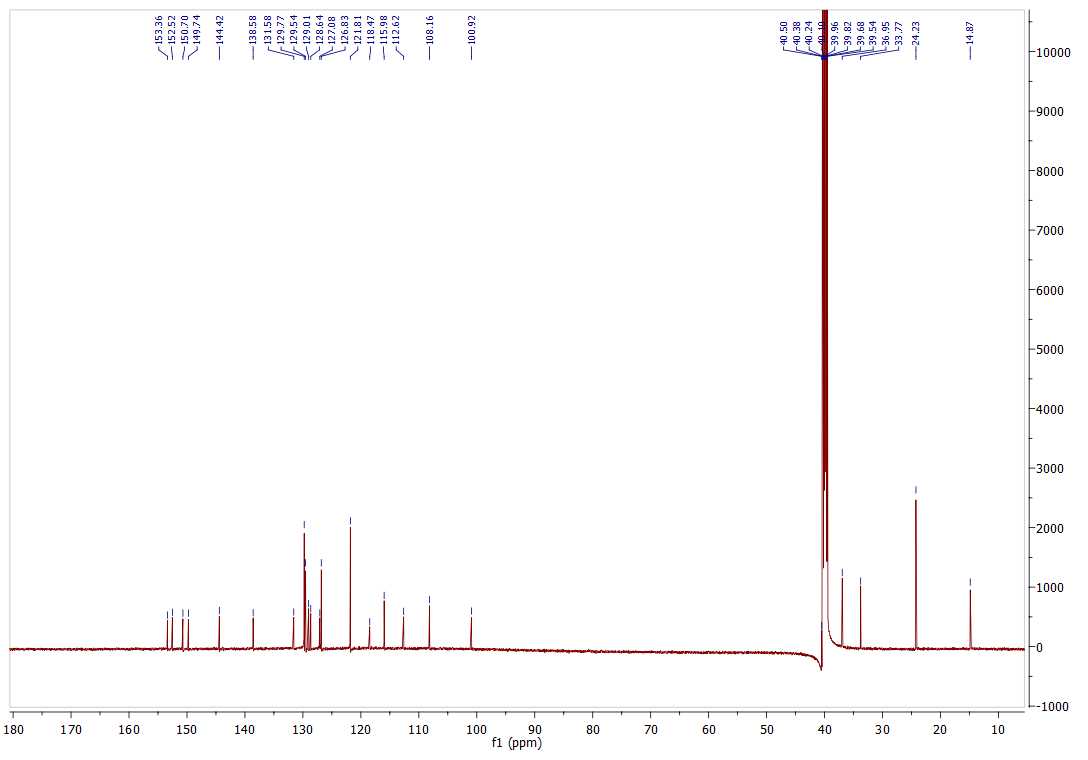

**Figure S6:** ^13^C NMR spectrum of 4-(4-isopropylphenyl)-3-methyl-6-(1-methyl-1*H*-pyrrol-2-yl)-1-phenyl-1*H*-pyrazolo[3,4-*b*] pyridine-5-carbonitrile (2a)


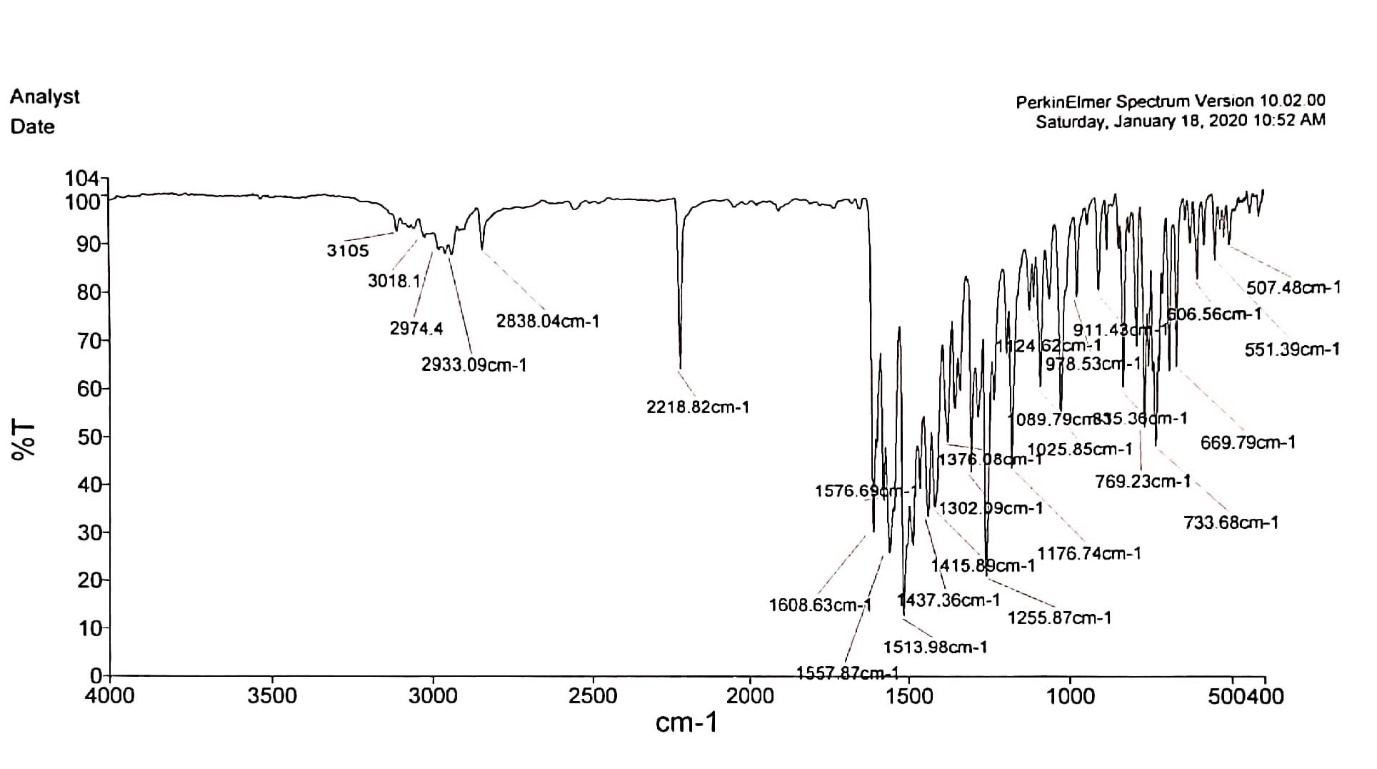

**Figure S7:** FT-IR spectrum of 4-(4-methoxyphenyl)-3-methyl-6-(1-methyl-1*H*-pyrrol-2-yl)-1-phenyl-1*H*-pyrazolo[3,4-*b*] pyridine-5-carbonitrile (3a)


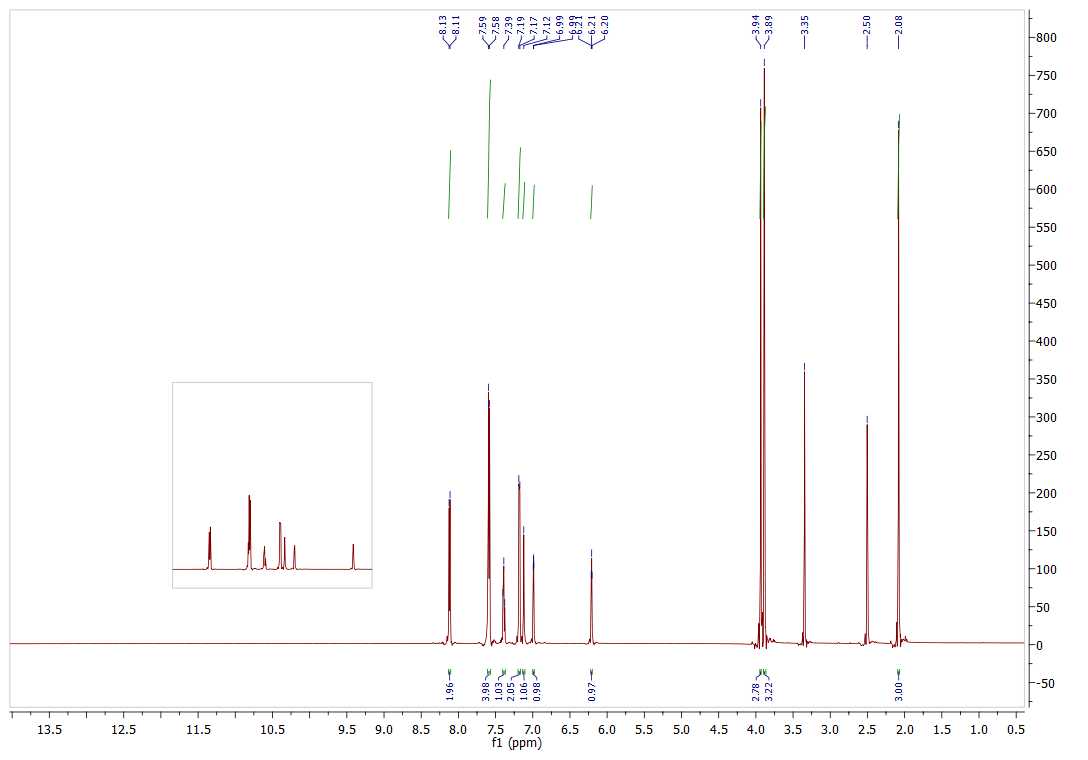

**Figure S8:** ^1^H NMR spectrum of 4-(4-methoxyphenyl)-3-methyl-6-(1-methyl-1*H*-pyrrol-2-yl)-1-phenyl-1*H*-pyrazolo[3,4-*b*] pyridine-5-carbonitrile (3a)


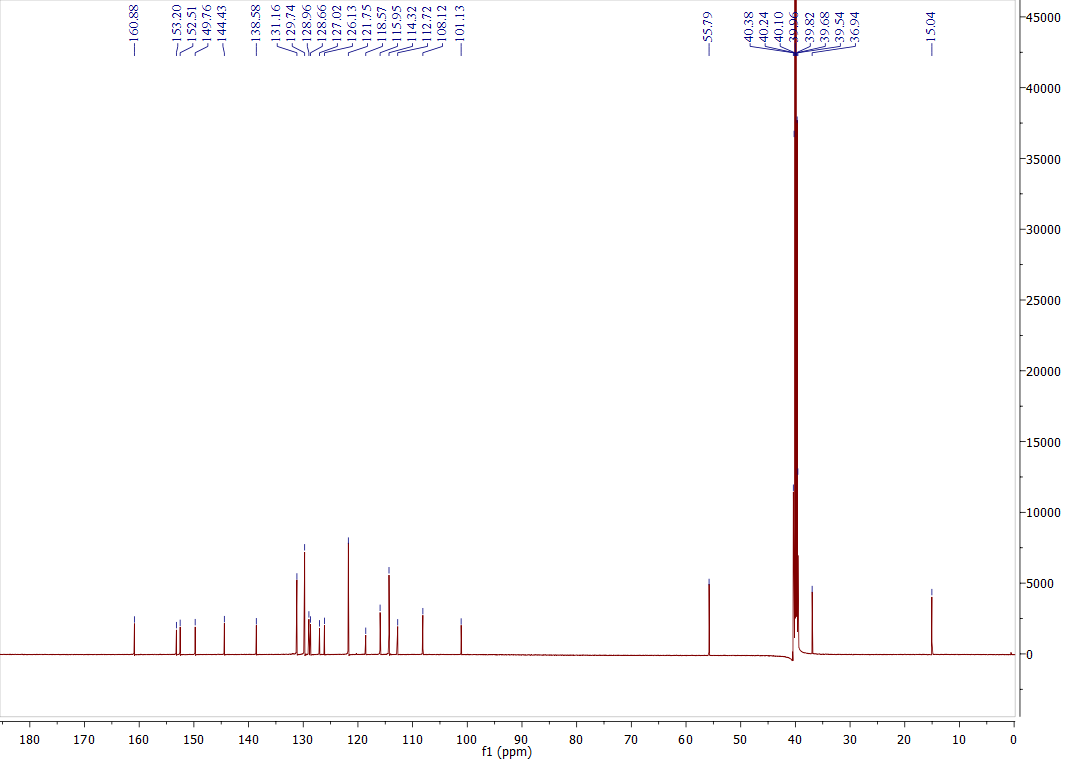

**Figure S9:** ^13^C NMR spectrum of 4-(4-methoxyphenyl)-3-methyl-6-(1-methyl-1*H*-pyrrol-2-yl)-1-phenyl-1*H*-pyrazolo[3,4-*b*] pyridine-5-carbonitrile (3a)


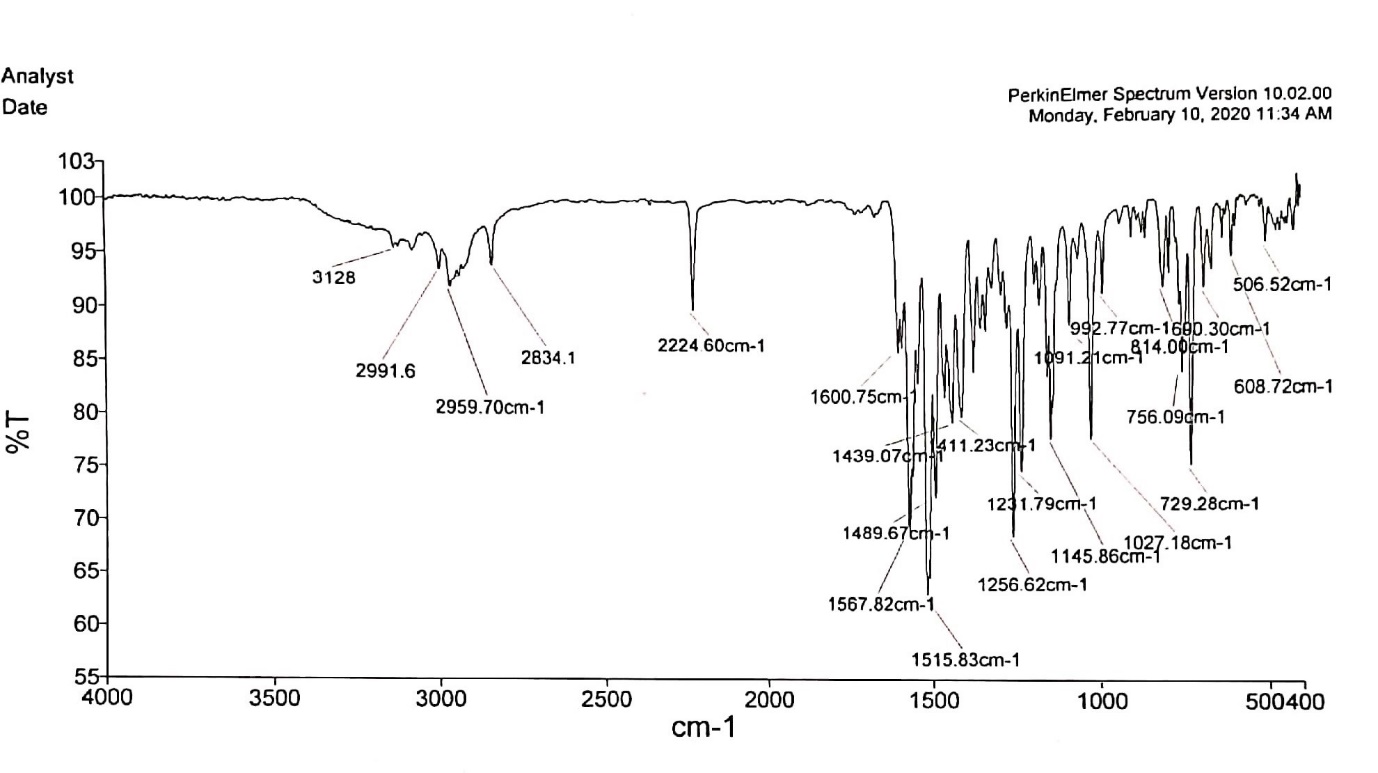

**Figure S10:** FT-IR spectrum of 4-(3,4-dimethoxyphenyl)-3-methyl-6-(1-methyl-1*H*-pyrrol-2-yl)-1-phenyl-1*H*-pyrazolo[3,4-*b*] pyridine-5-carbonitrile (4a)


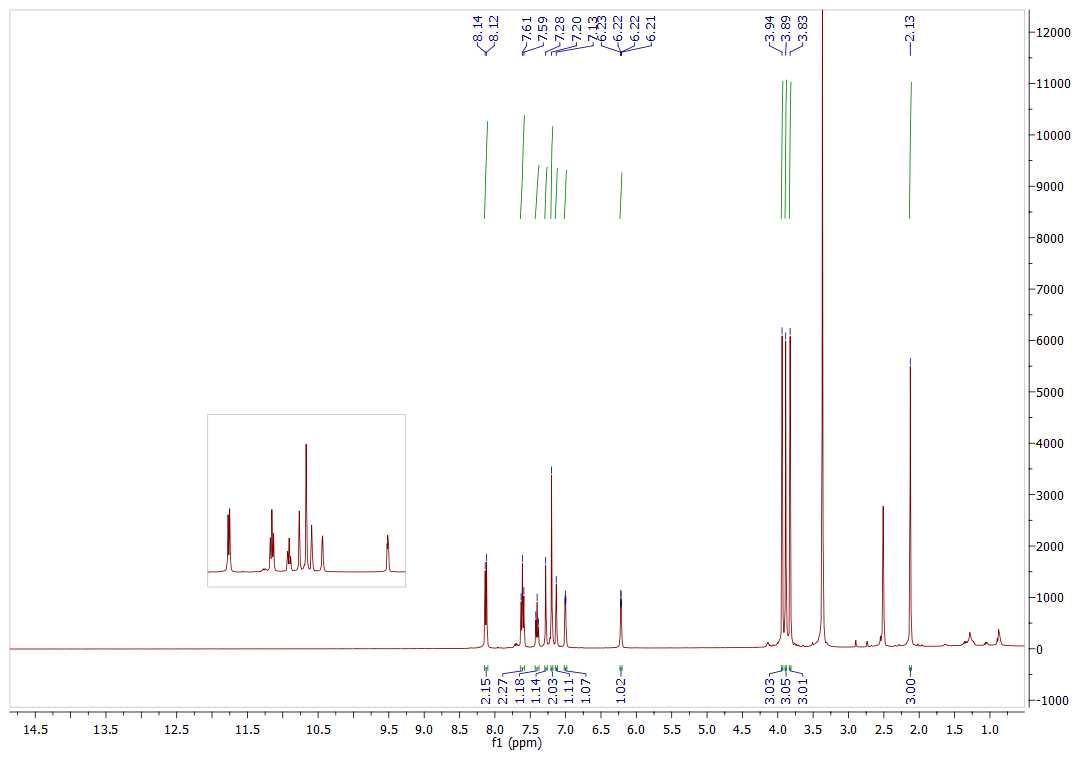

**Figure S11:** ^1^H NMR spectrum of 4-(3,4-dimethoxyphenyl)-3-methyl-6-(1-methyl-1*H*-pyrrol-2-yl)-1-phenyl-1*H*-pyrazolo[3,4-*b*] pyridine-5-carbonitrile (4a)


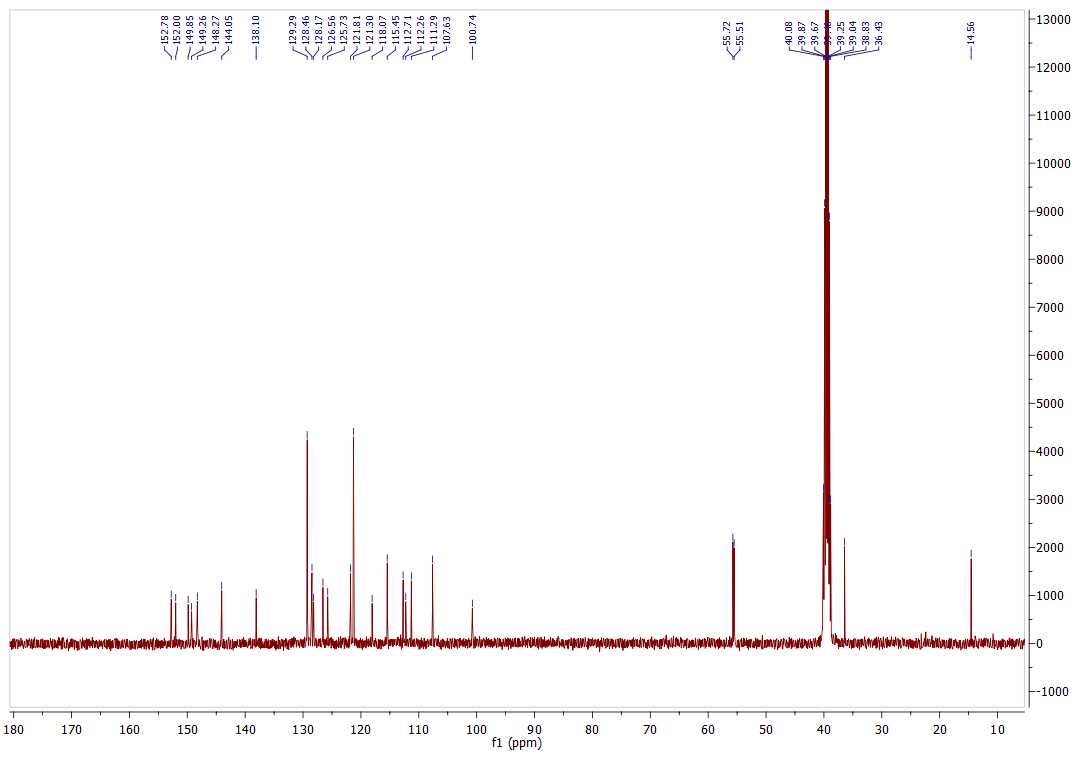

**Figure S12:** ^13^C NMR spectrum of 4-(3,4-dimethoxyphenyl)-3-methyl-6-(1-methyl-1*H*-pyrrol-2-yl)-1-phenyl-1*H*-pyrazolo[3,4-*b*] pyridine-5-carbonitrile (4a)


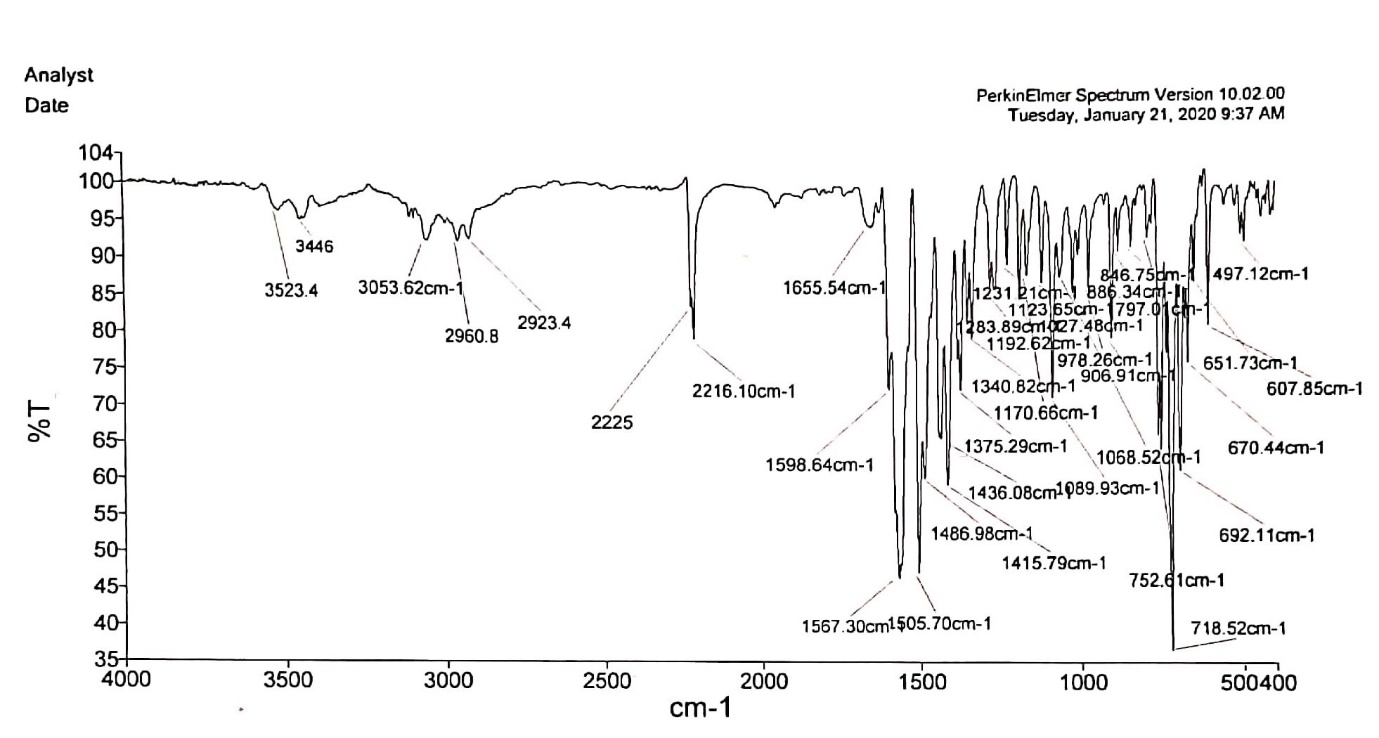

**Figure S13:** FT-IR spectrum of 3-methyl-6-(1-methyl-1*H*-pyrrol-2-yl)-1,4-diphenyl-1*H*-pyrazolo[3,4-*b*]pyridine-5 carbonitrile (5a)


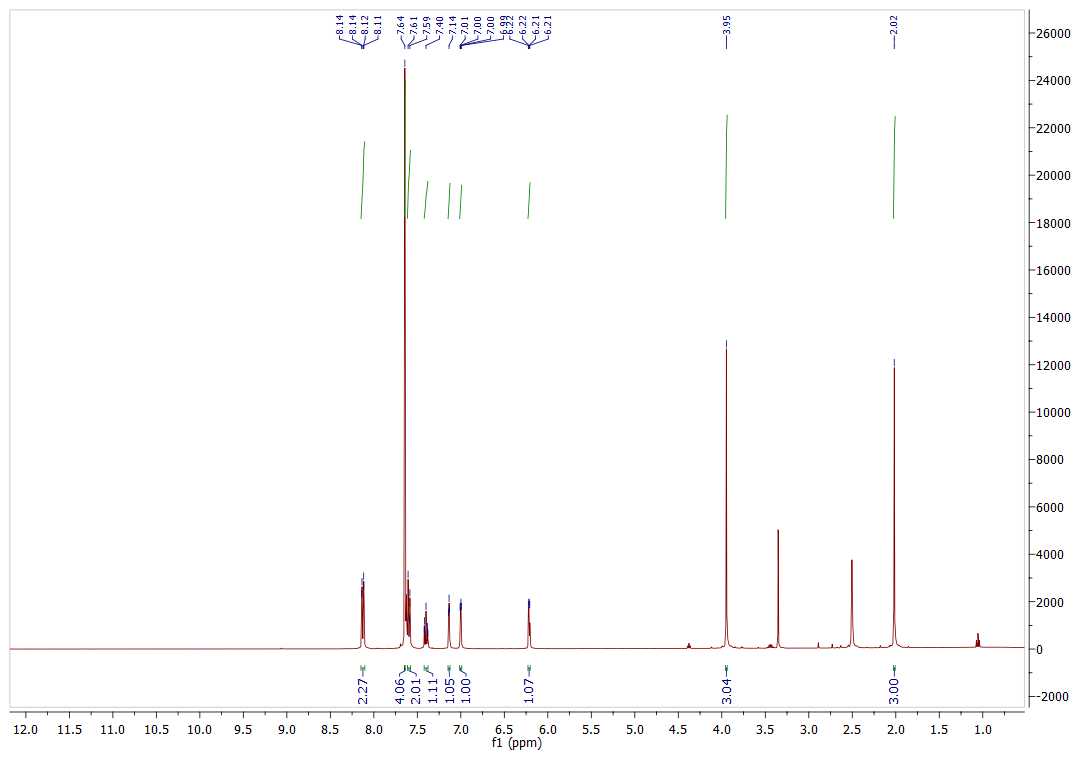

**Figure S14:** ^1^H NMR spectrum of 3-methyl-6-(1-methyl-1*H*-pyrrol-2-yl)-1,4-diphenyl-1*H*-pyrazolo[3,4-*b*]pyridine-5 carbonitrile (5a)


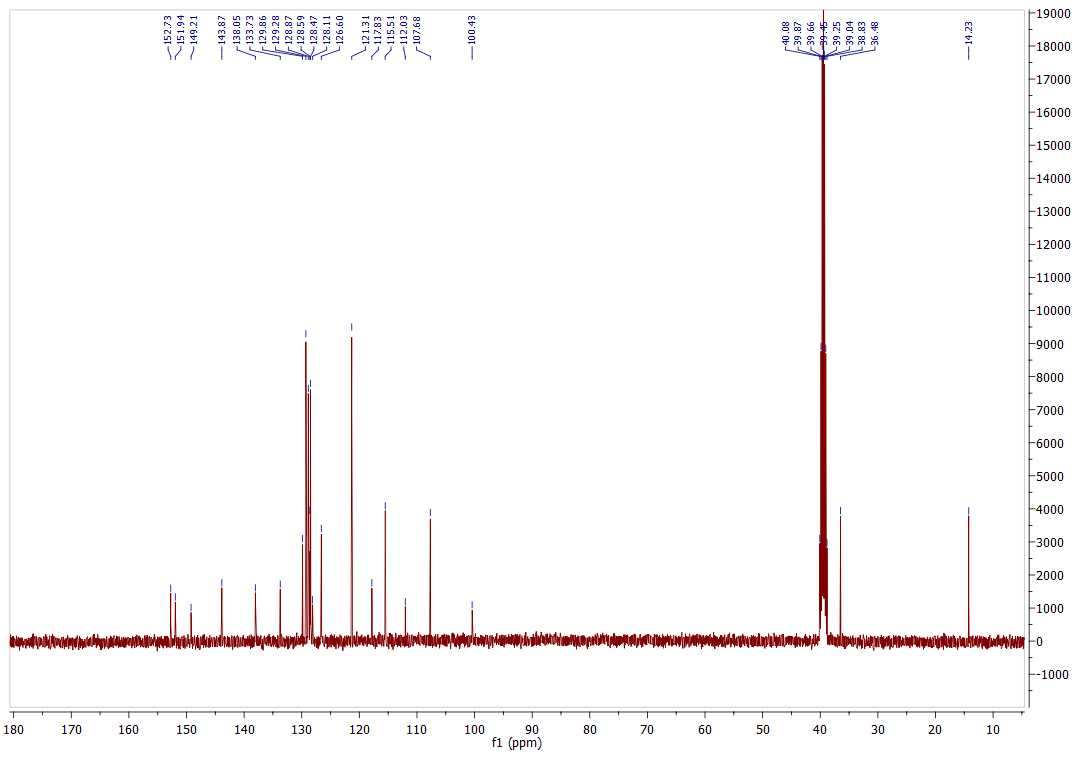

**Figure S15:** ^13^C NMR spectrum of 3-methyl-6-(1-methyl-1*H*-pyrrol-2-yl)-1,4-diphenyl-1*H*-pyrazolo[3,4-*b*]pyridine-5 carbonitrile (5a)


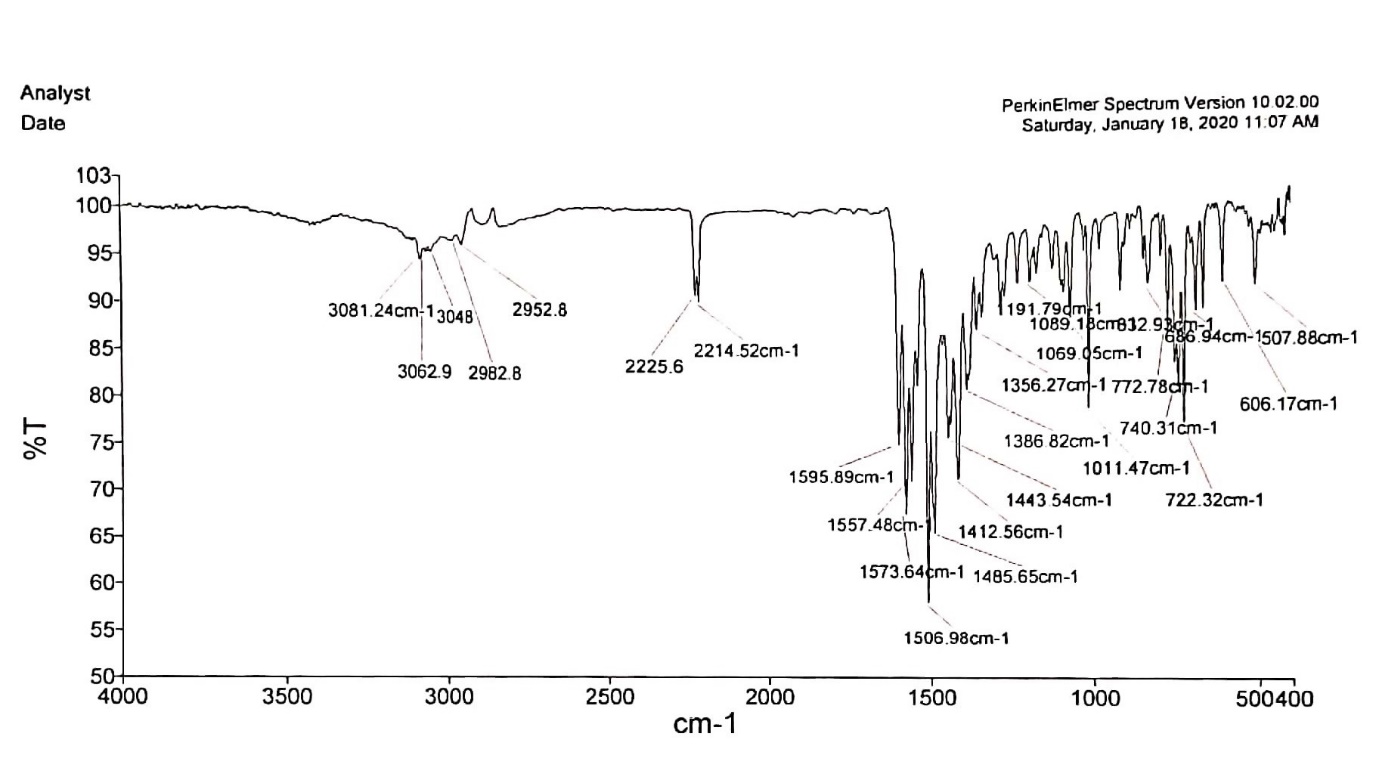

**Figure S16:** FT-IR spectrum of 4-(4-bromophenyl)-3-methyl-6-(1-methyl-1*H*-pyrrol-2-yl)-1-phenyl-1*H*-pyrazolo[3,4-*b*]pyridine-5-carbonitrile (6a)


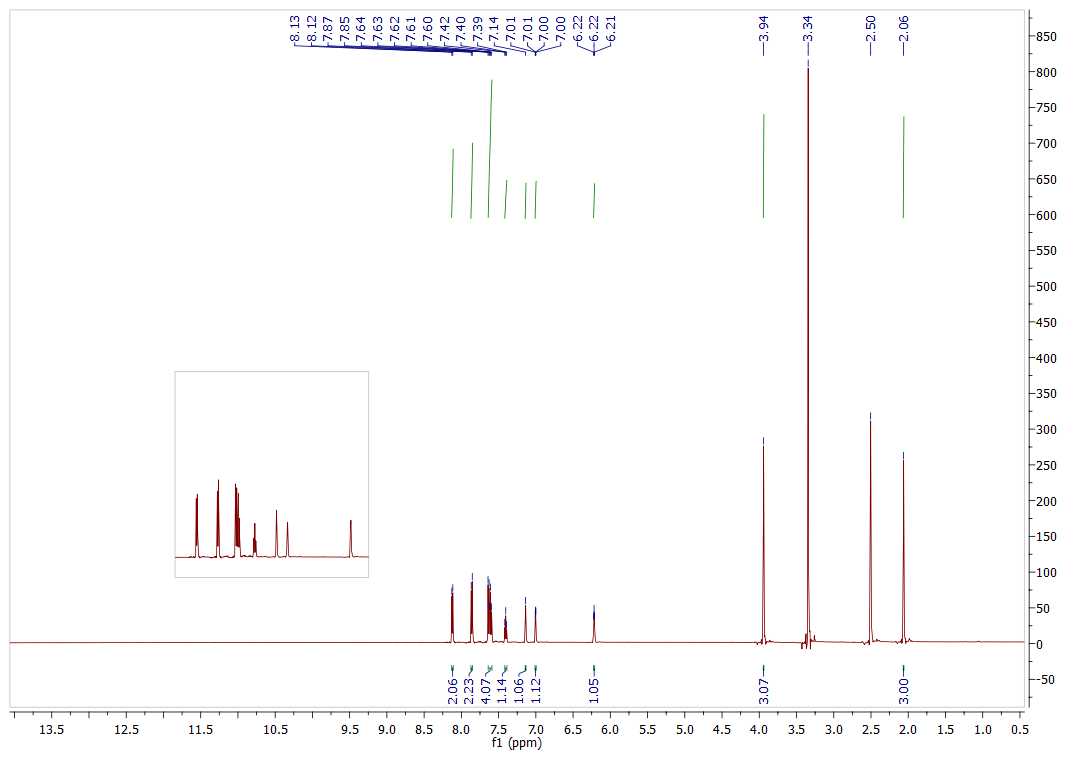

**Figure S17:** ^1^H NMR spectrum of 4-(4-bromophenyl)-3-methyl-6-(1-methyl-1*H*-pyrrol-2-yl)-1-phenyl-1*H*-pyrazolo[3,4-*b*]pyridine-5-carbonitrile (6a)


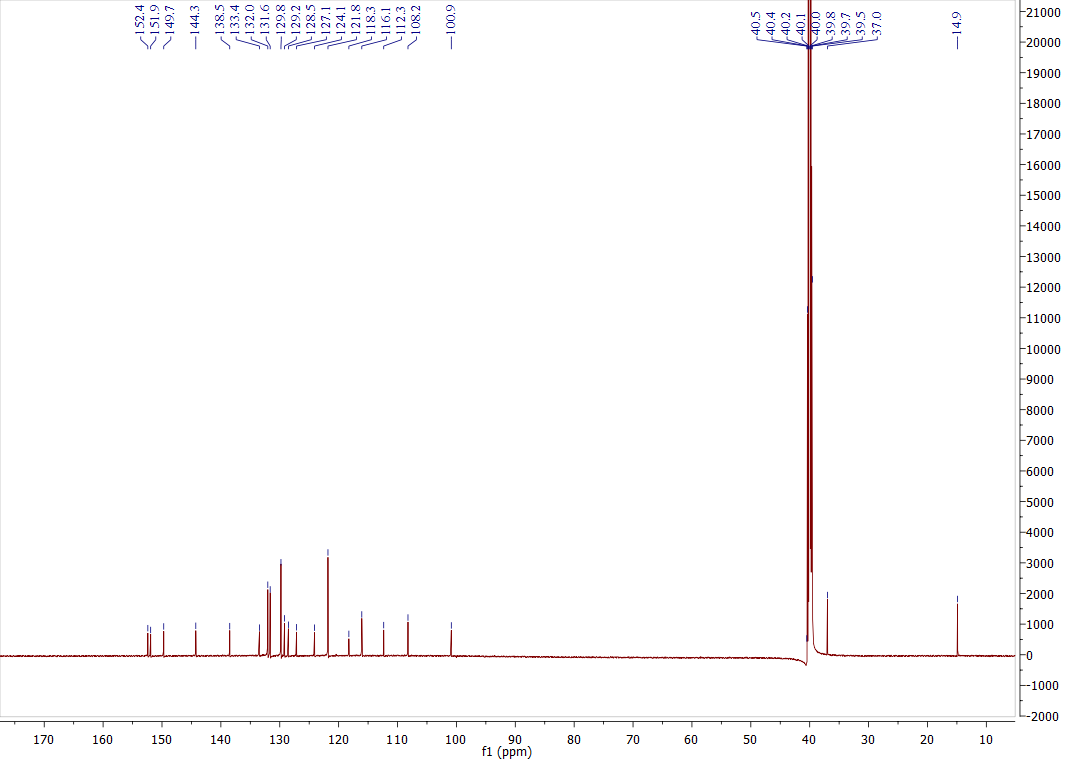

**Figure S18:** ^13^C NMR spectrum of 4-(4-bromophenyl)-3-methyl-6-(1-methyl-1*H*-pyrrol-2-yl)-1-phenyl-1*H*-pyrazolo[3,4-*b*]pyridine-5-carbonitrile (6a)


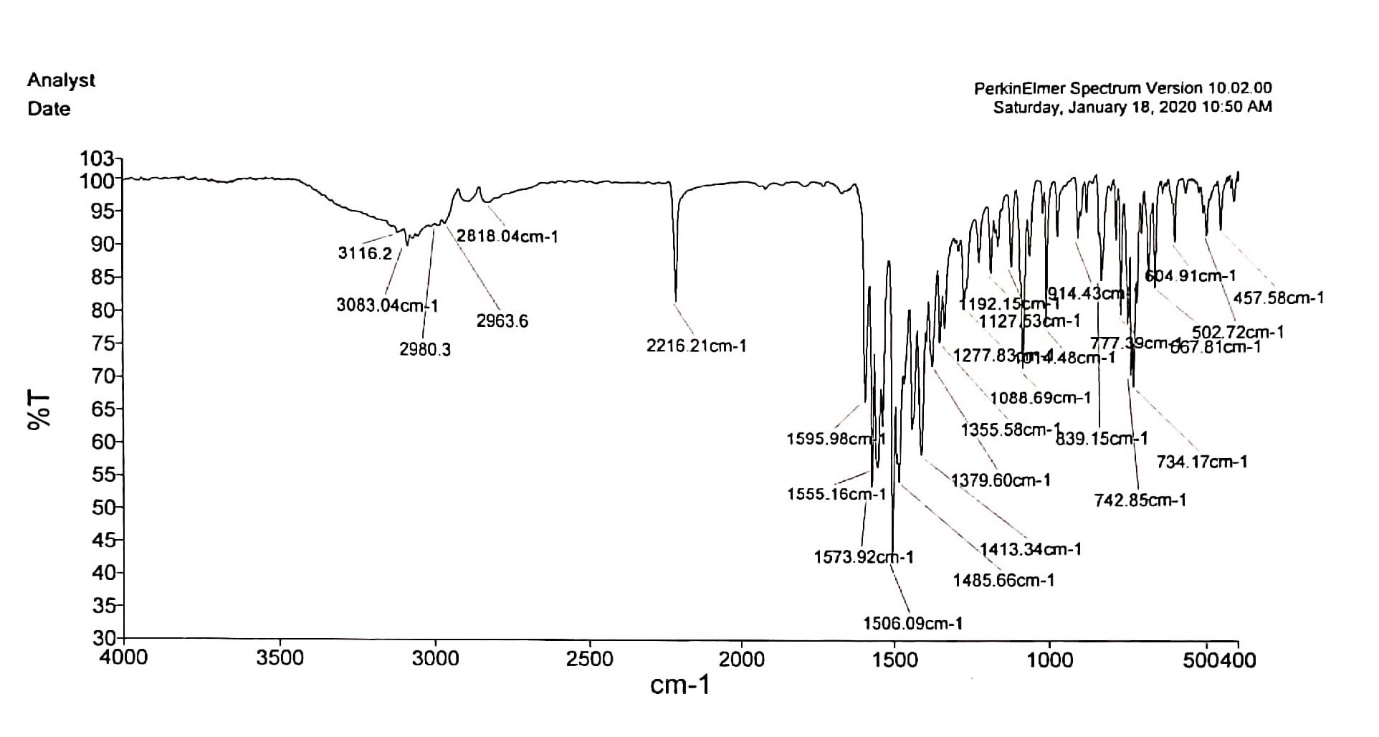

**Figure S19:** FT-IR spectrum of 4-(4-chlorophenyl)-3-methyl-6-(1-methyl-1*H*-pyrrol-2-yl)-1-phenyl-1*H*-pyrazolo[3,4-*b*]pyridine-5-carbonitrile (7a)


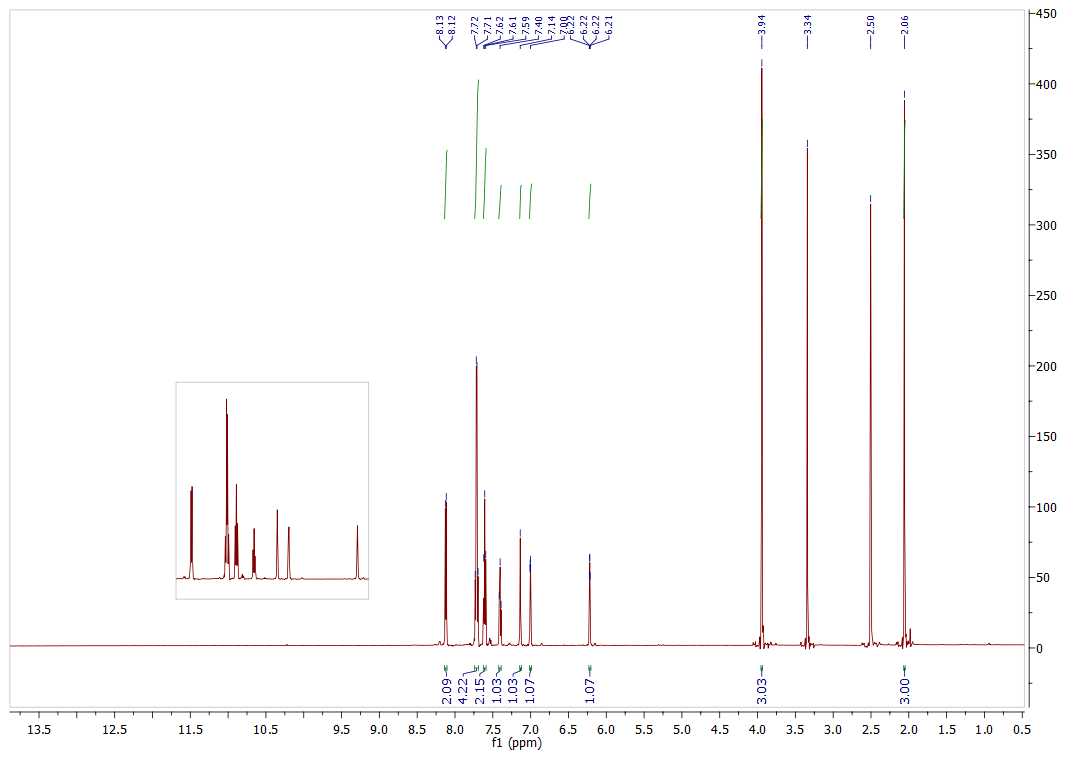

**Figure S20:** ^1^H NMR spectrum of 4-(4-chlorophenyl)-3-methyl-6-(1-methyl-1*H*-pyrrol-2-yl)-1-phenyl-1*H*-pyrazolo[3,4-*b*]pyridine-5-carbonitrile (7a)


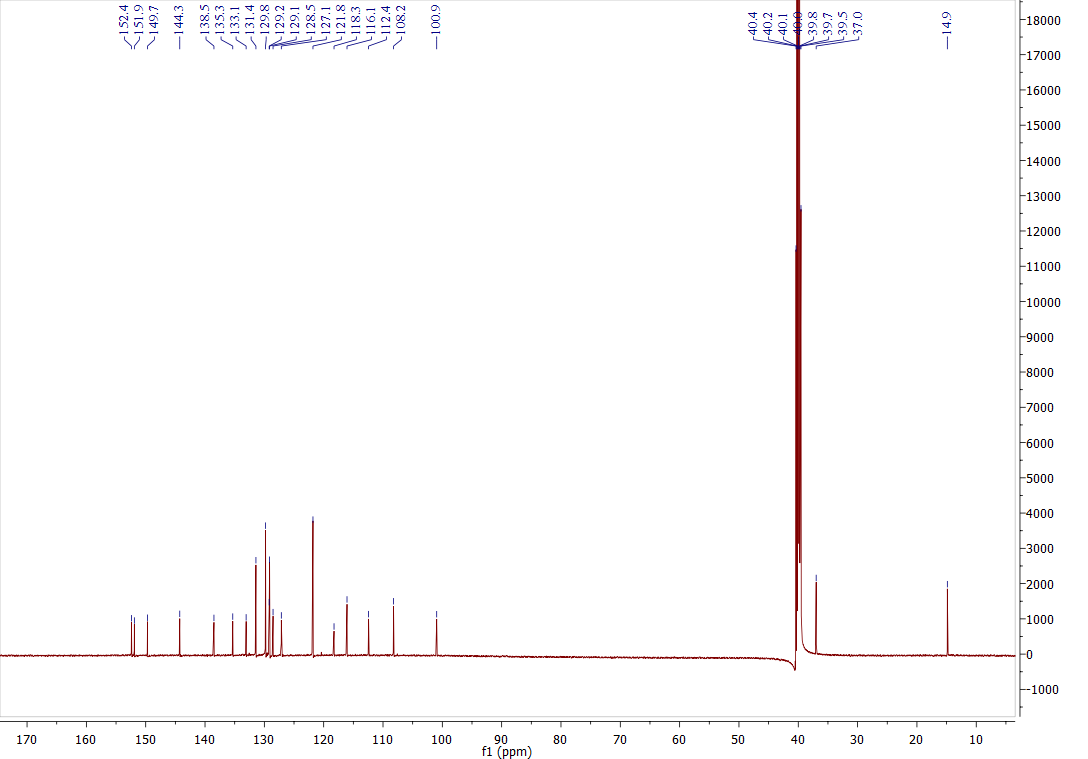

**Figure S21:** ^13^C NMR spectrum of 4-(4-chlorophenyl)-3-methyl-6-(1-methyl-1*H*-pyrrol-2-yl)-1-phenyl-1*H*-pyrazolo[3,4-*b*]pyridine-5-carbonitrile (7a)


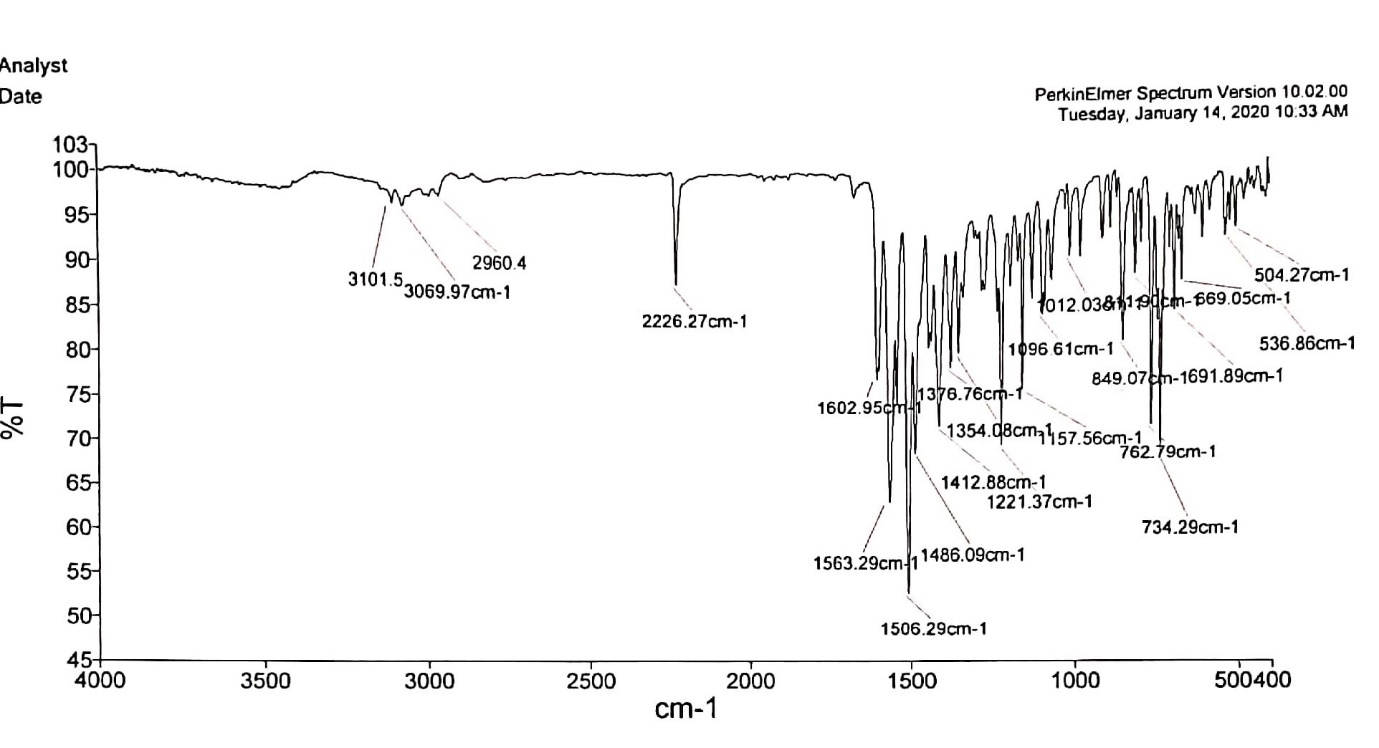

**Figure S22:** FT-IR spectrum of 4-(4-fluorophenyl)-3-methyl-6-(1-methyl-1*H*-pyrrol-2-yl)-1-phenyl-1*H*-pyrazolo[3,4-*b*]pyridine-5-carbonitrile (8a)


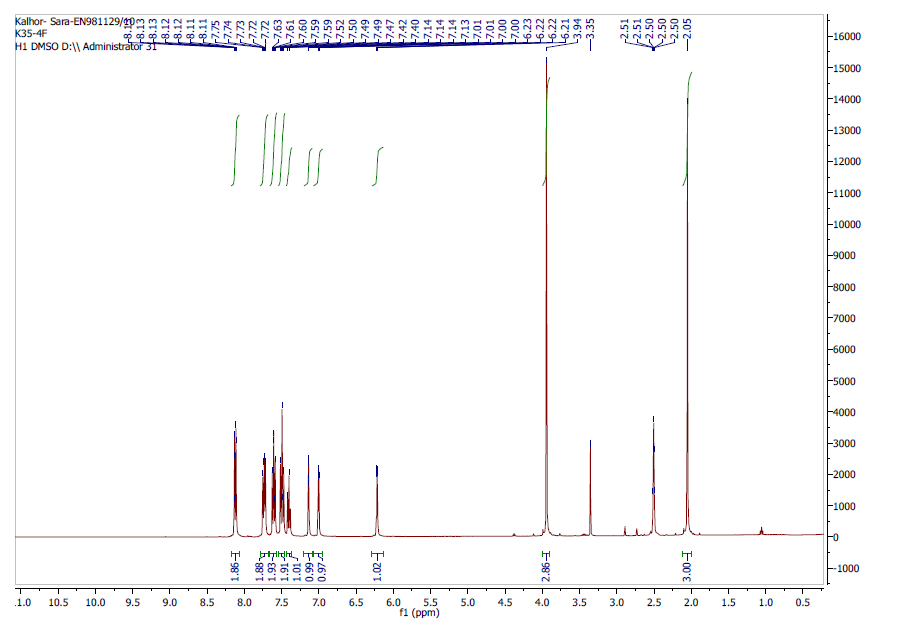

**Figure S23:** ^1^H NMR spectrum of 4-(4-fluorophenyl)-3-methyl-6-(1-methyl-1*H*-pyrrol-2-yl)-1-phenyl-1*H*-pyrazolo[3,4-*b*]pyridine-5-carbonitrile (8a)


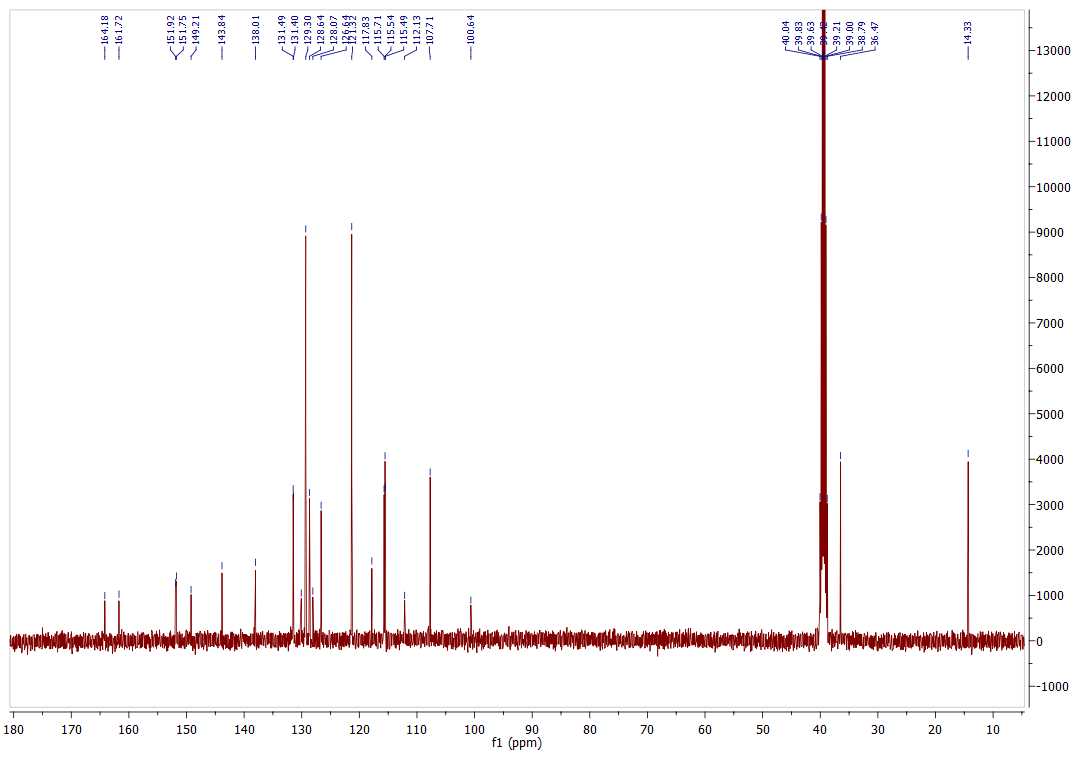

**Figure S24:** ^13^C NMR spectrum of 4-(4-fluorophenyl)-3-methyl-6-(1-methyl-1*H*-pyrrol-2-yl)-1-phenyl-1*H*-pyrazolo[3,4-*b*]pyridine-5-carbonitrile (8a)


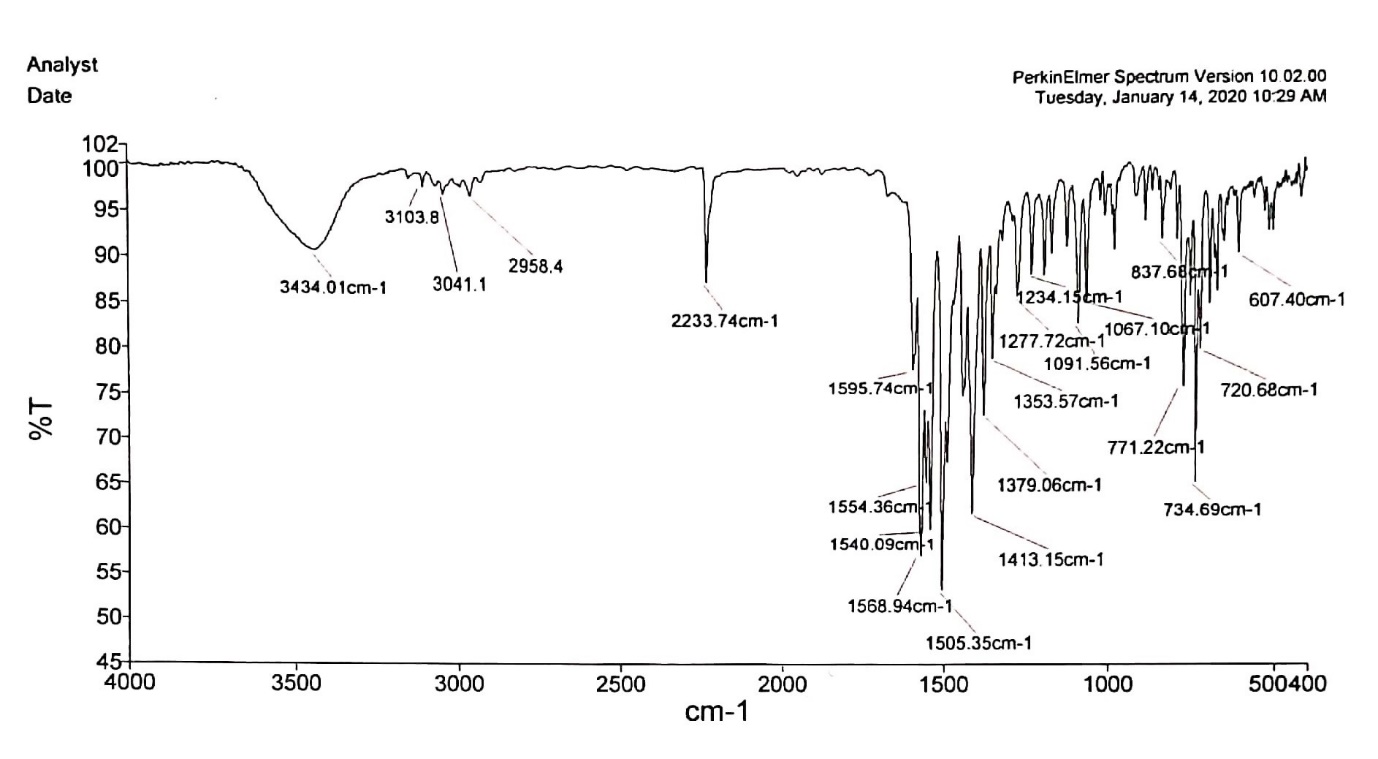

**Figure S25:** FT-IR spectrum of 3-methyl-6-(1-methyl-1*H*-pyrrol-2-yl)-1-phenyl-4-(pyridin-4-yl)-1*H*-pyrazolo[3,4-*b*]pyridine-5-carbonitrile (9a)


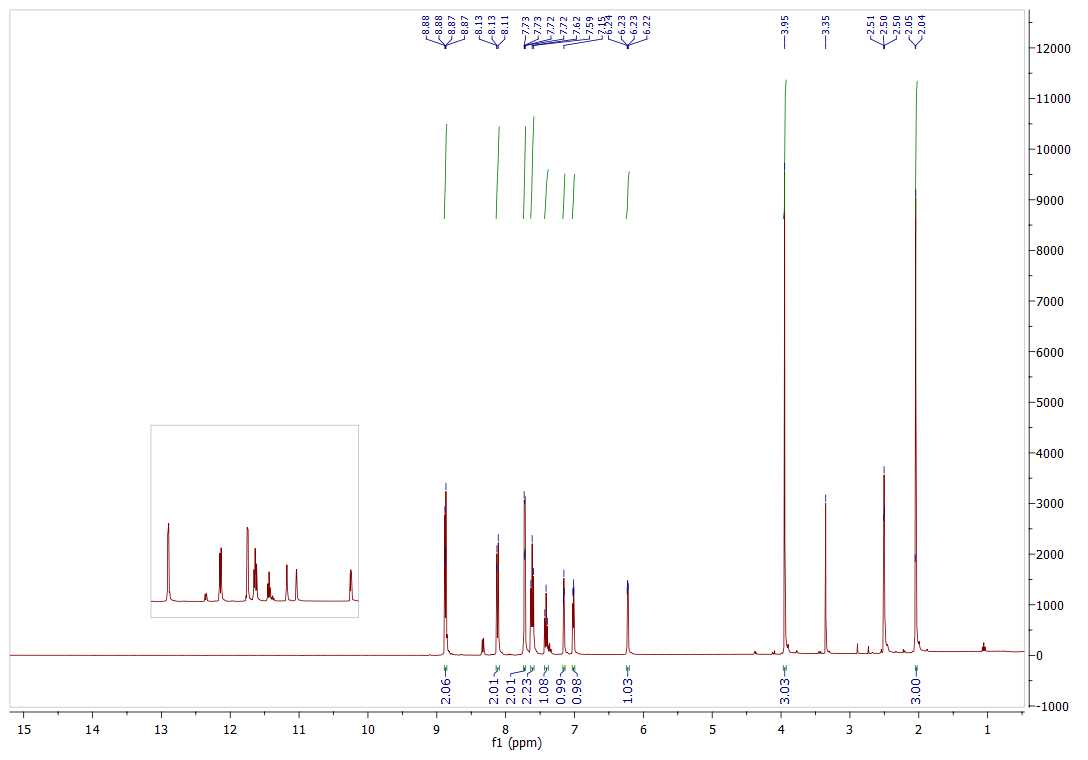

**Figure S26:** ^1^H NMR spectrum of 3-methyl-6-(1-methyl-1*H*-pyrrol-2-yl)-1-phenyl-4-(pyridin-4-yl)-1*H*-pyrazolo[3,4-*b*]pyridine-5-carbonitrile (9a)


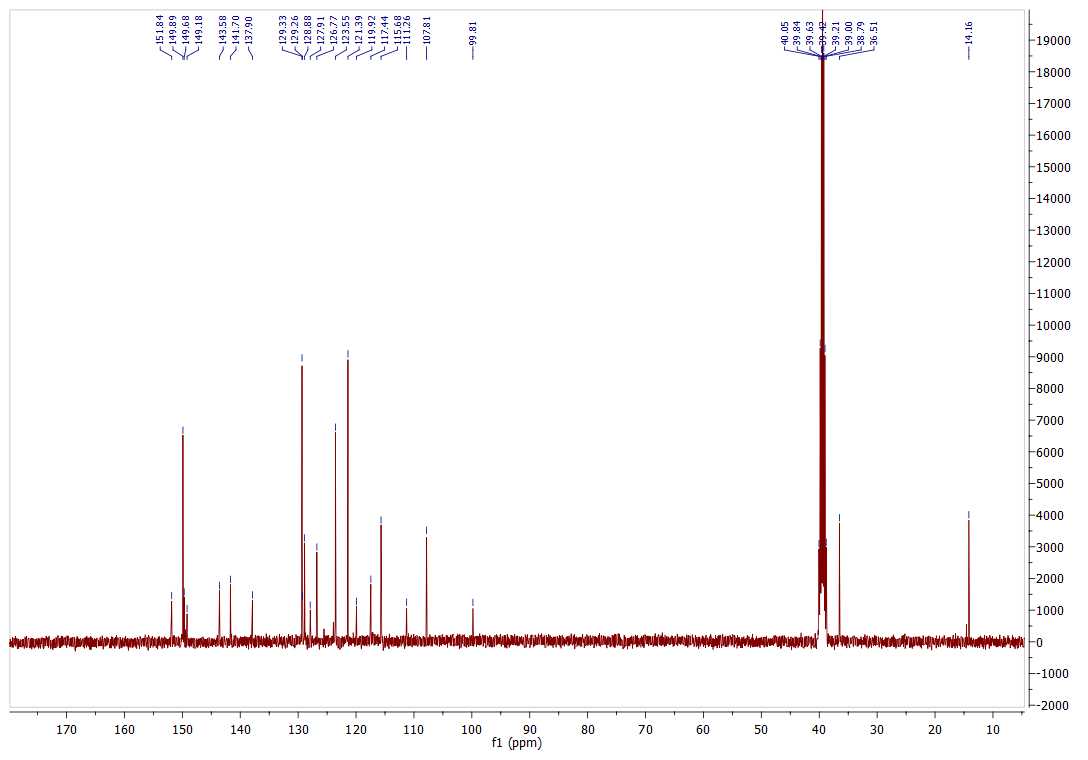

**Figure S27:** ^13^C NMR spectrum of 3-methyl-6-(1-methyl-1*H*-pyrrol-2-yl)-1-phenyl-4-(pyridin-4-yl)-1*H*-pyrazolo[3,4-*b*]pyridine-5-carbonitrile (9a)


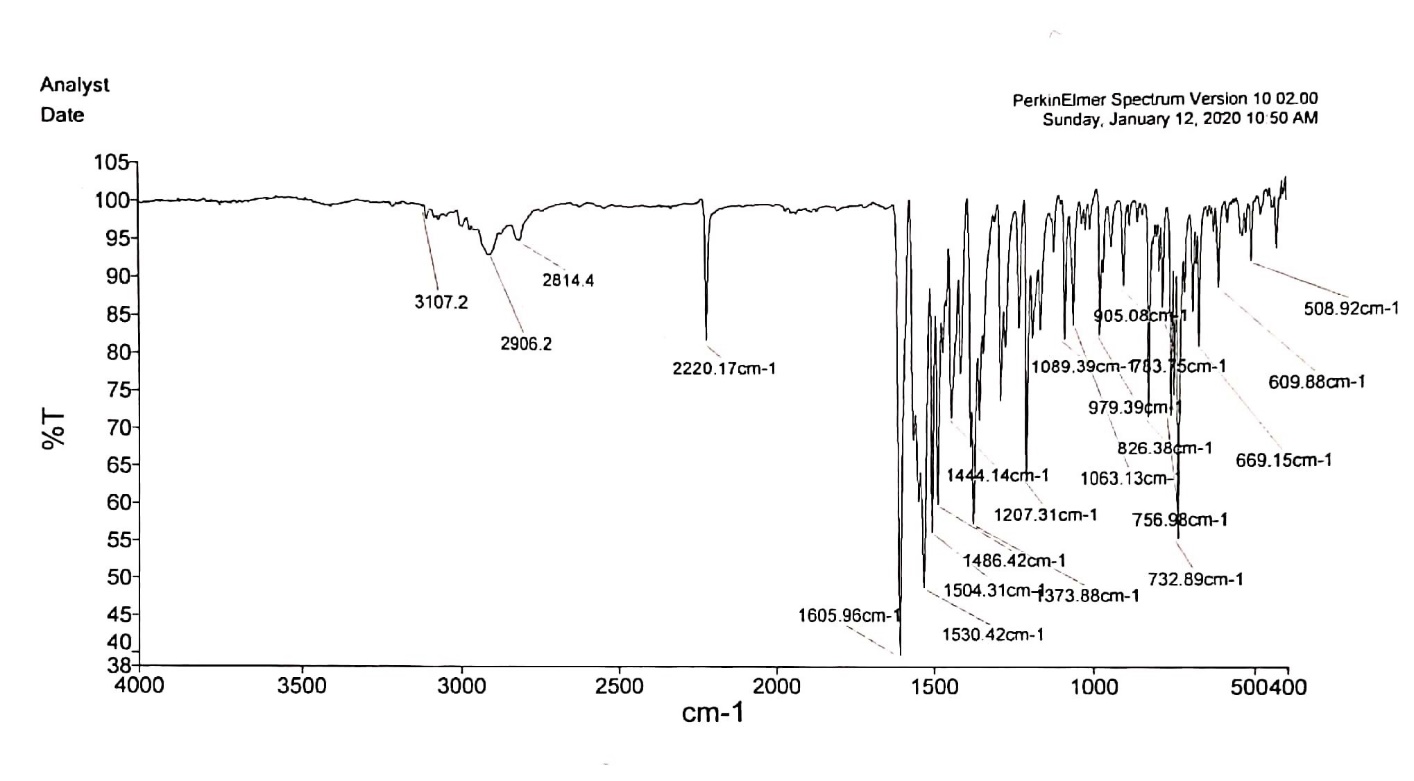

**Figure S28:** FT-IR spectrum of 4-(4-(dimethylamino)phenyl)-3-methyl-6-(1-methyl-1*H*-pyrrol-2-yl)-1-phenyl-1*H*-pyrazolo[3,4-*b*]pyridine-5-carbonitrile (10a)


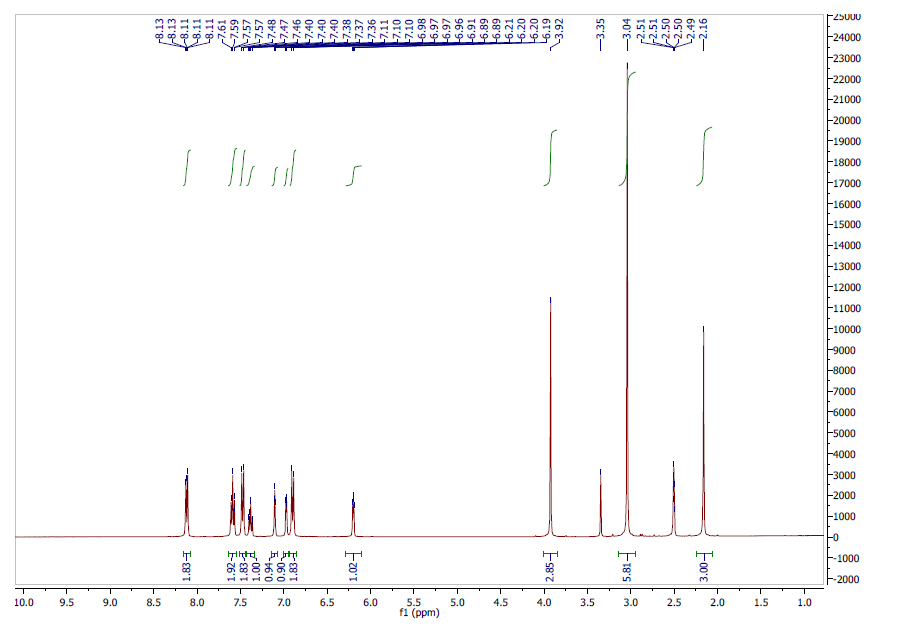

**Figure S29:** ^1^H NMR spectrum of 4-(4-(dimethylamino)phenyl)-3-methyl-6-(1-methyl-1*H*-pyrrol-2-yl)-1-phenyl-1*H*-pyrazolo[3,4-*b*]pyridine-5-carbonitrile (10a)


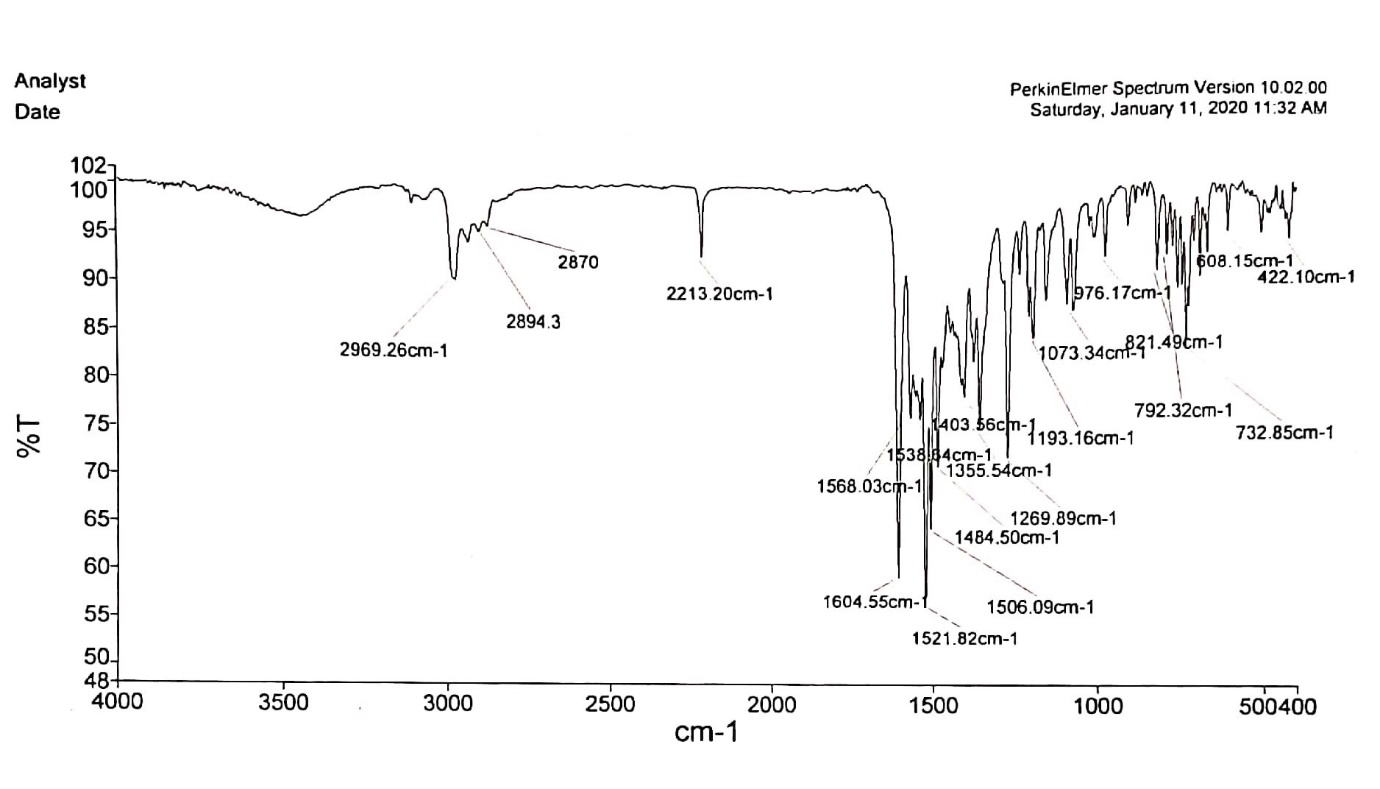

**Figure S30:** FT-IR spectrum of 4-(4-(diethylamino)phenyl)-3-methyl-6-(1-methyl-1*H*-pyrrol-2-yl)-1-phenyl-1*H*-pyrazolo[3,4-*b*]pyridine-5-carbonitrile (11a)


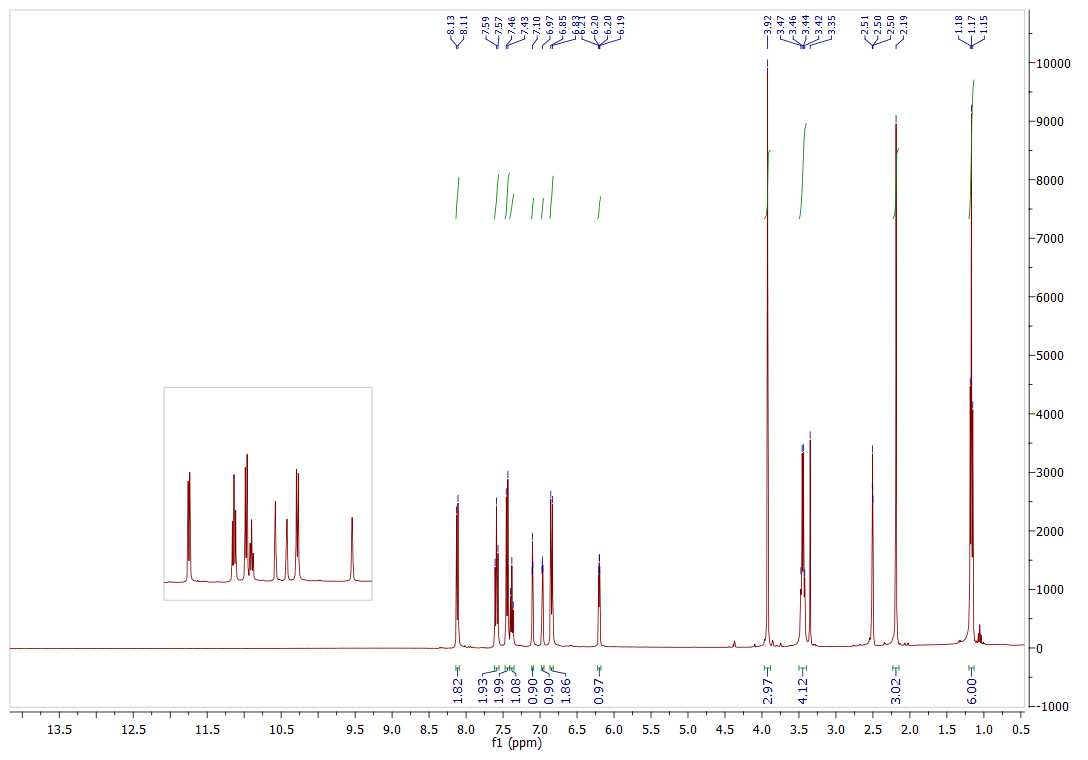

**Figure S31:** ^1^H NMR spectrum of 4-(4-(diethylamino)phenyl)-3-methyl-6-(1-methyl-1*H*-pyrrol-2-yl)-1-phenyl-1*H*-pyrazolo[3,4-*b*]pyridine-5-carbonitrile (11a)


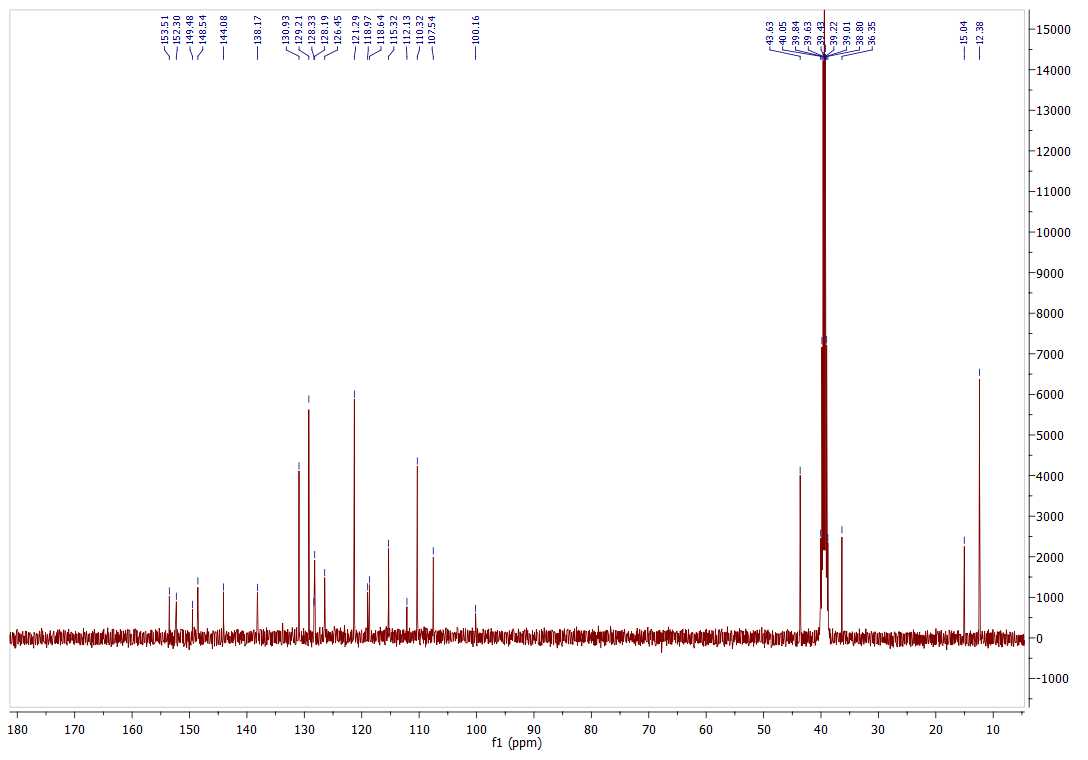

**Figure S32:** ^13^C NMR spectrum of 4-(4-(diethylamino)phenyl)-3-methyl-6-(1-methyl-1*H*-pyrrol-2-yl)-1-phenyl-1*H*-pyrazolo[3,4-*b*]pyridine-5-carbonitrile (11a)


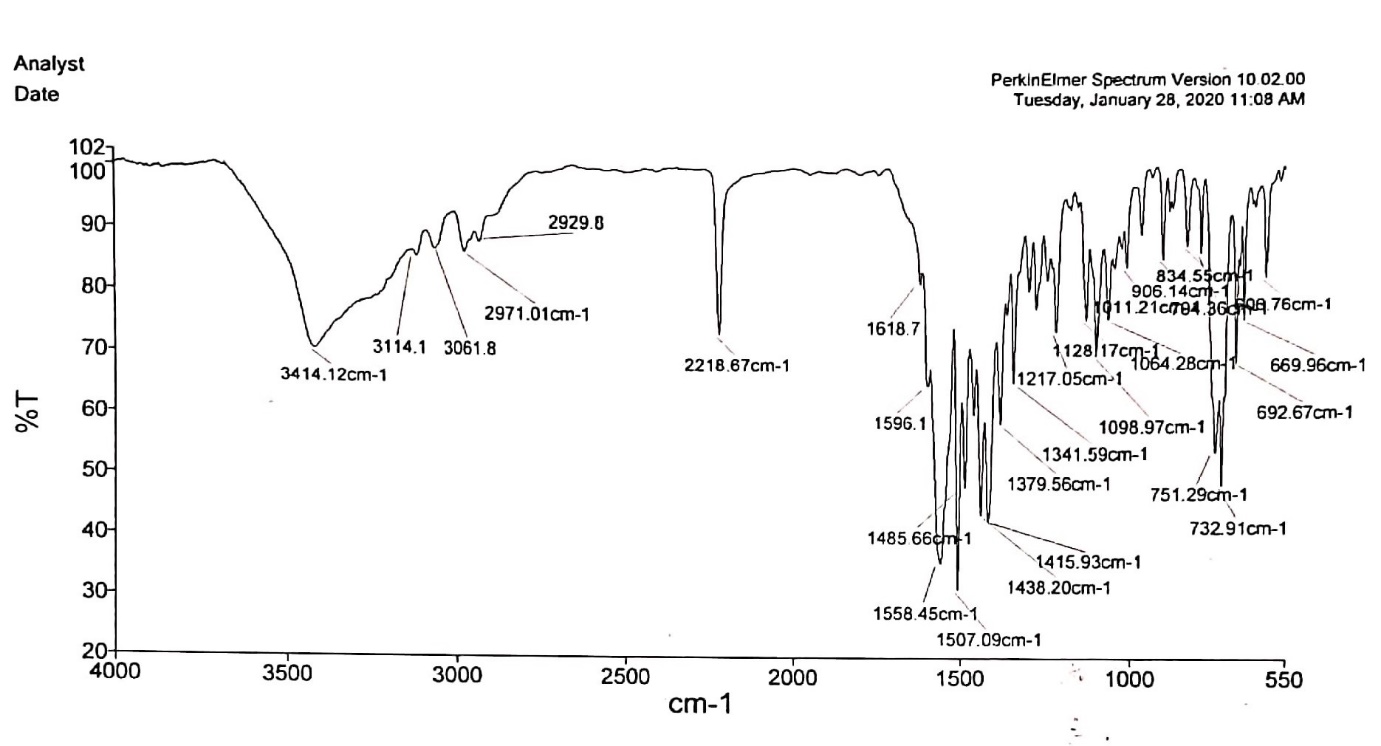

**Figure S33:** FT-IR spectrum of 4-(1*H*-indol-3-yl)-3-methyl-6-(1-methyl-1*H*-pyrrol-2-yl)-1-phenyl-1*H*-pyrazolo[3,4-*b*]pyridine-5-carbonitrile (12a)


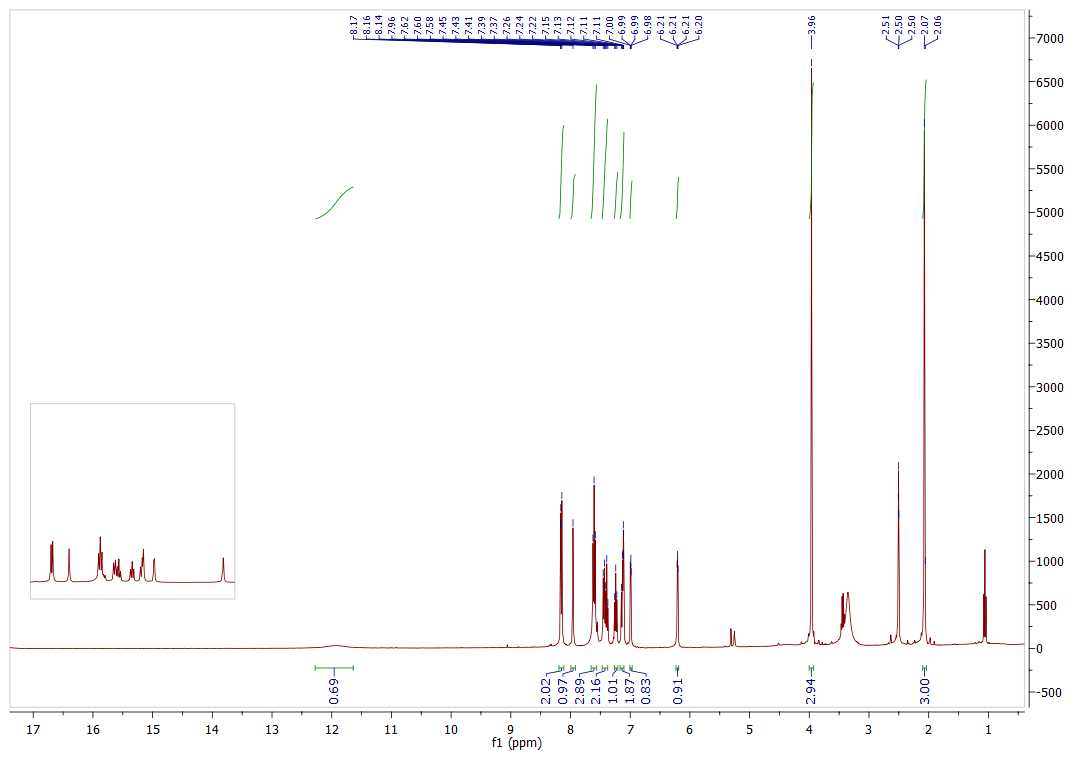

**Figure S34:** ^1^H NMR spectrum of 4-(1*H*-indol-3-yl)-3-methyl-6-(1-methyl-1*H*-pyrrol-2-yl)-1-phenyl-1*H*-pyrazolo[3,4-*b*]pyridine-5-carbonitrile (12a)


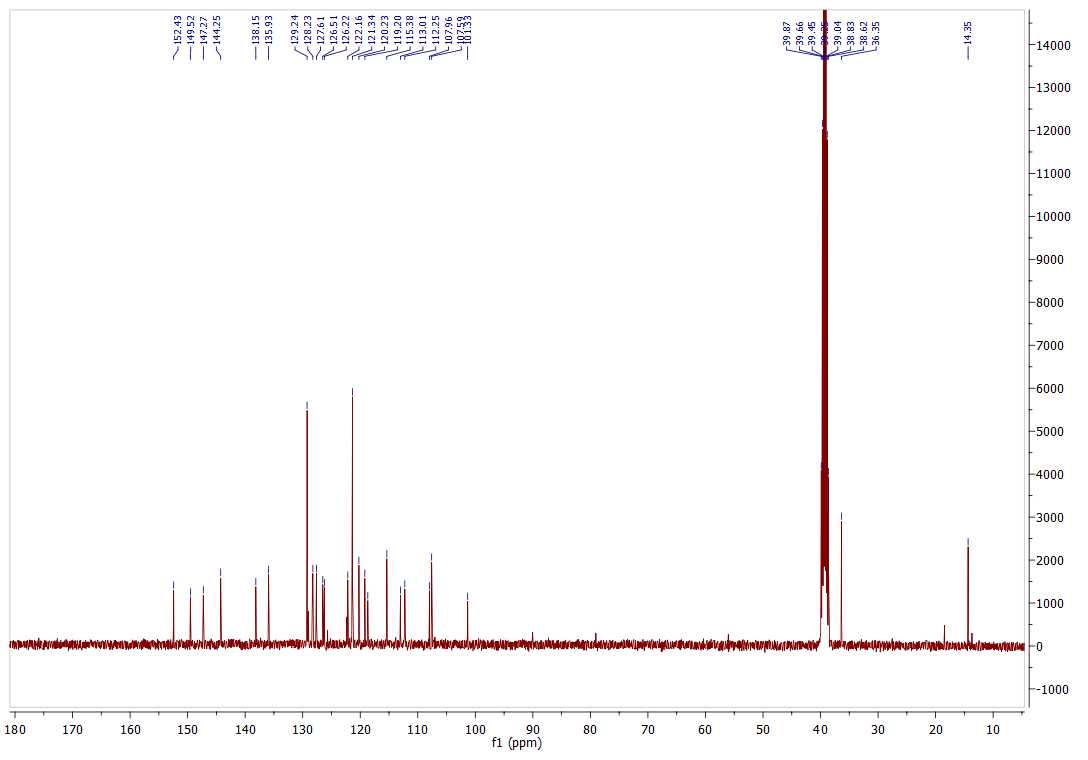

**Figure S35:** ^13^C NMR spectrum of 4-(1*H*-indol-3-yl)-3-methyl-6-(1-methyl-1*H*-pyrrol-2-yl)-1-phenyl-1*H*-pyrazolo[3,4-*b*]pyridine-5-carbonitrile (12a)


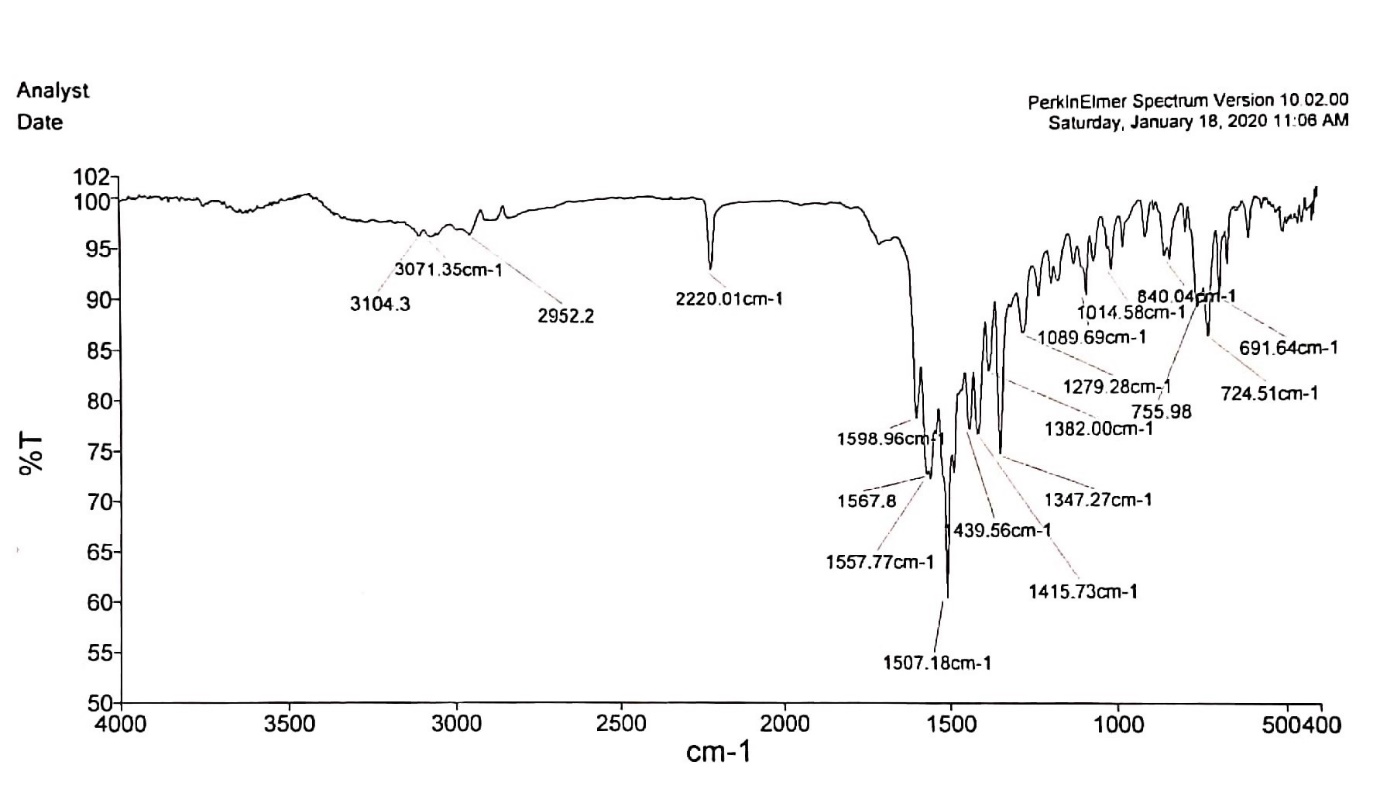

**Figure S36:** FT-IR spectrum of 3-methyl-6-(1-methyl-1*H*-pyrrol-2-yl)-4-(4-nitrophenyl)-1-phenyl-1*H*-pyrazolo[3,4-*b*]pyridine-5-carbonitrile (13a)


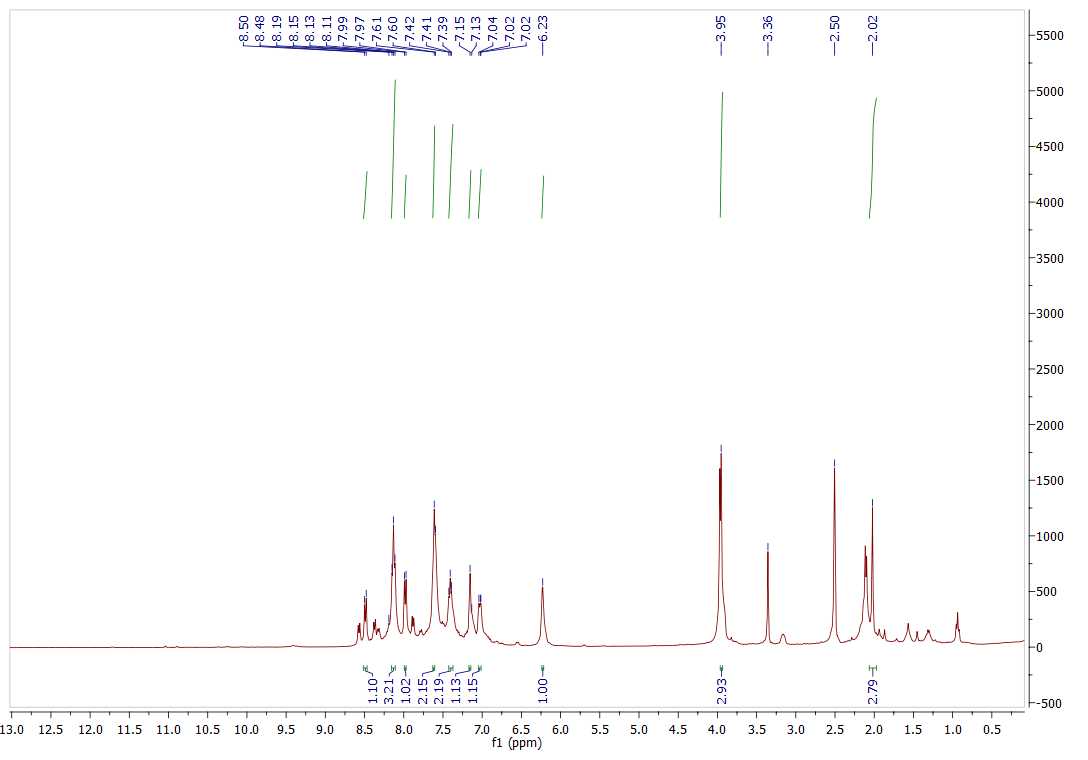

**Figure S37:** ^1^H NMR spectrum of 3-methyl-6-(1-methyl-1*H*-pyrrol-2-yl)-4-(4-nitrophenyl)-1-phenyl-1*H*-pyrazolo[3,4-*b*]pyridine-5-carbonitrile (13a)


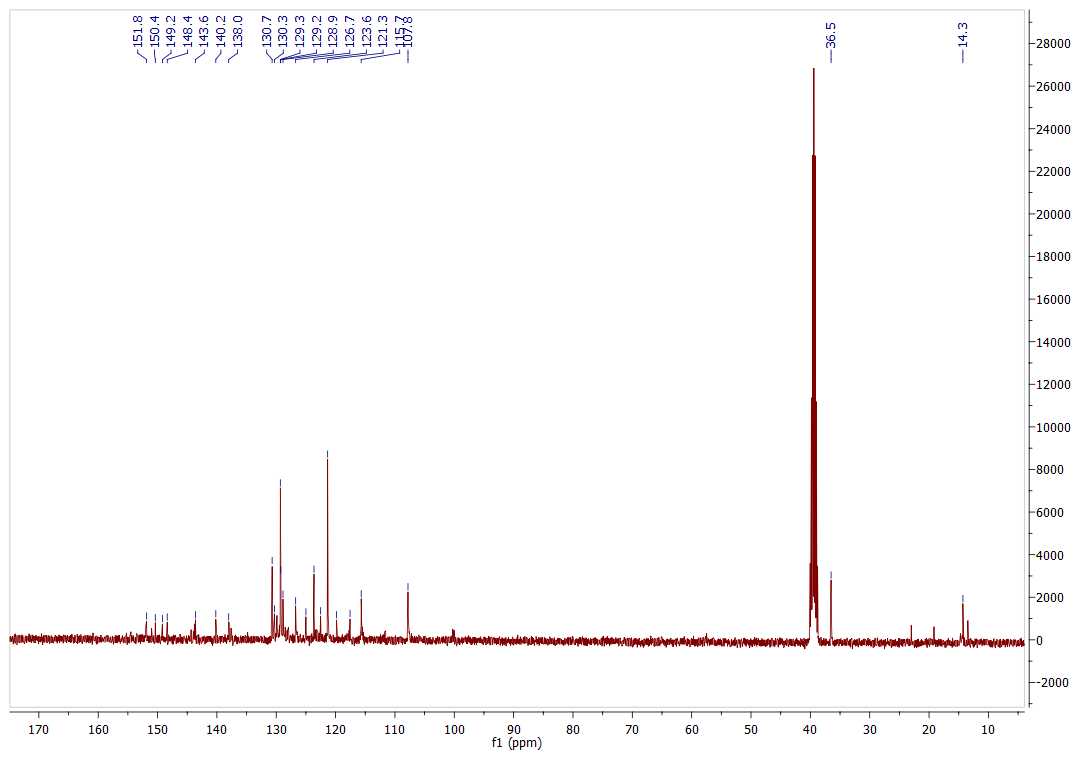

**Figure S38:** ^13^C NMR spectrum of 3-methyl-6-(1-methyl-1*H*-pyrrol-2-yl)-4-(4-nitrophenyl)-1-phenyl-1*H*-pyrazolo[3,4-*b*]pyridine-5-carbonitrile (13a)


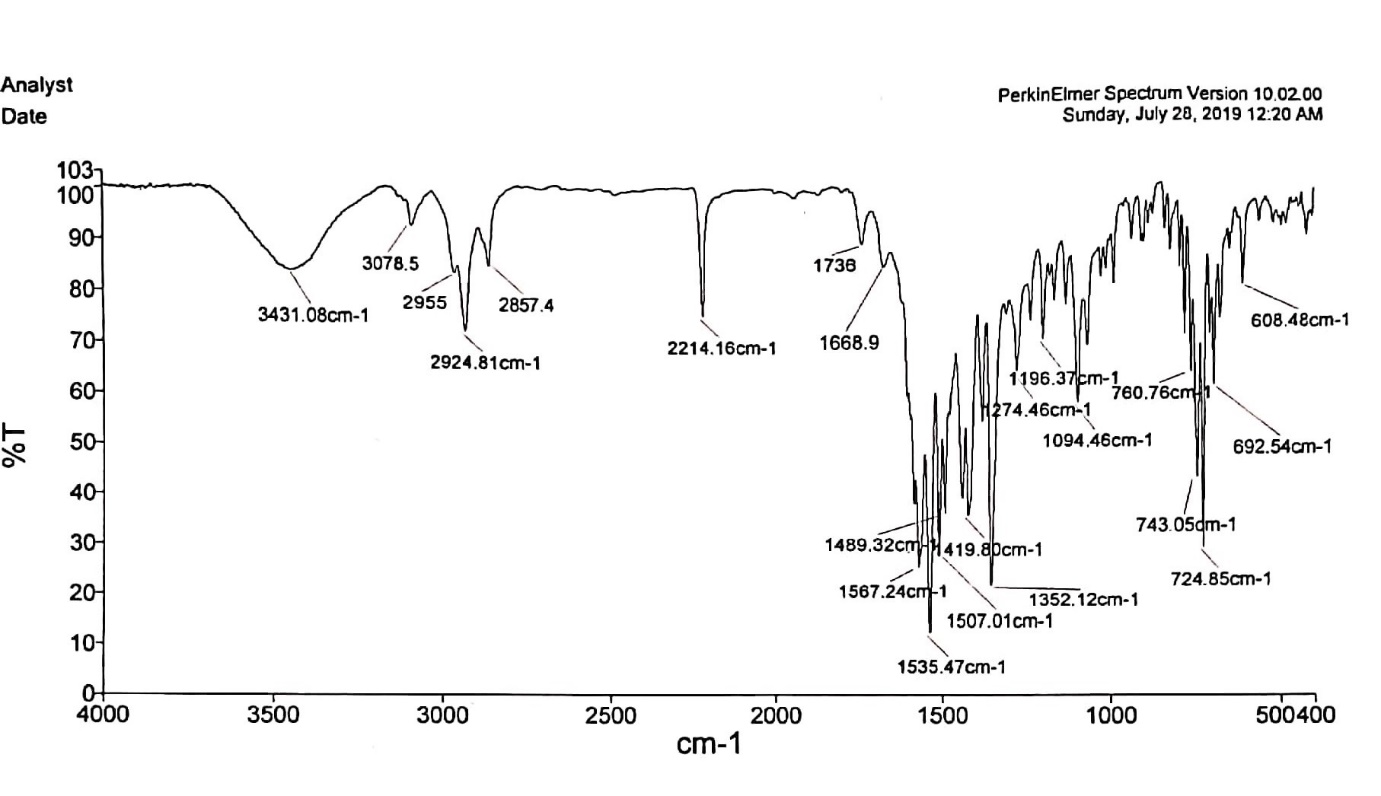

**Figure S39:** FT-IR spectrum of 3-methyl-6-(1-methyl-1*H*-pyrrol-2-yl)-4-(3-nitrophenyl)-1-phenyl-1*H*-pyrazolo[3,4-*b*]pyridine-5-carbonitrile (14a)


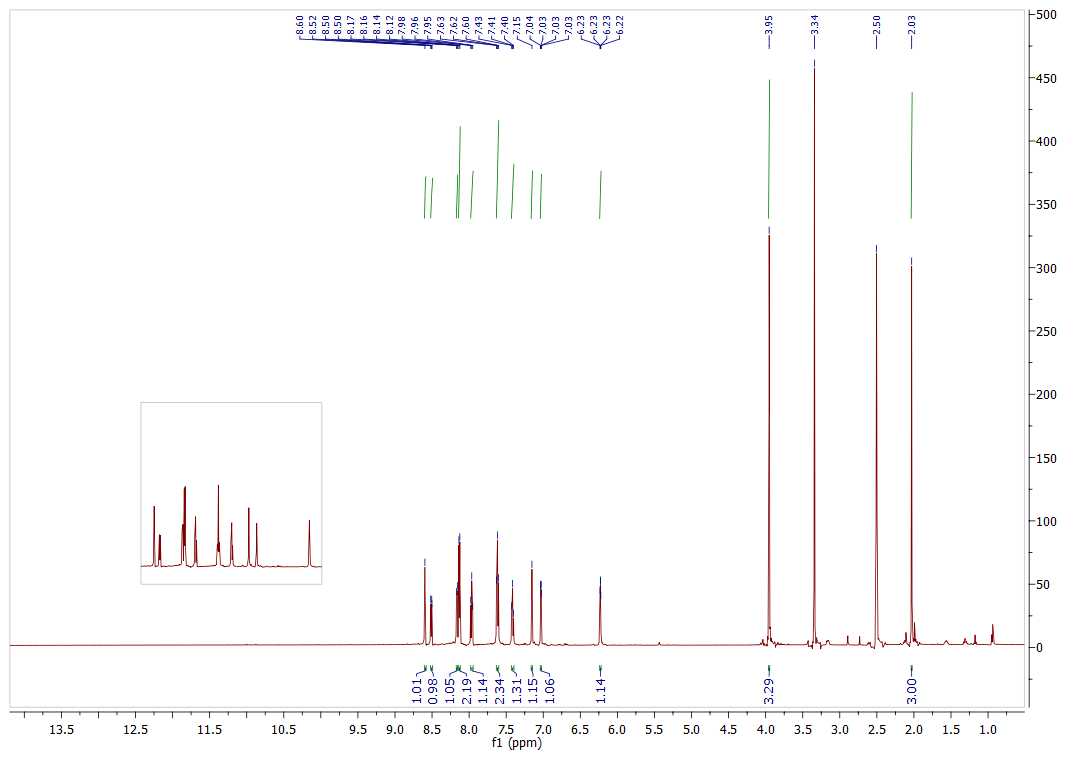

**Figure S40:** ^1^H NMR spectrum of 3-methyl-6-(1-methyl-1*H*-pyrrol-2-yl)-4-(3-nitrophenyl)-1-phenyl-1*H*-pyrazolo[3,4-*b*]pyridine-5-carbonitrile (14a)


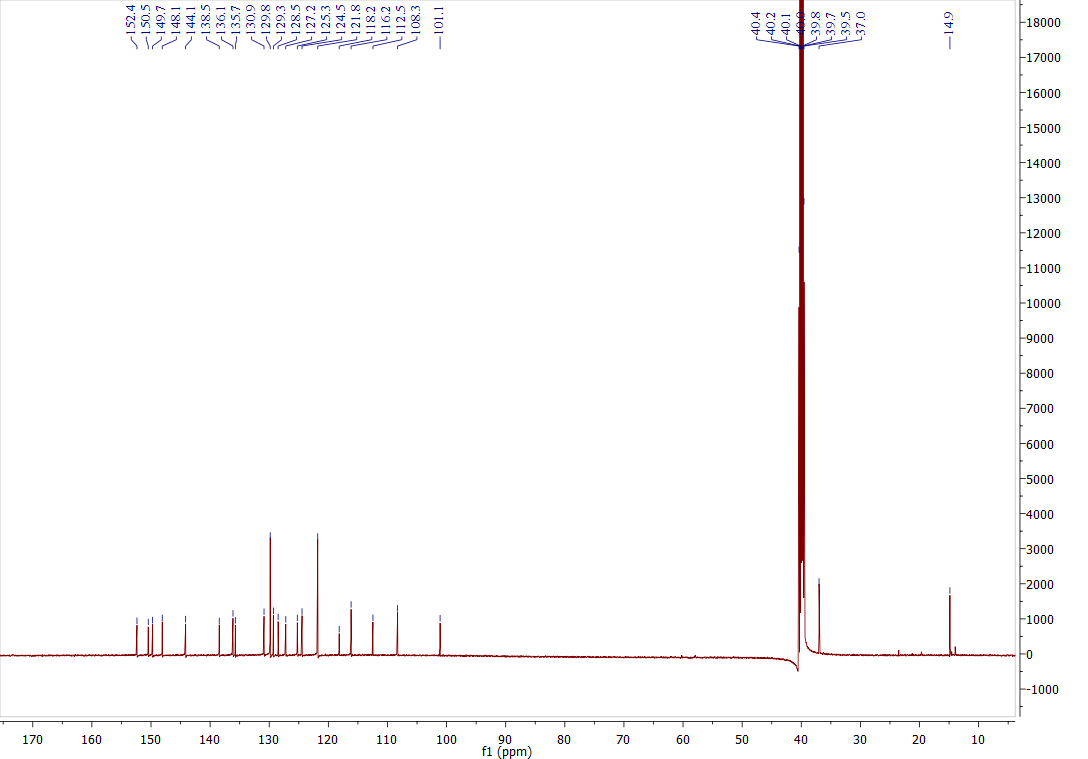

**Figure S41:** ^13^C NMR spectrum of 3-methyl-6-(1-methyl-1*H*-pyrrol-2-yl)-4-(3-nitrophenyl)-1-phenyl-1*H*-pyrazolo[3,4-*b*]pyridine-5-carbonitrile (14a)


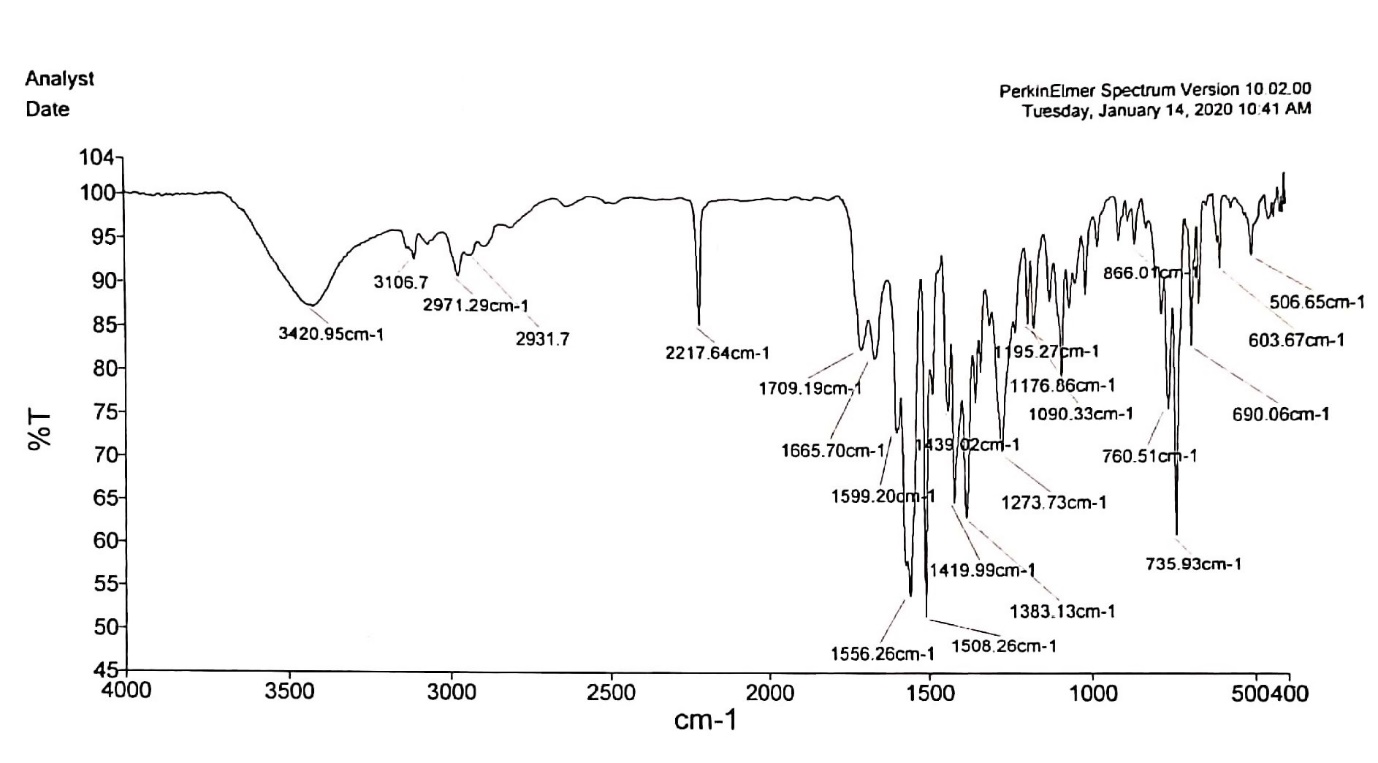

**Figure S42:** FT-IR spectrum of 4-(5-cyano-3-methyl-6-(1-methyl-1*H*-pyrrol-2-yl)-1-phenyl-1*H*-pyrazolo[3,4-*b*]pyridin-4-yl)benzoic acid (15a)


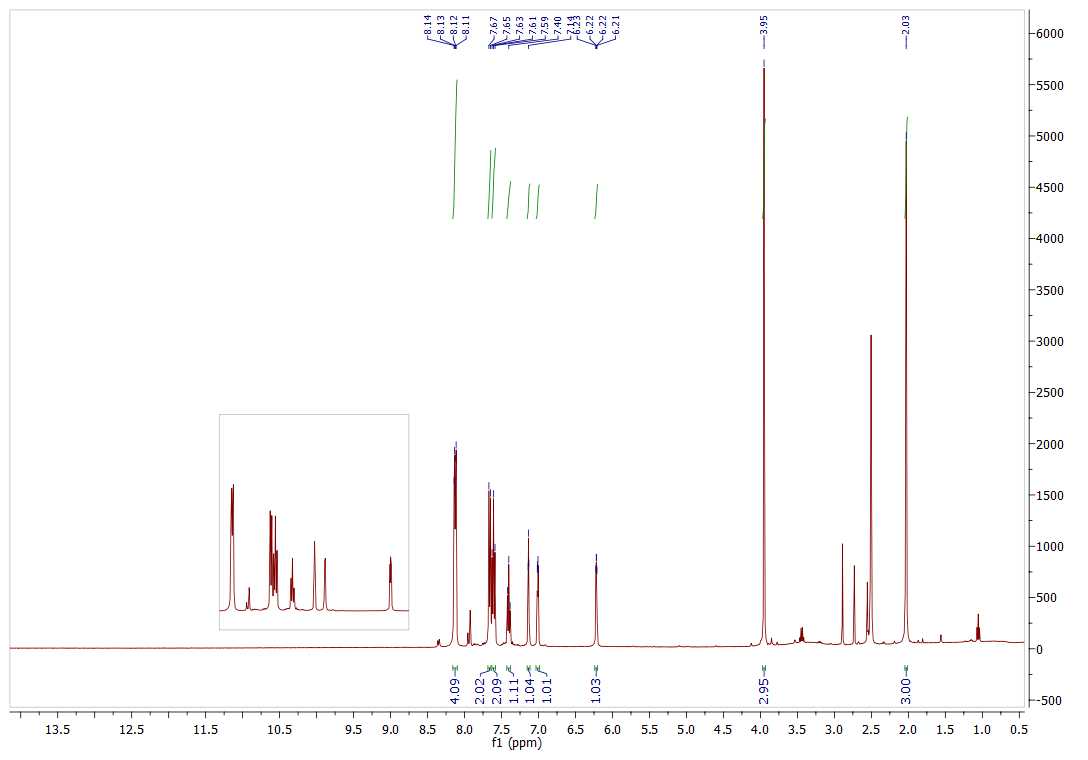

**Figure S43:** ^1^H NMR spectrum of 4-(5-cyano-3-methyl-6-(1-methyl-1*H*-pyrrol-2-yl)-1-phenyl-1*H*-pyrazolo[3,4-*b*]pyridin-4-yl)benzoic acid (15a)


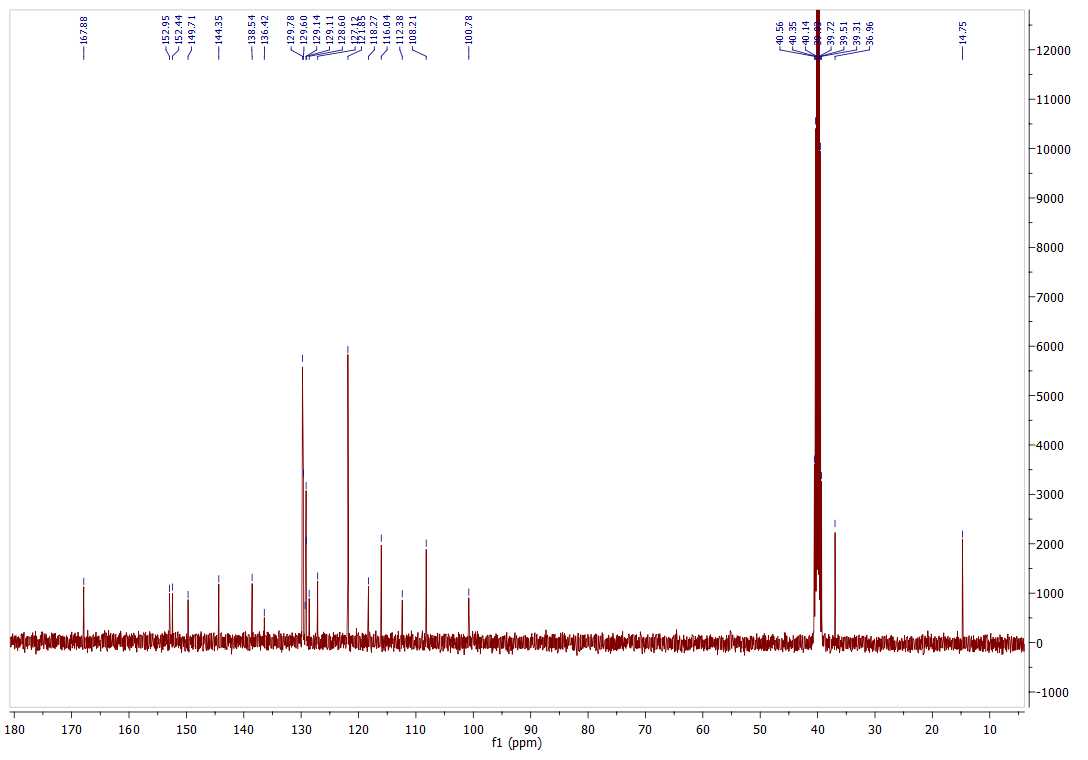

**Figure S44:** ^13^C NMR spectrum of 4-(5-cyano-3-methyl-6-(1-methyl-1*H*-pyrrol-2-yl)-1-phenyl-1*H*-pyrazolo[3,4-*b*]pyridin-4-yl)benzoic acid (15a)


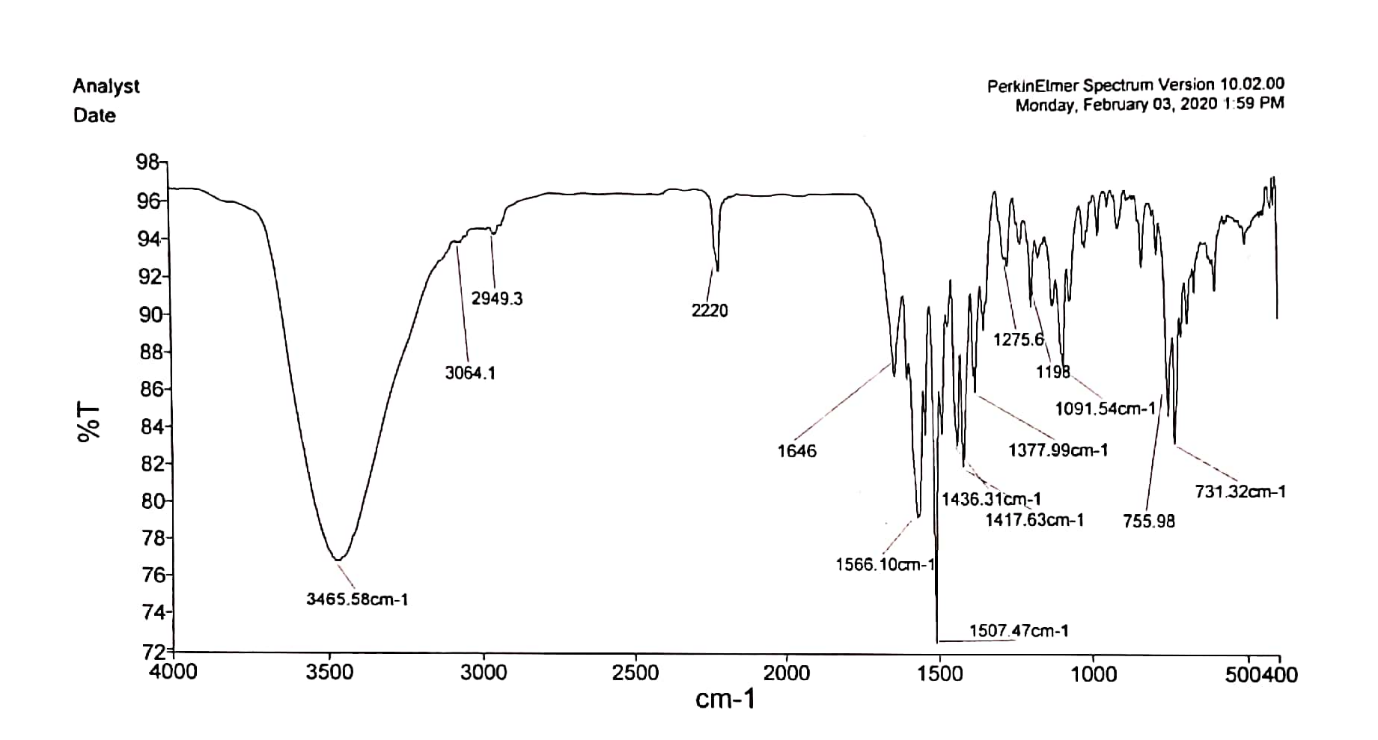

**Figure S45:** FT-IR spectrum of 4,4'-(1,4-phenylene)bis(3-methyl-6-(1-methyl-1*H*-pyrrol-2-yl)-1-phenyl-1*H*-pyrazolo[3,4-*b*]pyridine-5-carbonitrile) (16a)


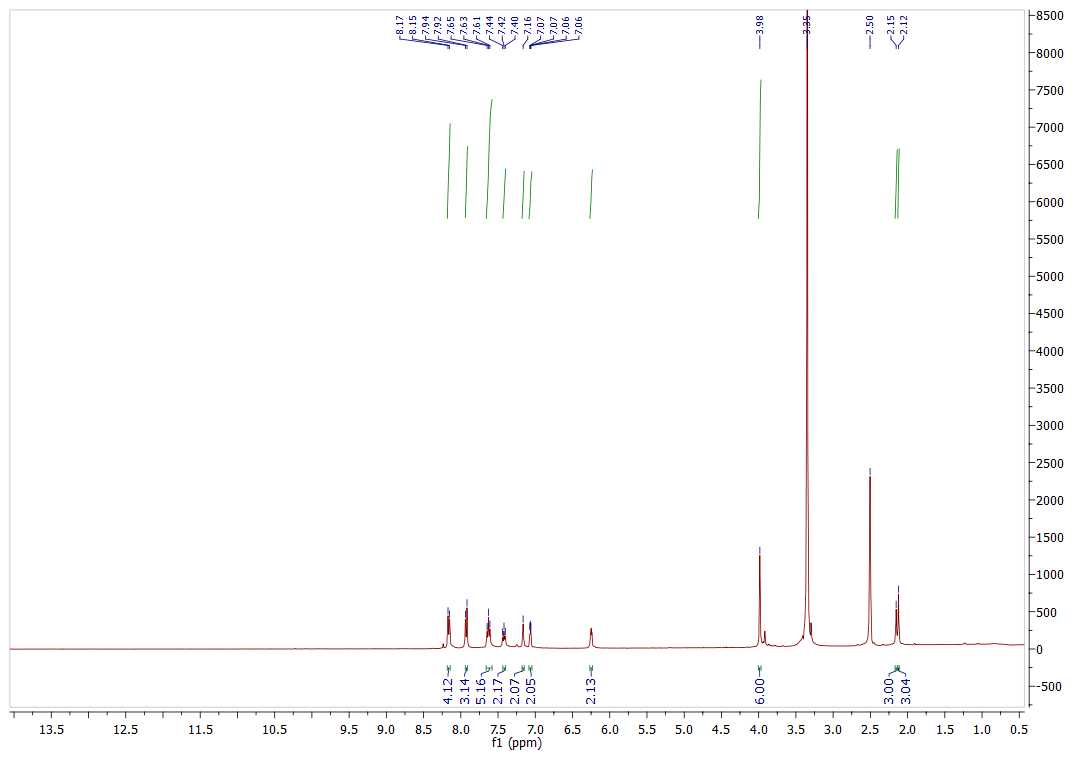

**Figure S46:** ^1^H NMR spectrum of 4,4'-(1,4-phenylene)bis(3-methyl-6-(1-methyl-1*H*-pyrrol-2-yl)-1-phenyl-1*H*-pyrazolo[3,4-*b*]pyridine-5-carbonitrile) (16a)


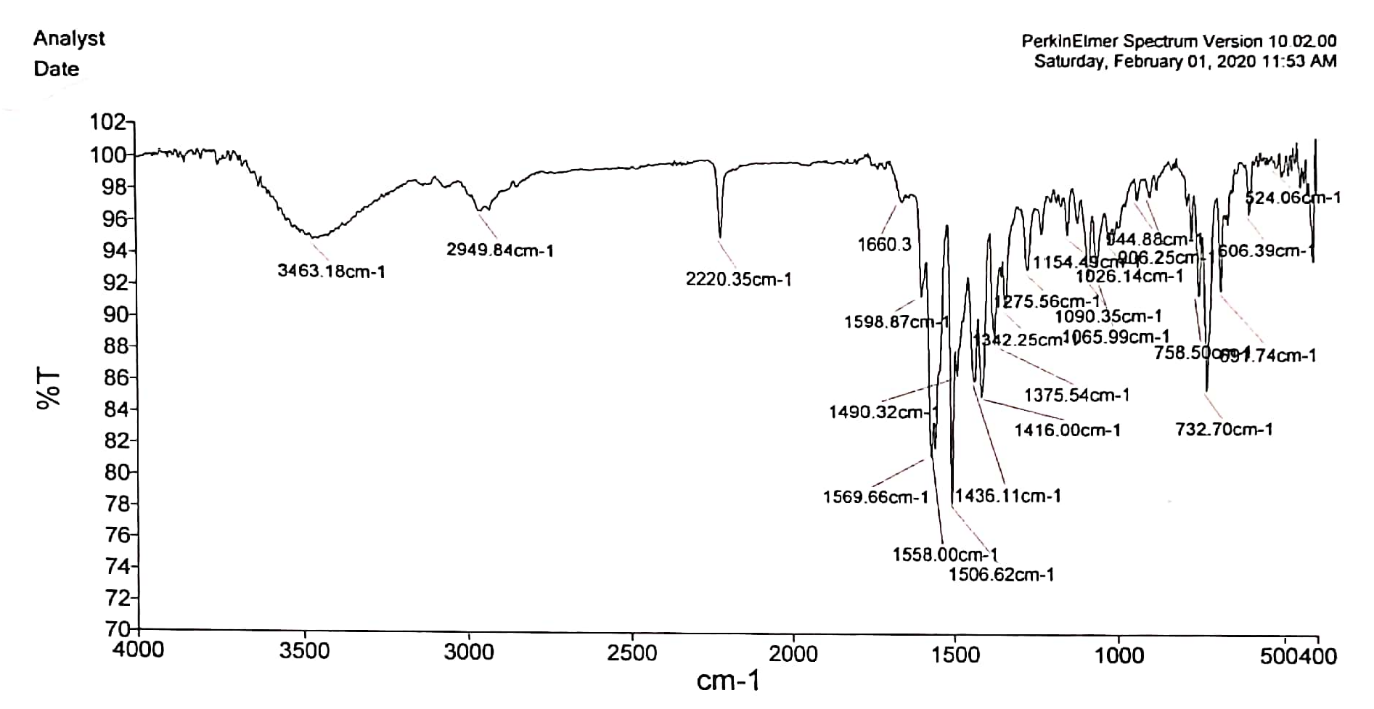

**Figure S47:** FT-IR spectrum of 4,4'-(1,3-phenylene)bis(3-methyl-6-(1-methyl-1*H*-pyrrol-2-yl)-1-phenyl-1*H*-pyrazolo[3,4-*b*]pyridine-5-carbonitrile) (17a)


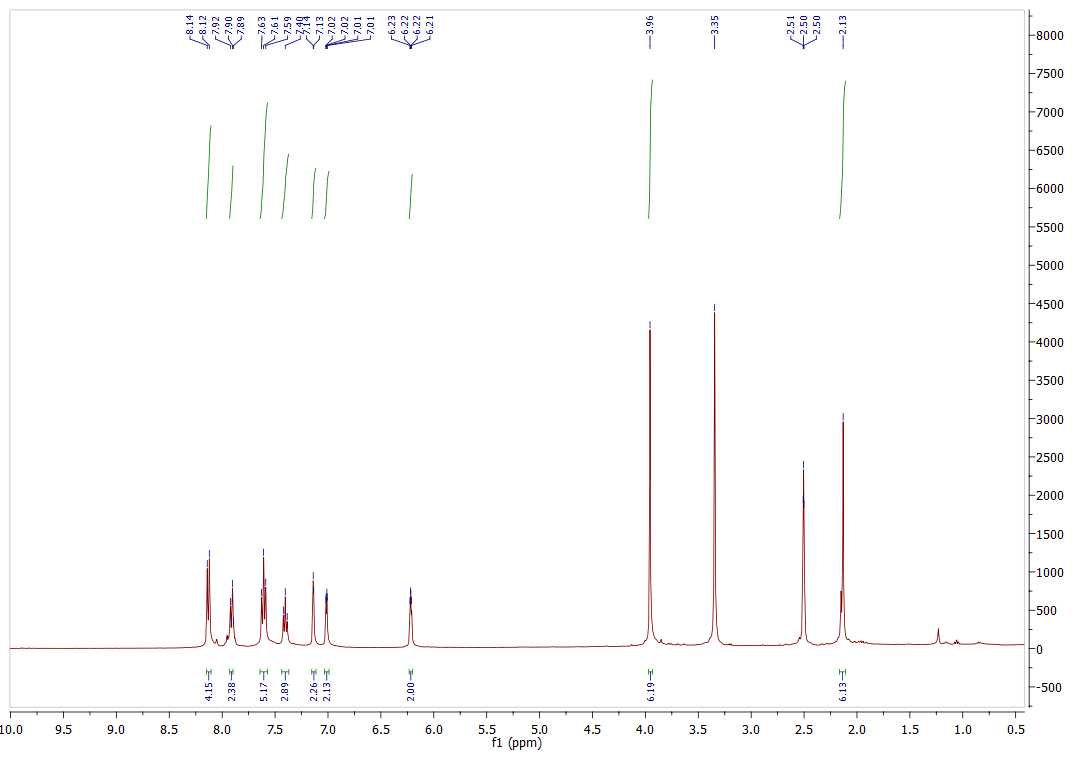

**Figure S48:** ^1^H NMR spectrum of 4,4'-(1,3-phenylene)bis(3-methyl-6-(1-methyl-1*H*-pyrrol-2-yl)-1-phenyl-1*H*-pyrazolo[3,4-*b*]pyridine-5-carbonitrile) (17a)


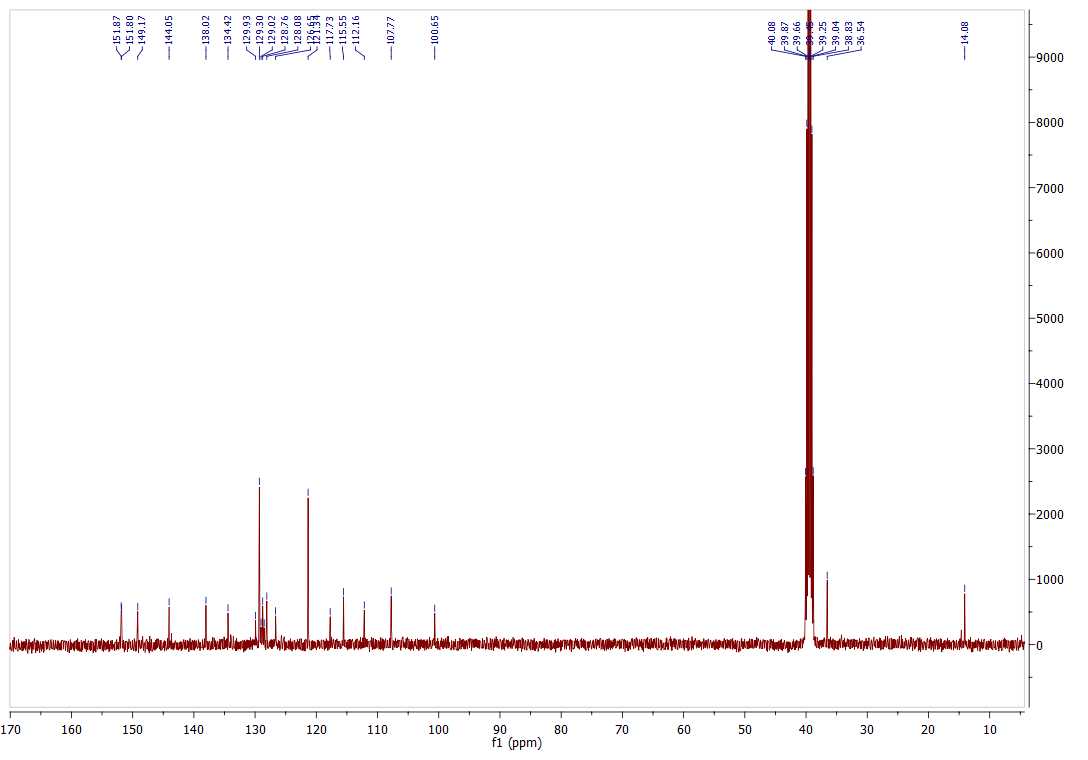

**Figure S49:** ^13^C NMR spectrum of 4,4'-(1,3-phenylene)bis(3-methyl-6-(1-methyl-1*H*-pyrrol-2-yl)-1-phenyl-1*H*-pyrazolo[3,4-*b*]pyridine-5-carbonitrile) (17a)


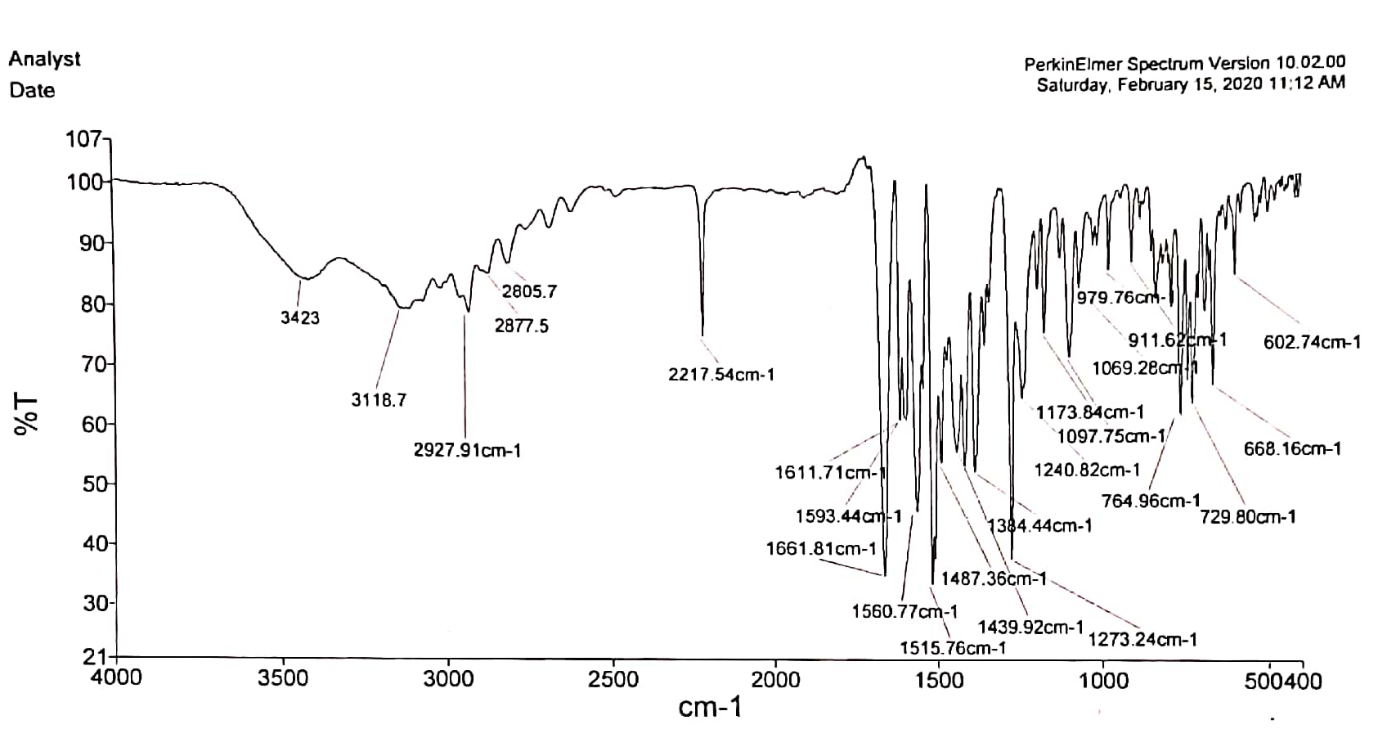

**Figure S50:** FT-IR spectrum of 4,4',4''-(((1,3,5-triazine-2,4,6-triyl)tris(oxy))tris(benzene-4,1-diyl))tris(3-methyl-6-(1-methyl-1*H*-pyrrol-2-yl)-1-phenyl-1*H*-pyrazolo[3,4-*b*]pyridine-5-carbonitrile) (18a)


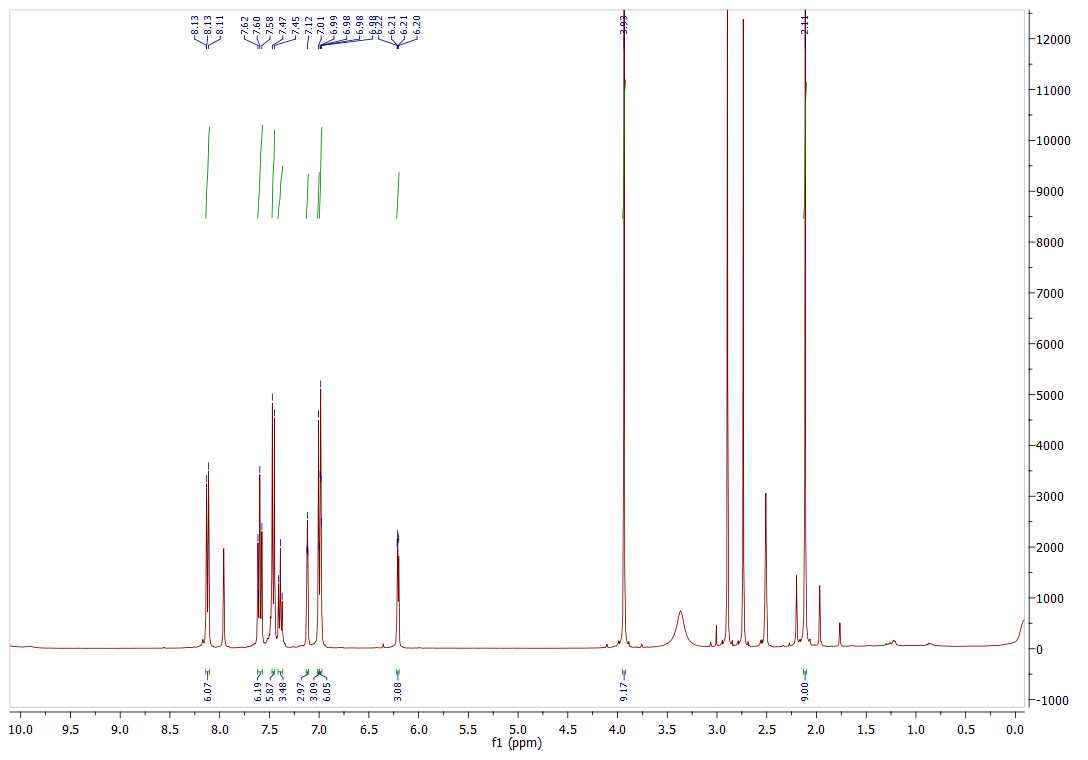

**Figure S51:** ^1^H NMR spectrum of 4,4',4''-(((1,3,5-triazine-2,4,6-triyl)tris(oxy))tris(benzene-4,1-diyl))tris(3-methyl-6-(1-methyl-1*H*-pyrrol-2-yl)-1-phenyl-1*H*-pyrazolo[3,4-*b*]pyridine-5-carbonitrile) (18a)


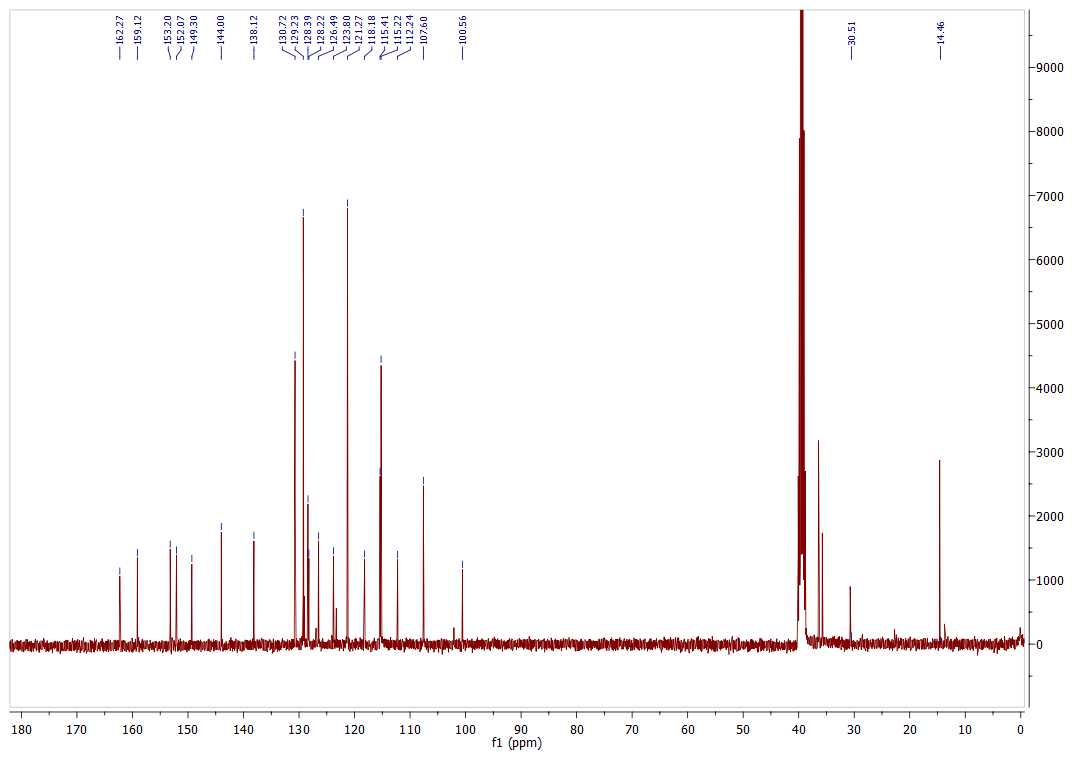

**Figure S52:** ^13^C NMR spectrum of 4,4',4''-(((1,3,5-triazine-2,4,6-triyl)tris(oxy))tris(benzene-4,1-diyl))tris(3-methyl-6-(1-methyl-1*H*-pyrrol-2-yl)-1-phenyl-1*H*-pyrazolo[3,4-*b*]pyridine-5-carbonitrile) (18a)
